# Supplementary material for: Comparative multiomics analysis of cell physiological state after culture in a basket bioreactor
Source: Sci Rep. 2022 Nov 23;12:20161. doi: 10.1038/s41598-022-24687-4 (PMC9686226; doi:10.1038/s41598-022-24687-4)
Supplement: Supplementary file 1 — Supplementary Information 1. [file 41598_2022_24687_MOESM1_ESM.zip › raw data/Transcriptomic raw data/6.Enrichment/1.GO/TE1vsTC1/GSEA/TE1/gsea_report_for_TE1_1656407547197.html]

Report for TE1 1656407547197 [GSEA]


| GS   follow link to MSigDB | GS DETAILS | SIZE | ES | NES | NOM p-val | FDR q-val | FWER p-val | RANK AT MAX | LEADING EDGE || 1 | ORGANIC\_ANION\_TRANSPORT(GO:0015711) | Details ... | 18 | 0.65 | 2.20 | 0.000 | 0.471 | 0.000 | 5165 | tags=61%, list=23%, signal=79% |
| 2 | ORGANIC\_ACID\_TRANSPORT(GO:0015849) | Details ... | 18 | 0.65 | 2.20 | 0.000 | 0.235 | 0.000 | 5165 | tags=61%, list=23%, signal=79% |
| 3 | CARBOXYLIC\_ACID\_TRANSPORT(GO:0046942) | Details ... | 18 | 0.65 | 2.20 | 0.000 | 0.157 | 0.000 | 5165 | tags=61%, list=23%, signal=79% |
| 4 | ORGANIC\_ANION\_TRANSMEMBRANE\_TRANSPORTER\_ACTIVITY(GO:0008514) | Details ... | 16 | 0.72 | 2.10 | 0.000 | 0.157 | 0.157 | 5165 | tags=75%, list=23%, signal=97% |
| 5 | RESPONSE\_TO\_HORMONE(GO:0009725) | Details ... | 46 | 0.50 | 2.08 | 0.000 | 0.157 | 0.157 | 6169 | tags=46%, list=28%, signal=63% |
| 6 | STEROID\_HORMONE\_MEDIATED\_SIGNALING\_PATHWAY(GO:0043401) | Details ... | 44 | 0.50 | 2.07 | 0.000 | 0.342 | 0.320 | 6169 | tags=45%, list=28%, signal=63% |
| 7 | HORMONE\_MEDIATED\_SIGNALING\_PATHWAY(GO:0009755) | Details ... | 44 | 0.50 | 2.07 | 0.000 | 0.293 | 0.320 | 6169 | tags=45%, list=28%, signal=63% |
| 8 | RESPONSE\_TO\_LIPID(GO:0033993) | Details ... | 44 | 0.50 | 2.07 | 0.000 | 0.257 | 0.320 | 6169 | tags=45%, list=28%, signal=63% |
| 9 | RESPONSE\_TO\_STEROID\_HORMONE(GO:0048545) | Details ... | 44 | 0.50 | 2.07 | 0.000 | 0.228 | 0.320 | 6169 | tags=45%, list=28%, signal=63% |
| 10 | CELLULAR\_RESPONSE\_TO\_STEROID\_HORMONE\_STIMULUS(GO:0071383) | Details ... | 44 | 0.50 | 2.07 | 0.000 | 0.205 | 0.320 | 6169 | tags=45%, list=28%, signal=63% |
| 11 | CELLULAR\_RESPONSE\_TO\_LIPID(GO:0071396) | Details ... | 44 | 0.50 | 2.07 | 0.000 | 0.187 | 0.320 | 6169 | tags=45%, list=28%, signal=63% |
| 12 | CELLULAR\_RESPONSE\_TO\_ORGANIC\_CYCLIC\_COMPOUND(GO:0071407) | Details ... | 44 | 0.50 | 2.07 | 0.000 | 0.171 | 0.320 | 6169 | tags=45%, list=28%, signal=63% |
| 13 | ORGANIC\_ACID\_TRANSMEMBRANE\_TRANSPORTER\_ACTIVITY(GO:0005342) | Details ... | 15 | 0.71 | 2.04 | 0.000 | 0.195 | 0.320 | 5165 | tags=73%, list=23%, signal=95% |
| 14 | CARBOXYLIC\_ACID\_TRANSMEMBRANE\_TRANSPORTER\_ACTIVITY(GO:0046943) | Details ... | 15 | 0.71 | 2.04 | 0.000 | 0.181 | 0.320 | 5165 | tags=73%, list=23%, signal=95% |
| 15 | CELLULAR\_RESPONSE\_TO\_HORMONE\_STIMULUS(GO:0032870) | Details ... | 45 | 0.49 | 2.03 | 0.000 | 0.179 | 0.320 | 6169 | tags=44%, list=28%, signal=61% |
| 16 | RESPONSE\_TO\_ENDOGENOUS\_STIMULUS(GO:0009719) | Details ... | 54 | 0.47 | 1.97 | 0.000 | 0.191 | 0.494 | 6169 | tags=43%, list=28%, signal=59% |
| 17 | PROTEIN\_HOMOOLIGOMERIZATION(GO:0051260) | Details ... | 41 | 0.54 | 1.96 | 0.000 | 0.201 | 0.494 | 3770 | tags=24%, list=17%, signal=29% |
| 18 | CELLULAR\_RESPONSE\_TO\_ENDOGENOUS\_STIMULUS(GO:0071495) | Details ... | 53 | 0.47 | 1.94 | 0.000 | 0.199 | 0.494 | 6169 | tags=42%, list=28%, signal=57% |
| 19 | CELLULAR\_RESPONSE\_TO\_CHEMICAL\_STIMULUS(GO:0070887) | Details ... | 54 | 0.46 | 1.91 | 0.000 | 0.205 | 0.494 | 6169 | tags=41%, list=28%, signal=56% |
| 20 | CELLULAR\_RESPONSE\_TO\_ORGANIC\_SUBSTANCE(GO:0071310) | Details ... | 54 | 0.46 | 1.91 | 0.000 | 0.195 | 0.494 | 6169 | tags=41%, list=28%, signal=56% |
| 21 | NITROGEN\_COMPOUND\_TRANSPORT(GO:0071705) | Details ... | 142 | 0.37 | 1.90 | 0.000 | 0.193 | 0.494 | 10225 | tags=67%, list=46%, signal=122% |
| 22 | TRANSLATION\_FACTOR\_ACTIVITY\_\_RNA\_BINDING(GO:0008135) | Details ... | 46 | 0.51 | 1.89 | 0.000 | 0.191 | 0.494 | 5448 | tags=52%, list=24%, signal=69% |
| 23 | CELLULAR\_AMINO\_ACID\_METABOLIC\_PROCESS(GO:0006520) | Details ... | 71 | 0.46 | 1.89 | 0.000 | 0.190 | 0.494 | 3974 | tags=38%, list=18%, signal=46% |
| 24 | INTRACELLULAR\_PROTEIN\_TRANSPORT(GO:0006886) | Details ... | 107 | 0.37 | 1.86 | 0.000 | 0.204 | 0.494 | 10225 | tags=69%, list=46%, signal=127% |
| 25 | PROTEIN\_TRANSPORT(GO:0015031) | Details ... | 122 | 0.37 | 1.86 | 0.000 | 0.208 | 0.494 | 10225 | tags=70%, list=46%, signal=127% |
| 26 | PEPTIDE\_TRANSPORT(GO:0015833) | Details ... | 122 | 0.37 | 1.86 | 0.000 | 0.200 | 0.494 | 10225 | tags=70%, list=46%, signal=127% |
| 27 | PROTEIN\_COMPLEX\_OLIGOMERIZATION(GO:0051259) | Details ... | 49 | 0.48 | 1.86 | 0.000 | 0.199 | 0.494 | 3770 | tags=20%, list=17%, signal=24% |
| 28 | AMIDE\_TRANSPORT(GO:0042886) | Details ... | 123 | 0.36 | 1.86 | 0.000 | 0.197 | 0.494 | 10225 | tags=69%, list=46%, signal=126% |
| 29 | ESTABLISHMENT\_OF\_PROTEIN\_LOCALIZATION(GO:0045184) | Details ... | 125 | 0.37 | 1.85 | 0.000 | 0.196 | 0.494 | 10225 | tags=70%, list=46%, signal=127% |
| 30 | PROTEOLYSIS\_INVOLVED\_IN\_CELLULAR\_PROTEIN\_CATABOLIC\_PROCESS(GO:0051603) | Details ... | 51 | 0.44 | 1.85 | 0.000 | 0.200 | 0.494 | 10943 | tags=82%, list=49%, signal=161% |
| 31 | CELLULAR\_PROTEIN\_CATABOLIC\_PROCESS(GO:0044257) | Details ... | 51 | 0.44 | 1.85 | 0.000 | 0.193 | 0.494 | 10943 | tags=82%, list=49%, signal=161% |
| 32 | ESTABLISHMENT\_OF\_LOCALIZATION\_IN\_CELL(GO:0051649) | Details ... | 127 | 0.35 | 1.84 | 0.000 | 0.192 | 0.494 | 10052 | tags=66%, list=45%, signal=119% |
| 33 | NUCLEAR\_PART(GO:0044428) | Details ... | 107 | 0.35 | 1.83 | 0.000 | 0.191 | 0.494 | 11985 | tags=77%, list=53%, signal=164% |
| 34 | INTRACELLULAR\_TRANSPORT(GO:0046907) | Details ... | 125 | 0.35 | 1.83 | 0.000 | 0.190 | 0.494 | 10052 | tags=66%, list=45%, signal=120% |
| 35 | PROTEIN\_CATABOLIC\_PROCESS(GO:0030163) | Details ... | 54 | 0.43 | 1.82 | 0.000 | 0.195 | 0.494 | 10943 | tags=81%, list=49%, signal=159% |
| 36 | TRANSLATIONAL\_INITIATION(GO:0006413) | Details ... | 23 | 0.49 | 1.82 | 0.000 | 0.200 | 0.494 | 6058 | tags=52%, list=27%, signal=71% |
| 37 | PROTEIN\_LOCALIZATION(GO:0008104) | Details ... | 132 | 0.36 | 1.81 | 0.000 | 0.210 | 0.494 | 10225 | tags=69%, list=46%, signal=126% |
| 38 | NUCLEOPLASM(GO:0005654) | Details ... | 55 | 0.41 | 1.80 | 0.000 | 0.213 | 0.494 | 11948 | tags=87%, list=53%, signal=187% |
| 39 | NUCLEOPLASM\_PART(GO:0044451) | Details ... | 55 | 0.41 | 1.80 | 0.000 | 0.207 | 0.494 | 11948 | tags=87%, list=53%, signal=187% |
| 40 | CELLULAR\_PROTEIN\_LOCALIZATION(GO:0034613) | Details ... | 118 | 0.36 | 1.80 | 0.000 | 0.215 | 0.494 | 10052 | tags=68%, list=45%, signal=122% |
| 41 | CELLULAR\_MACROMOLECULE\_LOCALIZATION(GO:0070727) | Details ... | 118 | 0.36 | 1.80 | 0.000 | 0.210 | 0.494 | 10052 | tags=68%, list=45%, signal=122% |
| 42 | ORGANIC\_SUBSTANCE\_TRANSPORT(GO:0071702) | Details ... | 170 | 0.34 | 1.78 | 0.000 | 0.209 | 0.494 | 10225 | tags=61%, list=46%, signal=112% |
| 43 | PROTEIN\_TARGETING(GO:0006605) | Details ... | 25 | 0.51 | 1.78 | 0.000 | 0.212 | 0.494 | 4938 | tags=60%, list=22%, signal=77% |
| 44 | NUCLEUS(GO:0005634) | Details ... | 549 | 0.33 | 1.77 | 0.000 | 0.211 | 0.494 | 10079 | tags=58%, list=45%, signal=103% |
| 45 | GROWTH\_FACTOR\_ACTIVITY(GO:0008083) | Details ... | 58 | 0.54 | 1.77 | 0.000 | 0.210 | 0.494 | 3790 | tags=31%, list=17%, signal=37% |
| 46 | MACROMOLECULE\_CATABOLIC\_PROCESS(GO:0009057) | Details ... | 63 | 0.41 | 1.77 | 0.000 | 0.209 | 0.494 | 10626 | tags=78%, list=47%, signal=148% |
| 47 | MEMBRANE\_ENCLOSED\_LUMEN(GO:0031974) | Details ... | 90 | 0.36 | 1.77 | 0.000 | 0.214 | 0.494 | 11985 | tags=78%, list=53%, signal=167% |
| 48 | ORGANELLE\_LUMEN(GO:0043233) | Details ... | 90 | 0.36 | 1.77 | 0.000 | 0.210 | 0.494 | 11985 | tags=78%, list=53%, signal=167% |
| 49 | INTRACELLULAR\_ORGANELLE\_LUMEN(GO:0070013) | Details ... | 90 | 0.36 | 1.77 | 0.000 | 0.206 | 0.494 | 11985 | tags=78%, list=53%, signal=167% |
| 50 | GROWTH(GO:0040007) | Details ... | 38 | 0.58 | 1.76 | 0.000 | 0.205 | 0.494 | 2906 | tags=26%, list=13%, signal=30% |
| 51 | TRANSLATION\_INITIATION\_FACTOR\_ACTIVITY(GO:0003743) |  | 25 | 0.56 | 1.76 | 0.000 | 0.204 | 0.494 | 5448 | tags=60%, list=24%, signal=79% |
| 52 | RNA\_PROCESSING(GO:0006396) |  | 126 | 0.36 | 1.76 | 0.000 | 0.203 | 0.494 | 10343 | tags=69%, list=46%, signal=128% |
| 53 | RNA\_BINDING(GO:0003723) |  | 269 | 0.40 | 1.76 | 0.000 | 0.202 | 0.494 | 8756 | tags=62%, list=39%, signal=100% |
| 54 | TRANSFERASE\_COMPLEX(GO:1990234) |  | 37 | 0.44 | 1.75 | 0.000 | 0.201 | 0.494 | 10825 | tags=84%, list=48%, signal=162% |
| 55 | MEMBRANE\_BOUNDED\_ORGANELLE(GO:0043227) |  | 804 | 0.32 | 1.74 | 0.000 | 0.200 | 0.494 | 10031 | tags=57%, list=45%, signal=100% |
| 56 | INTRACELLULAR\_MEMBRANE\_BOUNDED\_ORGANELLE(GO:0043231) |  | 787 | 0.32 | 1.73 | 0.000 | 0.200 | 0.494 | 10079 | tags=57%, list=45%, signal=100% |
| 57 | PROTEIN\_MODIFICATION\_BY\_SMALL\_PROTEIN\_CONJUGATION(GO:0032446) |  | 20 | 0.42 | 1.73 | 0.000 | 0.199 | 0.494 | 7217 | tags=55%, list=32%, signal=81% |
| 58 | ORGANELLE\_ENVELOPE(GO:0031967) |  | 81 | 0.40 | 1.71 | 0.000 | 0.214 | 0.654 | 9132 | tags=64%, list=41%, signal=108% |
| 59 | ENVELOPE(GO:0031975) |  | 81 | 0.40 | 1.71 | 0.000 | 0.210 | 0.654 | 9132 | tags=64%, list=41%, signal=108% |
| 60 | NUCLEAR\_LUMEN(GO:0031981) |  | 81 | 0.36 | 1.71 | 0.000 | 0.220 | 0.654 | 11985 | tags=78%, list=53%, signal=167% |
| 61 | CATALYTIC\_ACTIVITY\_\_ACTING\_ON\_RNA(GO:0140098) |  | 128 | 0.37 | 1.70 | 0.000 | 0.219 | 0.654 | 11836 | tags=78%, list=53%, signal=165% |
| 62 | PROTEIN\_UBIQUITINATION(GO:0016567) |  | 19 | 0.40 | 1.70 | 0.000 | 0.218 | 0.654 | 7217 | tags=53%, list=32%, signal=78% |
| 63 | NCRNA\_METABOLIC\_PROCESS(GO:0034660) |  | 97 | 0.40 | 1.69 | 0.000 | 0.227 | 0.654 | 10161 | tags=73%, list=45%, signal=133% |
| 64 | TRANSCRIPTION\_BY\_RNA\_POLYMERASE\_II(GO:0006366) |  | 46 | 0.37 | 1.68 | 0.000 | 0.226 | 0.654 | 9893 | tags=70%, list=44%, signal=124% |
| 65 | TRNA\_METABOLIC\_PROCESS(GO:0006399) |  | 75 | 0.42 | 1.68 | 0.000 | 0.224 | 0.654 | 10161 | tags=76%, list=45%, signal=139% |
| 66 | CATALYTIC\_ACTIVITY\_\_ACTING\_ON\_A\_TRNA(GO:0140101) |  | 62 | 0.42 | 1.68 | 0.000 | 0.223 | 0.654 | 10161 | tags=76%, list=45%, signal=138% |
| 67 | CELLULAR\_MACROMOLECULE\_CATABOLIC\_PROCESS(GO:0044265) |  | 60 | 0.42 | 1.67 | 0.000 | 0.222 | 0.654 | 10626 | tags=78%, list=47%, signal=149% |
| 68 | BOUNDING\_MEMBRANE\_OF\_ORGANELLE(GO:0098588) |  | 62 | 0.38 | 1.67 | 0.000 | 0.221 | 0.654 | 6965 | tags=50%, list=31%, signal=72% |
| 69 | PROTEIN\_CONTAINING\_COMPLEX\_SUBUNIT\_ORGANIZATION(GO:0043933) |  | 140 | 0.37 | 1.67 | 0.000 | 0.221 | 0.654 | 5966 | tags=33%, list=27%, signal=45% |
| 70 | TRNA\_AMINOACYLATION\_FOR\_PROTEIN\_TRANSLATION(GO:0006418) |  | 40 | 0.56 | 1.67 | 0.000 | 0.228 | 0.654 | 3974 | tags=52%, list=18%, signal=64% |
| 71 | RESPONSE\_TO\_ORGANIC\_CYCLIC\_COMPOUND(GO:0014070) |  | 52 | 0.48 | 1.67 | 0.000 | 0.227 | 0.654 | 1792 | tags=17%, list=8%, signal=19% |
| 72 | MITOCHONDRIAL\_MEMBRANE(GO:0031966) |  | 70 | 0.42 | 1.67 | 0.000 | 0.226 | 0.654 | 9132 | tags=66%, list=41%, signal=111% |
| 73 | CARBOXYLIC\_ACID\_METABOLIC\_PROCESS(GO:0019752) |  | 129 | 0.38 | 1.66 | 0.000 | 0.225 | 0.654 | 4880 | tags=35%, list=22%, signal=44% |
| 74 | AMINO\_ACID\_ACTIVATION(GO:0043038) |  | 42 | 0.54 | 1.66 | 0.000 | 0.226 | 0.654 | 3974 | tags=50%, list=18%, signal=61% |
| 75 | TRNA\_AMINOACYLATION(GO:0043039) |  | 42 | 0.54 | 1.66 | 0.000 | 0.223 | 0.654 | 3974 | tags=50%, list=18%, signal=61% |
| 76 | MITOCHONDRIAL\_PART(GO:0044429) |  | 81 | 0.40 | 1.66 | 0.000 | 0.222 | 0.654 | 9132 | tags=63%, list=41%, signal=106% |
| 77 | ORGANIC\_ACID\_METABOLIC\_PROCESS(GO:0006082) |  | 130 | 0.38 | 1.65 | 0.000 | 0.226 | 0.654 | 4880 | tags=35%, list=22%, signal=44% |
| 78 | OXOACID\_METABOLIC\_PROCESS(GO:0043436) |  | 130 | 0.38 | 1.65 | 0.000 | 0.223 | 0.654 | 4880 | tags=35%, list=22%, signal=44% |
| 79 | MITOCHONDRIAL\_MEMBRANE\_PART(GO:0044455) |  | 48 | 0.44 | 1.65 | 0.000 | 0.223 | 0.654 | 8663 | tags=67%, list=39%, signal=108% |
| 80 | PROTEIN\_CONTAINING\_COMPLEX\_ASSEMBLY(GO:0065003) |  | 132 | 0.37 | 1.65 | 0.000 | 0.222 | 0.654 | 5966 | tags=32%, list=27%, signal=43% |
| 81 | LIGASE\_ACTIVITY(GO:0016874) |  | 81 | 0.45 | 1.65 | 0.000 | 0.221 | 0.654 | 7962 | tags=63%, list=36%, signal=97% |
| 82 | PEPTIDYL\_AMINO\_ACID\_MODIFICATION(GO:0018193) |  | 75 | 0.38 | 1.65 | 0.000 | 0.220 | 0.654 | 11365 | tags=75%, list=51%, signal=151% |
| 83 | MACROMOLECULE\_LOCALIZATION(GO:0033036) |  | 155 | 0.32 | 1.64 | 0.000 | 0.219 | 0.654 | 10225 | tags=61%, list=46%, signal=112% |
| 84 | CELLULAR\_LOCALIZATION(GO:0051641) |  | 150 | 0.35 | 1.64 | 0.000 | 0.219 | 0.654 | 9799 | tags=65%, list=44%, signal=114% |
| 85 | CHROMATIN\_ORGANIZATION(GO:0006325) |  | 51 | 0.37 | 1.64 | 0.000 | 0.218 | 0.654 | 9893 | tags=61%, list=44%, signal=109% |
| 86 | PROTEIN\_LOCALIZATION\_TO\_ENDOPLASMIC\_RETICULUM(GO:0070972) |  | 18 | 0.54 | 1.64 | 0.000 | 0.219 | 0.829 | 7232 | tags=78%, list=32%, signal=115% |
| 87 | RESPONSE\_TO\_ORGANIC\_SUBSTANCE(GO:0010033) |  | 65 | 0.44 | 1.64 | 0.000 | 0.219 | 0.829 | 6169 | tags=37%, list=28%, signal=51% |
| 88 | AMINOACYL\_TRNA\_LIGASE\_ACTIVITY(GO:0004812) |  | 42 | 0.53 | 1.63 | 0.000 | 0.224 | 0.829 | 3974 | tags=50%, list=18%, signal=61% |
| 89 | LIGASE\_ACTIVITY\_\_FORMING\_CARBON\_OXYGEN\_BONDS(GO:0016875) |  | 42 | 0.53 | 1.63 | 0.000 | 0.222 | 0.829 | 3974 | tags=50%, list=18%, signal=61% |
| 90 | PROTEIN\_DNA\_COMPLEX\_ASSEMBLY(GO:0065004) |  | 35 | 0.46 | 1.63 | 0.000 | 0.227 | 0.829 | 3195 | tags=29%, list=14%, signal=33% |
| 91 | PROTEIN\_DNA\_COMPLEX\_SUBUNIT\_ORGANIZATION(GO:0071824) |  | 35 | 0.46 | 1.63 | 0.000 | 0.224 | 0.829 | 3195 | tags=29%, list=14%, signal=33% |
| 92 | PROTON\_TRANSMEMBRANE\_TRANSPORT(GO:1902600) |  | 52 | 0.40 | 1.62 | 0.000 | 0.228 | 0.829 | 8663 | tags=62%, list=39%, signal=100% |
| 93 | PROTON\_TRANSPORTING\_TWO\_SECTOR\_ATPASE\_COMPLEX(GO:0016469) |  | 43 | 0.43 | 1.62 | 0.000 | 0.227 | 0.829 | 8663 | tags=65%, list=39%, signal=106% |
| 94 | LIGASE\_ACTIVITY\_\_FORMING\_CARBON\_NITROGEN\_BONDS(GO:0016879) |  | 23 | 0.49 | 1.62 | 0.000 | 0.228 | 0.829 | 7942 | tags=65%, list=35%, signal=101% |
| 95 | TRANSCRIPTION\_COFACTOR\_ACTIVITY(GO:0003712) |  | 32 | 0.39 | 1.62 | 0.000 | 0.231 | 0.829 | 7687 | tags=63%, list=34%, signal=95% |
| 96 | TRANSCRIPTION\_FACTOR\_ACTIVITY\_\_PROTEIN\_BINDING(GO:0000988) |  | 32 | 0.39 | 1.62 | 0.000 | 0.228 | 0.829 | 7687 | tags=63%, list=34%, signal=95% |
| 97 | TRANSCRIPTION\_FACTOR\_ACTIVITY\_\_TRANSCRIPTION\_FACTOR\_BINDING(GO:0000989) |  | 32 | 0.39 | 1.62 | 0.000 | 0.226 | 0.829 | 7687 | tags=63%, list=34%, signal=95% |
| 98 | MITOCHONDRIAL\_ENVELOPE(GO:0005740) |  | 74 | 0.40 | 1.62 | 0.000 | 0.228 | 0.829 | 9132 | tags=64%, list=41%, signal=107% |
| 99 | RESPONSE\_TO\_CHEMICAL(GO:0042221) |  | 76 | 0.43 | 1.61 | 0.000 | 0.231 | 0.829 | 4981 | tags=29%, list=22%, signal=37% |
| 100 | POSITIVE\_REGULATION\_OF\_CELLULAR\_COMPONENT\_ORGANIZATION(GO:0051130) |  | 17 | 0.45 | 1.61 | 0.000 | 0.230 | 0.829 | 11721 | tags=88%, list=52%, signal=185% |
| 101 | SECONDARY\_ACTIVE\_TRANSMEMBRANE\_TRANSPORTER\_ACTIVITY(GO:0015291) |  | 38 | 0.41 | 1.61 | 0.000 | 0.230 | 0.829 | 5325 | tags=37%, list=24%, signal=48% |
| 102 | NUCLEOSOME\_ASSEMBLY(GO:0006334) |  | 28 | 0.52 | 1.60 | 0.000 | 0.238 | 0.829 | 3195 | tags=32%, list=14%, signal=37% |
| 103 | NUCLEOSOME\_ORGANIZATION(GO:0034728) |  | 28 | 0.52 | 1.60 | 0.000 | 0.236 | 0.829 | 3195 | tags=32%, list=14%, signal=37% |
| 104 | CHROMATIN\_ASSEMBLY(GO:0031497) |  | 28 | 0.52 | 1.60 | 0.000 | 0.234 | 0.829 | 3195 | tags=32%, list=14%, signal=37% |
| 105 | OXIDOREDUCTASE\_ACTIVITY\_\_ACTING\_ON\_CH\_OH\_GROUP\_OF\_DONORS(GO:0016614) |  | 65 | 0.39 | 1.60 | 0.000 | 0.233 | 0.829 | 6798 | tags=46%, list=30%, signal=66% |
| 106 | PROTON\_TRANSPORTING\_TWO\_SECTOR\_ATPASE\_COMPLEX\_\_PROTON\_TRANSPORTING\_DOMAIN(GO:0033177) |  | 26 | 0.48 | 1.60 | 0.000 | 0.234 | 0.829 | 7526 | tags=69%, list=34%, signal=104% |
| 107 | CELLULAR\_COMPONENT\_ASSEMBLY(GO:0022607) |  | 149 | 0.35 | 1.60 | 0.000 | 0.233 | 0.829 | 7401 | tags=39%, list=33%, signal=58% |
| 108 | COLLAGEN\_TRIMER(GO:0005581) |  | 16 | 0.68 | 1.60 | 0.000 | 0.233 | 0.829 | 2519 | tags=56%, list=11%, signal=63% |
| 109 | EXTRACELLULAR\_MATRIX\_STRUCTURAL\_CONSTITUENT(GO:0005201) |  | 17 | 0.68 | 1.60 | 0.000 | 0.232 | 0.829 | 2519 | tags=53%, list=11%, signal=60% |
| 110 | ORGANELLE\_MEMBRANE(GO:0031090) |  | 109 | 0.38 | 1.60 | 0.000 | 0.231 | 0.829 | 9733 | tags=66%, list=43%, signal=116% |
| 111 | CELLULAR\_COMPONENT\_ORGANIZATION\_OR\_BIOGENESIS(GO:0071840) |  | 356 | 0.30 | 1.60 | 0.000 | 0.231 | 0.829 | 12453 | tags=67%, list=56%, signal=149% |
| 112 | CYSTEINE\_TYPE\_PEPTIDASE\_ACTIVITY(GO:0008234) |  | 91 | 0.33 | 1.60 | 0.000 | 0.230 | 0.829 | 10078 | tags=60%, list=45%, signal=109% |
| 113 | CELLULAR\_COMPONENT\_BIOGENESIS(GO:0044085) |  | 191 | 0.33 | 1.60 | 0.000 | 0.229 | 0.829 | 9883 | tags=53%, list=44%, signal=95% |
| 114 | WHOLE\_MEMBRANE(GO:0098805) |  | 48 | 0.39 | 1.59 | 0.000 | 0.229 | 0.829 | 10225 | tags=69%, list=46%, signal=126% |
| 115 | INORGANIC\_ION\_TRANSMEMBRANE\_TRANSPORT(GO:0098660) |  | 65 | 0.39 | 1.59 | 0.000 | 0.228 | 0.829 | 7913 | tags=55%, list=35%, signal=85% |
| 116 | OXIDOREDUCTASE\_ACTIVITY\_\_ACTING\_ON\_THE\_CH\_OH\_GROUP\_OF\_DONORS\_\_NAD\_OR\_NADP\_AS\_ACCEPTOR(GO:0016616) |  | 55 | 0.41 | 1.59 | 0.000 | 0.227 | 0.829 | 6798 | tags=49%, list=30%, signal=70% |
| 117 | CELLULAR\_PROTEIN\_METABOLIC\_PROCESS(GO:0044267) |  | 1467 | 0.33 | 1.59 | 0.000 | 0.235 | 0.829 | 10813 | tags=60%, list=48%, signal=109% |
| 118 | DNA\_PACKAGING(GO:0006323) |  | 29 | 0.50 | 1.59 | 0.000 | 0.234 | 0.829 | 3195 | tags=31%, list=14%, signal=36% |
| 119 | ION\_TRANSMEMBRANE\_TRANSPORT(GO:0034220) |  | 89 | 0.40 | 1.59 | 0.000 | 0.233 | 0.829 | 8405 | tags=54%, list=38%, signal=86% |
| 120 | PROTEIN\_KINASE\_REGULATOR\_ACTIVITY(GO:0019887) |  | 19 | 0.56 | 1.58 | 0.000 | 0.238 | 0.829 | 3971 | tags=53%, list=18%, signal=64% |
| 121 | INTRACELLULAR\_PART(GO:0044424) |  | 1799 | 0.32 | 1.58 | 0.000 | 0.237 | 0.829 | 11130 | tags=60%, list=50%, signal=111% |
| 122 | UNFOLDED\_PROTEIN\_BINDING(GO:0051082) |  | 28 | 0.44 | 1.58 | 0.000 | 0.240 | 0.829 | 8118 | tags=61%, list=36%, signal=95% |
| 123 | ORGANELLE(GO:0043226) |  | 1548 | 0.33 | 1.58 | 0.000 | 0.239 | 0.829 | 9805 | tags=54%, list=44%, signal=89% |
| 124 | TRANSFERASE\_COMPLEX\_\_TRANSFERRING\_PHOSPHORUS\_CONTAINING\_GROUPS(GO:0061695) |  | 20 | 0.47 | 1.58 | 0.000 | 0.239 | 0.829 | 9893 | tags=80%, list=44%, signal=143% |
| 125 | INTRACELLULAR\_ORGANELLE(GO:0043229) |  | 1543 | 0.33 | 1.58 | 0.000 | 0.238 | 0.829 | 9805 | tags=54%, list=44%, signal=89% |
| 126 | CELLULAR\_COMPONENT\_ORGANIZATION(GO:0016043) |  | 314 | 0.30 | 1.57 | 0.000 | 0.237 | 0.829 | 12984 | tags=70%, list=58%, signal=164% |
| 127 | NUCLEOCYTOPLASMIC\_TRANSPORT(GO:0006913) |  | 15 | 0.41 | 1.57 | 0.000 | 0.240 | 0.829 | 9717 | tags=67%, list=43%, signal=118% |
| 128 | NUCLEAR\_TRANSPORT(GO:0051169) |  | 15 | 0.41 | 1.57 | 0.000 | 0.238 | 0.829 | 9717 | tags=67%, list=43%, signal=118% |
| 129 | SEQUENCE\_SPECIFIC\_DNA\_BINDING(GO:0043565) |  | 171 | 0.35 | 1.57 | 0.000 | 0.237 | 0.829 | 7555 | tags=42%, list=34%, signal=62% |
| 130 | CHROMATIN\_ASSEMBLY\_OR\_DISASSEMBLY(GO:0006333) |  | 30 | 0.49 | 1.57 | 0.000 | 0.237 | 0.829 | 3195 | tags=30%, list=14%, signal=35% |
| 131 | MRNA\_PROCESSING(GO:0006397) |  | 41 | 0.40 | 1.56 | 0.000 | 0.236 | 0.829 | 10321 | tags=68%, list=46%, signal=126% |
| 132 | ESTABLISHMENT\_OF\_PROTEIN\_LOCALIZATION\_TO\_ORGANELLE(GO:0072594) |  | 29 | 0.48 | 1.56 | 0.000 | 0.237 | 0.829 | 10052 | tags=83%, list=45%, signal=150% |
| 133 | DNA\_DIRECTED\_RNA\_POLYMERASE\_II\_\_HOLOENZYME(GO:0016591) |  | 16 | 0.46 | 1.56 | 0.000 | 0.236 | 0.829 | 8014 | tags=69%, list=36%, signal=107% |
| 134 | MULTICELLULAR\_ORGANISM\_DEVELOPMENT(GO:0007275) |  | 65 | 0.43 | 1.56 | 0.000 | 0.236 | 0.829 | 6898 | tags=42%, list=31%, signal=60% |
| 135 | HELICASE\_ACTIVITY(GO:0004386) |  | 37 | 0.41 | 1.56 | 0.000 | 0.235 | 0.829 | 8738 | tags=62%, list=39%, signal=102% |
| 136 | PHOSPHOLIPID\_BINDING(GO:0005543) |  | 69 | 0.33 | 1.56 | 0.000 | 0.235 | 0.829 | 9466 | tags=59%, list=42%, signal=103% |
| 137 | ACTIN\_BINDING(GO:0003779) |  | 77 | 0.32 | 1.56 | 0.000 | 0.238 | 0.829 | 12781 | tags=77%, list=57%, signal=178% |
| 138 | DNA\_CONFORMATION\_CHANGE(GO:0071103) |  | 36 | 0.43 | 1.55 | 0.000 | 0.237 | 0.829 | 4157 | tags=31%, list=19%, signal=37% |
| 139 | PROTON\_TRANSMEMBRANE\_TRANSPORTER\_ACTIVITY(GO:0015078) |  | 93 | 0.37 | 1.55 | 0.000 | 0.239 | 0.829 | 9132 | tags=59%, list=41%, signal=99% |
| 140 | INORGANIC\_CATION\_TRANSMEMBRANE\_TRANSPORT(GO:0098662) |  | 64 | 0.38 | 1.55 | 0.000 | 0.240 | 0.829 | 7913 | tags=55%, list=35%, signal=84% |
| 141 | ANATOMICAL\_STRUCTURE\_DEVELOPMENT(GO:0048856) |  | 71 | 0.42 | 1.55 | 0.000 | 0.239 | 0.829 | 6570 | tags=38%, list=29%, signal=54% |
| 142 | RIBOSOME\_BIOGENESIS(GO:0042254) |  | 43 | 0.34 | 1.55 | 0.000 | 0.248 | 0.829 | 9883 | tags=65%, list=44%, signal=116% |
| 143 | MRNA\_METABOLIC\_PROCESS(GO:0016071) |  | 48 | 0.37 | 1.55 | 0.000 | 0.247 | 0.829 | 10578 | tags=67%, list=47%, signal=126% |
| 144 | PROTEIN\_TYROSINE\_SERINE\_THREONINE\_PHOSPHATASE\_ACTIVITY(GO:0008138) |  | 36 | 0.36 | 1.55 | 0.000 | 0.247 | 0.829 | 12170 | tags=78%, list=54%, signal=170% |
| 145 | PHOSPHATIDYLINOSITOL\_BINDING(GO:0035091) |  | 54 | 0.34 | 1.54 | 0.000 | 0.253 | 0.829 | 9422 | tags=59%, list=42%, signal=102% |
| 146 | ORGANONITROGEN\_COMPOUND\_CATABOLIC\_PROCESS(GO:1901565) |  | 66 | 0.39 | 1.54 | 0.000 | 0.254 | 0.829 | 11137 | tags=77%, list=50%, signal=153% |
| 147 | DNA\_TEMPLATED\_TRANSCRIPTION\_\_INITIATION(GO:0006352) |  | 29 | 0.44 | 1.54 | 0.000 | 0.254 | 0.829 | 8362 | tags=66%, list=37%, signal=104% |
| 148 | CELLULAR\_LIPID\_METABOLIC\_PROCESS(GO:0044255) |  | 84 | 0.33 | 1.53 | 0.000 | 0.255 | 0.829 | 9542 | tags=55%, list=43%, signal=95% |
| 149 | CELL\_CYCLE(GO:0007049) |  | 60 | 0.32 | 1.53 | 0.000 | 0.254 | 0.829 | 10079 | tags=58%, list=45%, signal=106% |
| 150 | MITOCHONDRION(GO:0005739) |  | 116 | 0.34 | 1.53 | 0.000 | 0.258 | 0.829 | 8663 | tags=55%, list=39%, signal=89% |
| 151 | CHROMOSOME\_ORGANIZATION(GO:0051276) |  | 82 | 0.32 | 1.52 | 0.000 | 0.267 | 0.829 | 9893 | tags=56%, list=44%, signal=100% |
| 152 | ORGANONITROGEN\_COMPOUND\_METABOLIC\_PROCESS(GO:1901564) |  | 1954 | 0.32 | 1.51 | 0.000 | 0.266 | 0.829 | 10948 | tags=58%, list=49%, signal=104% |
| 153 | ORGANELLE\_ORGANIZATION(GO:0006996) |  | 194 | 0.30 | 1.51 | 0.000 | 0.265 | 0.829 | 12972 | tags=74%, list=58%, signal=174% |
| 154 | INTRACELLULAR\_ORGANELLE\_PART(GO:0044446) |  | 509 | 0.30 | 1.51 | 0.000 | 0.265 | 0.829 | 12050 | tags=65%, list=54%, signal=137% |
| 155 | ORGANELLE\_PART(GO:0044422) |  | 513 | 0.30 | 1.51 | 0.000 | 0.264 | 0.829 | 12050 | tags=65%, list=54%, signal=137% |
| 156 | PROTEIN\_FOLDING(GO:0006457) |  | 109 | 0.40 | 1.51 | 0.000 | 0.265 | 0.829 | 8304 | tags=57%, list=37%, signal=90% |
| 157 | SMALL\_MOLECULE\_BINDING(GO:0036094) |  | 1579 | 0.30 | 1.51 | 0.000 | 0.265 | 0.829 | 9863 | tags=54%, list=44%, signal=89% |
| 158 | NUCLEOTIDE\_BINDING(GO:0000166) |  | 1522 | 0.30 | 1.51 | 0.000 | 0.268 | 0.829 | 9863 | tags=54%, list=44%, signal=89% |
| 159 | NUCLEOSIDE\_PHOSPHATE\_BINDING(GO:1901265) |  | 1522 | 0.30 | 1.51 | 0.000 | 0.266 | 0.829 | 9863 | tags=54%, list=44%, signal=89% |
| 160 | CATION\_TRANSPORT(GO:0006812) |  | 177 | 0.35 | 1.50 | 0.000 | 0.266 | 0.829 | 8430 | tags=49%, list=38%, signal=78% |
| 161 | PROTEIN\_METABOLIC\_PROCESS(GO:0019538) |  | 1740 | 0.32 | 1.50 | 0.000 | 0.266 | 0.829 | 10813 | tags=58%, list=48%, signal=103% |
| 162 | GLYCOSAMINOGLYCAN\_BINDING(GO:0005539) |  | 18 | 0.36 | 1.50 | 0.000 | 0.265 | 0.829 | 4814 | tags=33%, list=21%, signal=42% |
| 163 | DEVELOPMENTAL\_PROCESS(GO:0032502) |  | 74 | 0.41 | 1.50 | 0.000 | 0.264 | 0.829 | 6570 | tags=36%, list=29%, signal=51% |
| 164 | VIRION(GO:0019012) |  | 17 | 0.57 | 1.50 | 0.000 | 0.265 | 0.829 | 2808 | tags=29%, list=13%, signal=34% |
| 165 | VIRION\_PART(GO:0044423) |  | 17 | 0.57 | 1.50 | 0.000 | 0.263 | 0.829 | 2808 | tags=29%, list=13%, signal=34% |
| 166 | CELL\_SURFACE\_RECEPTOR\_SIGNALING\_PATHWAY(GO:0007166) |  | 85 | 0.37 | 1.50 | 0.000 | 0.264 | 0.829 | 3079 | tags=22%, list=14%, signal=26% |
| 167 | ENDOMEMBRANE\_SYSTEM(GO:0012505) |  | 133 | 0.29 | 1.50 | 0.000 | 0.265 | 0.829 | 11638 | tags=64%, list=52%, signal=132% |
| 168 | NEGATIVE\_REGULATION\_OF\_CELLULAR\_METABOLIC\_PROCESS(GO:0031324) |  | 21 | 0.37 | 1.50 | 0.000 | 0.266 | 0.829 | 6852 | tags=48%, list=31%, signal=69% |
| 169 | NEGATIVE\_REGULATION\_OF\_NITROGEN\_COMPOUND\_METABOLIC\_PROCESS(GO:0051172) |  | 21 | 0.37 | 1.50 | 0.000 | 0.264 | 0.829 | 6852 | tags=48%, list=31%, signal=69% |
| 170 | RRNA\_PROCESSING(GO:0006364) |  | 24 | 0.37 | 1.50 | 0.000 | 0.266 | 0.829 | 5989 | tags=50%, list=27%, signal=68% |
| 171 | RRNA\_METABOLIC\_PROCESS(GO:0016072) |  | 24 | 0.37 | 1.50 | 0.000 | 0.264 | 0.829 | 5989 | tags=50%, list=27%, signal=68% |
| 172 | MITOCHONDRIAL\_PROTEIN\_COMPLEX(GO:0098798) |  | 44 | 0.43 | 1.50 | 0.000 | 0.265 | 0.829 | 7633 | tags=59%, list=34%, signal=89% |
| 173 | GOLGI\_SUBCOMPARTMENT(GO:0098791) |  | 16 | 0.41 | 1.50 | 0.000 | 0.264 | 0.829 | 4803 | tags=44%, list=21%, signal=56% |
| 174 | ORGANONITROGEN\_COMPOUND\_BIOSYNTHETIC\_PROCESS(GO:1901566) |  | 809 | 0.37 | 1.50 | 0.000 | 0.263 | 0.829 | 9441 | tags=57%, list=42%, signal=94% |
| 175 | GOLGI\_APPARATUS\_PART(GO:0044431) |  | 29 | 0.34 | 1.49 | 0.000 | 0.267 | 0.829 | 5081 | tags=41%, list=23%, signal=53% |
| 176 | CATALYTIC\_COMPLEX(GO:1902494) |  | 136 | 0.33 | 1.49 | 0.000 | 0.267 | 0.829 | 11127 | tags=66%, list=50%, signal=131% |
| 177 | VESICLE\_MEDIATED\_TRANSPORT(GO:0016192) |  | 105 | 0.29 | 1.49 | 0.000 | 0.269 | 0.829 | 11614 | tags=66%, list=52%, signal=136% |
| 178 | TRANSCRIPTION\_FACTOR\_COMPLEX(GO:0005667) |  | 30 | 0.42 | 1.49 | 0.000 | 0.268 | 0.829 | 9653 | tags=73%, list=43%, signal=129% |
| 179 | NUCLEIC\_ACID\_BINDING(GO:0003676) |  | 1797 | 0.28 | 1.48 | 0.000 | 0.274 | 1.000 | 10779 | tags=55%, list=48%, signal=97% |
| 180 | NCRNA\_PROCESSING(GO:0034470) |  | 57 | 0.34 | 1.48 | 0.000 | 0.274 | 1.000 | 12681 | tags=82%, list=57%, signal=190% |
| 181 | DNA\_DIRECTED\_RNA\_POLYMERASE\_COMPLEX(GO:0000428) |  | 18 | 0.47 | 1.48 | 0.000 | 0.276 | 1.000 | 9893 | tags=83%, list=44%, signal=149% |
| 182 | RNA\_POLYMERASE\_COMPLEX(GO:0030880) |  | 18 | 0.47 | 1.48 | 0.000 | 0.274 | 1.000 | 9893 | tags=83%, list=44%, signal=149% |
| 183 | NUCLEAR\_DNA\_DIRECTED\_RNA\_POLYMERASE\_COMPLEX(GO:0055029) |  | 18 | 0.47 | 1.48 | 0.000 | 0.273 | 1.000 | 9893 | tags=83%, list=44%, signal=149% |
| 184 | CELLULAR\_METABOLIC\_PROCESS(GO:0044237) |  | 3013 | 0.28 | 1.48 | 0.000 | 0.272 | 1.000 | 11180 | tags=57%, list=50%, signal=98% |
| 185 | SMALL\_MOLECULE\_METABOLIC\_PROCESS(GO:0044281) |  | 302 | 0.30 | 1.47 | 0.000 | 0.278 | 1.000 | 8130 | tags=45%, list=36%, signal=70% |
| 186 | SRP\_DEPENDENT\_COTRANSLATIONAL\_PROTEIN\_TARGETING\_TO\_MEMBRANE(GO:0006614) |  | 15 | 0.56 | 1.47 | 0.000 | 0.282 | 1.000 | 4938 | tags=67%, list=22%, signal=85% |
| 187 | PROTEIN\_TARGETING\_TO\_MEMBRANE(GO:0006612) |  | 15 | 0.56 | 1.47 | 0.000 | 0.280 | 1.000 | 4938 | tags=67%, list=22%, signal=85% |
| 188 | COTRANSLATIONAL\_PROTEIN\_TARGETING\_TO\_MEMBRANE(GO:0006613) |  | 15 | 0.56 | 1.47 | 0.000 | 0.279 | 1.000 | 4938 | tags=67%, list=22%, signal=85% |
| 189 | PROTEIN\_TARGETING\_TO\_ER(GO:0045047) |  | 15 | 0.56 | 1.47 | 0.000 | 0.277 | 1.000 | 4938 | tags=67%, list=22%, signal=85% |
| 190 | ESTABLISHMENT\_OF\_PROTEIN\_LOCALIZATION\_TO\_ENDOPLASMIC\_RETICULUM(GO:0072599) |  | 15 | 0.56 | 1.47 | 0.000 | 0.276 | 1.000 | 4938 | tags=67%, list=22%, signal=85% |
| 191 | GLYCOPROTEIN\_METABOLIC\_PROCESS(GO:0009100) |  | 57 | 0.39 | 1.47 | 0.000 | 0.276 | 1.000 | 7825 | tags=49%, list=35%, signal=75% |
| 192 | ESTABLISHMENT\_OF\_LOCALIZATION(GO:0051234) |  | 836 | 0.30 | 1.46 | 0.000 | 0.278 | 1.000 | 9161 | tags=46%, list=41%, signal=74% |
| 193 | ORGANELLE\_INNER\_MEMBRANE(GO:0019866) |  | 46 | 0.40 | 1.46 | 0.000 | 0.278 | 1.000 | 9572 | tags=70%, list=43%, signal=121% |
| 194 | ZINC\_ION\_BINDING(GO:0008270) |  | 543 | 0.27 | 1.46 | 0.000 | 0.288 | 1.000 | 8699 | tags=44%, list=39%, signal=71% |
| 195 | TRANSPORT(GO:0006810) |  | 831 | 0.30 | 1.46 | 0.000 | 0.287 | 1.000 | 9161 | tags=45%, list=41%, signal=74% |
| 196 | LOCALIZATION(GO:0051179) |  | 853 | 0.30 | 1.46 | 0.000 | 0.288 | 1.000 | 9161 | tags=45%, list=41%, signal=74% |
| 197 | LIPID\_BIOSYNTHETIC\_PROCESS(GO:0008610) |  | 52 | 0.34 | 1.46 | 0.000 | 0.288 | 1.000 | 11286 | tags=67%, list=50%, signal=135% |
| 198 | PRERIBOSOME(GO:0030684) |  | 22 | 0.42 | 1.45 | 0.000 | 0.289 | 1.000 | 6013 | tags=50%, list=27%, signal=68% |
| 199 | PROTEIN\_LOCALIZATION\_TO\_MEMBRANE(GO:0072657) |  | 20 | 0.46 | 1.45 | 0.000 | 0.288 | 1.000 | 7744 | tags=70%, list=35%, signal=107% |
| 200 | PHOSPHOLIPID\_METABOLIC\_PROCESS(GO:0006644) |  | 45 | 0.29 | 1.45 | 0.000 | 0.288 | 1.000 | 10795 | tags=60%, list=48%, signal=116% |
| 201 | ATP\_SYNTHESIS\_COUPLED\_PROTON\_TRANSPORT(GO:0015986) |  | 26 | 0.43 | 1.45 | 0.000 | 0.288 | 1.000 | 8663 | tags=65%, list=39%, signal=106% |
| 202 | ENERGY\_COUPLED\_PROTON\_TRANSPORT\_\_DOWN\_ELECTROCHEMICAL\_GRADIENT(GO:0015985) |  | 26 | 0.43 | 1.45 | 0.000 | 0.286 | 1.000 | 8663 | tags=65%, list=39%, signal=106% |
| 203 | MITOCHONDRIAL\_INNER\_MEMBRANE(GO:0005743) |  | 44 | 0.41 | 1.45 | 0.000 | 0.286 | 1.000 | 9572 | tags=70%, list=43%, signal=123% |
| 204 | GLYCOPROTEIN\_BIOSYNTHETIC\_PROCESS(GO:0009101) |  | 56 | 0.38 | 1.45 | 0.000 | 0.287 | 1.000 | 7825 | tags=48%, list=35%, signal=74% |
| 205 | MITOCHONDRIAL\_OUTER\_MEMBRANE(GO:0005741) |  | 25 | 0.44 | 1.45 | 0.000 | 0.288 | 1.000 | 8565 | tags=60%, list=38%, signal=97% |
| 206 | OUTER\_MEMBRANE(GO:0019867) |  | 25 | 0.44 | 1.45 | 0.000 | 0.286 | 1.000 | 8565 | tags=60%, list=38%, signal=97% |
| 207 | ORGANELLE\_OUTER\_MEMBRANE(GO:0031968) |  | 25 | 0.44 | 1.45 | 0.000 | 0.285 | 1.000 | 8565 | tags=60%, list=38%, signal=97% |
| 208 | ESTABLISHMENT\_OF\_PROTEIN\_LOCALIZATION\_TO\_MEMBRANE(GO:0090150) |  | 19 | 0.49 | 1.45 | 0.000 | 0.287 | 1.000 | 7744 | tags=74%, list=35%, signal=113% |
| 209 | CELLULAR\_MACROMOLECULE\_METABOLIC\_PROCESS(GO:0044260) |  | 2506 | 0.28 | 1.45 | 0.000 | 0.287 | 1.000 | 11225 | tags=57%, list=50%, signal=101% |
| 210 | POSITIVE\_REGULATION\_OF\_CELLULAR\_PROCESS(GO:0048522) |  | 45 | 0.32 | 1.44 | 0.000 | 0.287 | 1.000 | 10864 | tags=64%, list=48%, signal=125% |
| 211 | MACROMOLECULE\_METABOLIC\_PROCESS(GO:0043170) |  | 2919 | 0.28 | 1.44 | 0.000 | 0.286 | 1.000 | 10966 | tags=55%, list=49%, signal=93% |
| 212 | UBIQUITIN\_DEPENDENT\_PROTEIN\_CATABOLIC\_PROCESS(GO:0006511) |  | 37 | 0.47 | 1.44 | 0.000 | 0.287 | 1.000 | 10943 | tags=92%, list=49%, signal=179% |
| 213 | MODIFICATION\_DEPENDENT\_PROTEIN\_CATABOLIC\_PROCESS(GO:0019941) |  | 37 | 0.47 | 1.44 | 0.000 | 0.286 | 1.000 | 10943 | tags=92%, list=49%, signal=179% |
| 214 | MODIFICATION\_DEPENDENT\_MACROMOLECULE\_CATABOLIC\_PROCESS(GO:0043632) |  | 37 | 0.47 | 1.44 | 0.000 | 0.285 | 1.000 | 10943 | tags=92%, list=49%, signal=179% |
| 215 | TRANSCRIPTION\_REGULATOR\_ACTIVITY(GO:0140110) |  | 351 | 0.28 | 1.44 | 0.000 | 0.288 | 1.000 | 12086 | tags=61%, list=54%, signal=131% |
| 216 | UDP\_GLYCOSYLTRANSFERASE\_ACTIVITY(GO:0008194) |  | 32 | 0.34 | 1.44 | 0.000 | 0.287 | 1.000 | 4643 | tags=34%, list=21%, signal=43% |
| 217 | AMIDE\_BIOSYNTHETIC\_PROCESS(GO:0043604) |  | 609 | 0.39 | 1.44 | 0.175 | 0.287 | 1.000 | 9441 | tags=58%, list=42%, signal=97% |
| 218 | DNA\_BINDING(GO:0003677) |  | 786 | 0.28 | 1.44 | 0.000 | 0.289 | 1.000 | 11829 | tags=61%, list=53%, signal=125% |
| 219 | NITROGEN\_COMPOUND\_METABOLIC\_PROCESS(GO:0006807) |  | 3159 | 0.28 | 1.44 | 0.000 | 0.288 | 1.000 | 11180 | tags=56%, list=50%, signal=95% |
| 220 | MONOVALENT\_INORGANIC\_CATION\_TRANSPORT(GO:0015672) |  | 98 | 0.38 | 1.43 | 0.000 | 0.288 | 1.000 | 7913 | tags=49%, list=35%, signal=75% |
| 221 | CATION\_TRANSMEMBRANE\_TRANSPORTER\_ACTIVITY(GO:0008324) |  | 212 | 0.35 | 1.43 | 0.000 | 0.287 | 1.000 | 9132 | tags=51%, list=41%, signal=85% |
| 222 | CHROMOSOME(GO:0005694) |  | 78 | 0.32 | 1.43 | 0.000 | 0.287 | 1.000 | 13389 | tags=79%, list=60%, signal=197% |
| 223 | ATP\_HYDROLYSIS\_COUPLED\_PROTON\_TRANSPORT(GO:0015991) |  | 26 | 0.39 | 1.43 | 0.000 | 0.289 | 1.000 | 8405 | tags=58%, list=38%, signal=92% |
| 224 | ENERGY\_COUPLED\_PROTON\_TRANSMEMBRANE\_TRANSPORT\_\_AGAINST\_ELECTROCHEMICAL\_GRADIENT(GO:0015988) |  | 26 | 0.39 | 1.43 | 0.000 | 0.288 | 1.000 | 8405 | tags=58%, list=38%, signal=92% |
| 225 | ATP\_HYDROLYSIS\_COUPLED\_TRANSMEMBRANE\_TRANSPORT(GO:0090662) |  | 26 | 0.39 | 1.43 | 0.000 | 0.286 | 1.000 | 8405 | tags=58%, list=38%, signal=92% |
| 226 | ATP\_HYDROLYSIS\_COUPLED\_ION\_TRANSMEMBRANE\_TRANSPORT(GO:0099131) |  | 26 | 0.39 | 1.43 | 0.000 | 0.285 | 1.000 | 8405 | tags=58%, list=38%, signal=92% |
| 227 | ATP\_HYDROLYSIS\_COUPLED\_CATION\_TRANSMEMBRANE\_TRANSPORT(GO:0099132) |  | 26 | 0.39 | 1.43 | 0.000 | 0.284 | 1.000 | 8405 | tags=58%, list=38%, signal=92% |
| 228 | CARBOXYLIC\_ACID\_BINDING(GO:0031406) |  | 19 | 0.39 | 1.42 | 0.000 | 0.289 | 1.000 | 7294 | tags=47%, list=33%, signal=70% |
| 229 | ORGANIC\_ACID\_BINDING(GO:0043177) |  | 19 | 0.39 | 1.42 | 0.000 | 0.288 | 1.000 | 7294 | tags=47%, list=33%, signal=70% |
| 230 | OXIDOREDUCTASE\_ACTIVITY\_\_ACTING\_ON\_NAD(P)H(GO:0016651) |  | 23 | 0.36 | 1.42 | 0.000 | 0.287 | 1.000 | 10141 | tags=65%, list=45%, signal=119% |
| 231 | PHOSPHOPROTEIN\_PHOSPHATASE\_ACTIVITY(GO:0004721) |  | 84 | 0.33 | 1.42 | 0.000 | 0.287 | 1.000 | 8358 | tags=48%, list=37%, signal=76% |
| 232 | REGULATION\_OF\_CELLULAR\_COMPONENT\_ORGANIZATION(GO:0051128) |  | 58 | 0.34 | 1.42 | 0.000 | 0.286 | 1.000 | 11721 | tags=66%, list=52%, signal=137% |
| 233 | PEPTIDE\_BIOSYNTHETIC\_PROCESS(GO:0043043) |  | 602 | 0.39 | 1.42 | 0.175 | 0.286 | 1.000 | 9441 | tags=57%, list=42%, signal=97% |
| 234 | MACROMOLECULE\_MODIFICATION(GO:0043412) |  | 832 | 0.28 | 1.42 | 0.000 | 0.285 | 1.000 | 11692 | tags=61%, list=52%, signal=123% |
| 235 | RNA\_POLYMERASE\_II\_TRANSCRIPTION\_COFACTOR\_ACTIVITY(GO:0001104) |  | 20 | 0.35 | 1.42 | 0.000 | 0.287 | 1.000 | 11890 | tags=80%, list=53%, signal=170% |
| 236 | TRANSCRIPTION\_FACTOR\_ACTIVITY\_\_RNA\_POLYMERASE\_II\_TRANSCRIPTION\_FACTOR\_BINDING(GO:0001076) |  | 20 | 0.35 | 1.42 | 0.000 | 0.286 | 1.000 | 11890 | tags=80%, list=53%, signal=170% |
| 237 | CELLULAR\_AMIDE\_METABOLIC\_PROCESS(GO:0043603) |  | 628 | 0.39 | 1.42 | 0.175 | 0.286 | 1.000 | 9441 | tags=57%, list=42%, signal=96% |
| 238 | NUCLEOTIDYLTRANSFERASE\_ACTIVITY(GO:0016779) |  | 73 | 0.31 | 1.42 | 0.000 | 0.285 | 1.000 | 11674 | tags=71%, list=52%, signal=148% |
| 239 | REGULATION\_OF\_PROTEIN\_POLYMERIZATION(GO:0032271) |  | 24 | 0.41 | 1.42 | 0.000 | 0.288 | 1.000 | 10722 | tags=79%, list=48%, signal=152% |
| 240 | REGULATION\_OF\_PROTEIN\_COMPLEX\_ASSEMBLY(GO:0043254) |  | 24 | 0.41 | 1.42 | 0.000 | 0.286 | 1.000 | 10722 | tags=79%, list=48%, signal=152% |
| 241 | REGULATION\_OF\_CELLULAR\_COMPONENT\_BIOGENESIS(GO:0044087) |  | 24 | 0.41 | 1.42 | 0.000 | 0.285 | 1.000 | 10722 | tags=79%, list=48%, signal=152% |
| 242 | PROTEIN\_POLYMERIZATION(GO:0051258) |  | 24 | 0.41 | 1.42 | 0.000 | 0.284 | 1.000 | 10722 | tags=79%, list=48%, signal=152% |
| 243 | REGULATION\_OF\_SUPRAMOLECULAR\_FIBER\_ORGANIZATION(GO:1902903) |  | 24 | 0.41 | 1.42 | 0.000 | 0.283 | 1.000 | 10722 | tags=79%, list=48%, signal=152% |
| 244 | TRANSLATION(GO:0006412) |  | 600 | 0.39 | 1.42 | 0.175 | 0.282 | 1.000 | 9441 | tags=58%, list=42%, signal=97% |
| 245 | PRIMARY\_METABOLIC\_PROCESS(GO:0044238) |  | 3350 | 0.27 | 1.41 | 0.000 | 0.282 | 1.000 | 11180 | tags=55%, list=50%, signal=93% |
| 246 | PROTEIN\_GLYCOSYLATION(GO:0006486) |  | 51 | 0.36 | 1.41 | 0.000 | 0.290 | 1.000 | 7768 | tags=45%, list=35%, signal=69% |
| 247 | MACROMOLECULE\_GLYCOSYLATION(GO:0043413) |  | 51 | 0.36 | 1.41 | 0.000 | 0.289 | 1.000 | 7768 | tags=45%, list=35%, signal=69% |
| 248 | GLYCOSYLATION(GO:0070085) |  | 51 | 0.36 | 1.41 | 0.000 | 0.287 | 1.000 | 7768 | tags=45%, list=35%, signal=69% |
| 249 | CELLULAR\_PROTEIN\_MODIFICATION\_PROCESS(GO:0006464) |  | 806 | 0.28 | 1.41 | 0.000 | 0.288 | 1.000 | 11692 | tags=61%, list=52%, signal=124% |
| 250 | PROTEIN\_MODIFICATION\_PROCESS(GO:0036211) |  | 806 | 0.28 | 1.41 | 0.000 | 0.286 | 1.000 | 11692 | tags=61%, list=52%, signal=124% |
| 251 | ORGANIC\_SUBSTANCE\_METABOLIC\_PROCESS(GO:0071704) |  | 3428 | 0.27 | 1.41 | 0.000 | 0.286 | 1.000 | 11180 | tags=55%, list=50%, signal=93% |
| 252 | CELLULAR\_PROCESS(GO:0009987) |  | 4434 | 0.28 | 1.41 | 0.000 | 0.285 | 1.000 | 11232 | tags=55%, list=50%, signal=88% |
| 253 | ATP\_BINDING(GO:0005524) |  | 834 | 0.28 | 1.41 | 0.000 | 0.285 | 1.000 | 11466 | tags=61%, list=51%, signal=119% |
| 254 | ADENYL\_NUCLEOTIDE\_BINDING(GO:0030554) |  | 838 | 0.28 | 1.41 | 0.000 | 0.285 | 1.000 | 11466 | tags=61%, list=51%, signal=119% |
| 255 | ADENYL\_RIBONUCLEOTIDE\_BINDING(GO:0032559) |  | 835 | 0.28 | 1.41 | 0.000 | 0.285 | 1.000 | 11466 | tags=60%, list=51%, signal=119% |
| 256 | METHYLATION(GO:0032259) |  | 28 | 0.35 | 1.41 | 0.000 | 0.285 | 1.000 | 9058 | tags=61%, list=40%, signal=102% |
| 257 | INORGANIC\_CATION\_TRANSMEMBRANE\_TRANSPORTER\_ACTIVITY(GO:0022890) |  | 197 | 0.34 | 1.41 | 0.000 | 0.284 | 1.000 | 9132 | tags=50%, list=41%, signal=83% |
| 258 | CELLULAR\_PROTEIN\_CONTAINING\_COMPLEX\_ASSEMBLY(GO:0034622) |  | 83 | 0.34 | 1.41 | 0.000 | 0.284 | 1.000 | 9757 | tags=57%, list=44%, signal=100% |
| 259 | PURINE\_RIBONUCLEOSIDE\_TRIPHOSPHATE\_BINDING(GO:0035639) |  | 1093 | 0.28 | 1.41 | 0.000 | 0.284 | 1.000 | 10928 | tags=58%, list=49%, signal=108% |
| 260 | PURINE\_RIBONUCLEOTIDE\_BINDING(GO:0032555) |  | 1095 | 0.28 | 1.41 | 0.000 | 0.284 | 1.000 | 10928 | tags=58%, list=49%, signal=108% |
| 261 | MEDIATOR\_COMPLEX(GO:0016592) |  | 21 | 0.35 | 1.41 | 0.000 | 0.283 | 1.000 | 11948 | tags=81%, list=53%, signal=173% |
| 262 | PROTEIN\_CONTAINING\_COMPLEX(GO:0032991) |  | 1042 | 0.34 | 1.41 | 0.000 | 0.283 | 1.000 | 9765 | tags=55%, list=44%, signal=93% |
| 263 | PURINE\_NUCLEOTIDE\_BINDING(GO:0017076) |  | 1116 | 0.28 | 1.41 | 0.000 | 0.283 | 1.000 | 10857 | tags=58%, list=48%, signal=106% |
| 264 | ANION\_BINDING(GO:0043168) |  | 1288 | 0.28 | 1.41 | 0.000 | 0.282 | 1.000 | 10804 | tags=57%, list=48%, signal=103% |
| 265 | HETEROCYCLIC\_COMPOUND\_BINDING(GO:1901363) |  | 3075 | 0.27 | 1.40 | 0.000 | 0.283 | 1.000 | 10797 | tags=54%, list=48%, signal=90% |
| 266 | RIBONUCLEOPROTEIN\_COMPLEX\_BIOGENESIS(GO:0022613) |  | 48 | 0.30 | 1.40 | 0.175 | 0.282 | 1.000 | 12143 | tags=71%, list=54%, signal=154% |
| 267 | ORGANIC\_CYCLIC\_COMPOUND\_BINDING(GO:0097159) |  | 3076 | 0.27 | 1.40 | 0.000 | 0.282 | 1.000 | 10797 | tags=54%, list=48%, signal=90% |
| 268 | PROTON\_TRANSPORTING\_ATP\_SYNTHASE\_COMPLEX(GO:0045259) |  | 17 | 0.50 | 1.40 | 0.212 | 0.281 | 1.000 | 8663 | tags=76%, list=39%, signal=125% |
| 269 | CARBOHYDRATE\_DERIVATIVE\_BINDING(GO:0097367) |  | 1136 | 0.28 | 1.40 | 0.000 | 0.281 | 1.000 | 10804 | tags=57%, list=48%, signal=105% |
| 270 | REGULATION\_OF\_CELLULAR\_PROTEIN\_METABOLIC\_PROCESS(GO:0032268) |  | 20 | 0.42 | 1.40 | 0.000 | 0.281 | 1.000 | 6454 | tags=45%, list=29%, signal=63% |
| 271 | RIBONUCLEOTIDE\_BINDING(GO:0032553) |  | 1105 | 0.28 | 1.40 | 0.000 | 0.281 | 1.000 | 10928 | tags=58%, list=49%, signal=107% |
| 272 | MAGNESIUM\_ION\_BINDING(GO:0000287) |  | 38 | 0.31 | 1.40 | 0.000 | 0.281 | 1.000 | 5079 | tags=34%, list=23%, signal=44% |
| 273 | CATION\_TRANSMEMBRANE\_TRANSPORT(GO:0098655) |  | 72 | 0.36 | 1.40 | 0.000 | 0.281 | 1.000 | 7913 | tags=50%, list=35%, signal=77% |
| 274 | PURINE\_CONTAINING\_COMPOUND\_METABOLIC\_PROCESS(GO:0072521) |  | 95 | 0.34 | 1.40 | 0.000 | 0.281 | 1.000 | 8028 | tags=51%, list=36%, signal=78% |
| 275 | PEPTIDE\_METABOLIC\_PROCESS(GO:0006518) |  | 617 | 0.38 | 1.40 | 0.175 | 0.282 | 1.000 | 9441 | tags=57%, list=42%, signal=96% |
| 276 | PROTEOLYSIS(GO:0006508) |  | 369 | 0.30 | 1.39 | 0.000 | 0.282 | 1.000 | 10375 | tags=53%, list=46%, signal=98% |
| 277 | POSITIVE\_REGULATION\_OF\_METABOLIC\_PROCESS(GO:0009893) |  | 20 | 0.39 | 1.39 | 0.000 | 0.282 | 1.000 | 9553 | tags=65%, list=43%, signal=113% |
| 278 | POSITIVE\_REGULATION\_OF\_CELLULAR\_METABOLIC\_PROCESS(GO:0031325) |  | 20 | 0.39 | 1.39 | 0.000 | 0.281 | 1.000 | 9553 | tags=65%, list=43%, signal=113% |
| 279 | POSITIVE\_REGULATION\_OF\_MACROMOLECULE\_METABOLIC\_PROCESS(GO:0010604) |  | 18 | 0.41 | 1.39 | 0.000 | 0.281 | 1.000 | 9553 | tags=67%, list=43%, signal=116% |
| 280 | POSITIVE\_REGULATION\_OF\_NITROGEN\_COMPOUND\_METABOLIC\_PROCESS(GO:0051173) |  | 18 | 0.41 | 1.39 | 0.000 | 0.280 | 1.000 | 9553 | tags=67%, list=43%, signal=116% |
| 281 | DRUG\_BINDING(GO:0008144) |  | 879 | 0.27 | 1.39 | 0.000 | 0.281 | 1.000 | 11422 | tags=60%, list=51%, signal=117% |
| 282 | KINASE\_REGULATOR\_ACTIVITY(GO:0019207) |  | 20 | 0.56 | 1.39 | 0.000 | 0.285 | 1.000 | 3971 | tags=50%, list=18%, signal=61% |
| 283 | TRANSMEMBRANE\_TRANSPORT(GO:0055085) |  | 370 | 0.31 | 1.38 | 0.000 | 0.284 | 1.000 | 8853 | tags=42%, list=40%, signal=69% |
| 284 | GENE\_EXPRESSION(GO:0010467) |  | 1639 | 0.28 | 1.38 | 0.000 | 0.284 | 1.000 | 10724 | tags=54%, list=48%, signal=96% |
| 285 | REGULATION\_OF\_ORGANELLE\_ORGANIZATION(GO:0033043) |  | 36 | 0.33 | 1.38 | 0.000 | 0.283 | 1.000 | 11721 | tags=72%, list=52%, signal=151% |
| 286 | PHOSPHATIDYLINOSITOL\_METABOLIC\_PROCESS(GO:0046488) |  | 31 | 0.35 | 1.38 | 0.000 | 0.283 | 1.000 | 9542 | tags=65%, list=43%, signal=112% |
| 287 | NAD\_BINDING(GO:0051287) |  | 23 | 0.41 | 1.38 | 0.000 | 0.282 | 1.000 | 8389 | tags=57%, list=37%, signal=90% |
| 288 | ISOMERASE\_ACTIVITY(GO:0016853) |  | 97 | 0.33 | 1.38 | 0.000 | 0.282 | 1.000 | 8304 | tags=52%, list=37%, signal=82% |
| 289 | CELLULAR\_NITROGEN\_COMPOUND\_METABOLIC\_PROCESS(GO:0034641) |  | 1992 | 0.27 | 1.38 | 0.000 | 0.284 | 1.000 | 11180 | tags=55%, list=50%, signal=101% |
| 290 | PURINE\_NUCLEOTIDE\_METABOLIC\_PROCESS(GO:0006163) |  | 94 | 0.33 | 1.38 | 0.000 | 0.284 | 1.000 | 8028 | tags=50%, list=36%, signal=78% |
| 291 | ATPASE\_ACTIVITY\_\_COUPLED(GO:0042623) |  | 65 | 0.28 | 1.38 | 0.000 | 0.284 | 1.000 | 12210 | tags=68%, list=55%, signal=148% |
| 292 | METABOLIC\_PROCESS(GO:0008152) |  | 4050 | 0.26 | 1.38 | 0.000 | 0.285 | 1.000 | 11180 | tags=54%, list=50%, signal=88% |
| 293 | METAL\_ION\_TRANSPORT(GO:0030001) |  | 85 | 0.33 | 1.37 | 0.000 | 0.286 | 1.000 | 8393 | tags=45%, list=37%, signal=71% |
| 294 | ANION\_TRANSPORT(GO:0006820) |  | 52 | 0.34 | 1.37 | 0.000 | 0.289 | 1.000 | 12066 | tags=69%, list=54%, signal=150% |
| 295 | PEPTIDASE\_COMPLEX(GO:1905368) |  | 37 | 0.41 | 1.37 | 0.193 | 0.290 | 1.000 | 11026 | tags=78%, list=49%, signal=154% |
| 296 | PROTEIN\_DEPHOSPHORYLATION(GO:0006470) |  | 81 | 0.31 | 1.37 | 0.000 | 0.290 | 1.000 | 8079 | tags=44%, list=36%, signal=69% |
| 297 | BIOSYNTHETIC\_PROCESS(GO:0009058) |  | 1863 | 0.28 | 1.37 | 0.000 | 0.289 | 1.000 | 8912 | tags=45%, list=40%, signal=68% |
| 298 | PROTEIN\_CONTAINING\_COMPLEX\_BINDING(GO:0044877) |  | 74 | 0.31 | 1.36 | 0.000 | 0.290 | 1.000 | 12410 | tags=69%, list=55%, signal=154% |
| 299 | CYTOPLASM(GO:0005737) |  | 974 | 0.33 | 1.36 | 0.175 | 0.291 | 1.000 | 9180 | tags=51%, list=41%, signal=83% |
| 300 | DEPHOSPHORYLATION(GO:0016311) |  | 83 | 0.31 | 1.36 | 0.000 | 0.290 | 1.000 | 8079 | tags=45%, list=36%, signal=69% |
| 301 | ORGANIC\_SUBSTANCE\_BIOSYNTHETIC\_PROCESS(GO:1901576) |  | 1820 | 0.28 | 1.36 | 0.000 | 0.290 | 1.000 | 8912 | tags=45%, list=40%, signal=68% |
| 302 | GLYCEROPHOSPHOLIPID\_METABOLIC\_PROCESS(GO:0006650) |  | 33 | 0.34 | 1.36 | 0.000 | 0.290 | 1.000 | 11286 | tags=73%, list=50%, signal=146% |
| 303 | GLYCEROLIPID\_METABOLIC\_PROCESS(GO:0046486) |  | 33 | 0.34 | 1.36 | 0.000 | 0.289 | 1.000 | 11286 | tags=73%, list=50%, signal=146% |
| 304 | CHROMOSOMAL\_PART(GO:0044427) |  | 63 | 0.30 | 1.36 | 0.000 | 0.295 | 1.000 | 13389 | tags=75%, list=60%, signal=185% |
| 305 | SULFUR\_COMPOUND\_BINDING(GO:1901681) |  | 23 | 0.33 | 1.36 | 0.000 | 0.296 | 1.000 | 10306 | tags=57%, list=46%, signal=105% |
| 306 | EXTRACELLULAR\_MATRIX(GO:0031012) |  | 58 | 0.40 | 1.36 | 0.174 | 0.301 | 1.000 | 2144 | tags=21%, list=10%, signal=23% |
| 307 | INTRACELLULAR(GO:0005622) |  | 2238 | 0.27 | 1.36 | 0.000 | 0.301 | 1.000 | 11200 | tags=55%, list=50%, signal=99% |
| 308 | CATALYTIC\_ACTIVITY\_\_ACTING\_ON\_A\_PROTEIN(GO:0140096) |  | 1067 | 0.27 | 1.36 | 0.000 | 0.301 | 1.000 | 11154 | tags=56%, list=50%, signal=107% |
| 309 | ACTIVE\_TRANSMEMBRANE\_TRANSPORTER\_ACTIVITY(GO:0022804) |  | 80 | 0.30 | 1.35 | 0.000 | 0.301 | 1.000 | 8663 | tags=45%, list=39%, signal=73% |
| 310 | TRNA\_PROCESSING(GO:0008033) |  | 35 | 0.35 | 1.35 | 0.000 | 0.303 | 1.000 | 12681 | tags=86%, list=57%, signal=197% |
| 311 | REGULATION\_OF\_TRANSCRIPTION\_BY\_RNA\_POLYMERASE\_II(GO:0006357) |  | 24 | 0.35 | 1.35 | 0.000 | 0.303 | 1.000 | 11890 | tags=79%, list=53%, signal=169% |
| 312 | ANION\_TRANSMEMBRANE\_TRANSPORTER\_ACTIVITY(GO:0008509) |  | 47 | 0.38 | 1.35 | 0.000 | 0.303 | 1.000 | 11602 | tags=70%, list=52%, signal=145% |
| 313 | WNT\_SIGNALING\_PATHWAY(GO:0016055) |  | 27 | 0.54 | 1.35 | 0.000 | 0.306 | 1.000 | 2889 | tags=26%, list=13%, signal=30% |
| 314 | CELL\_CELL\_SIGNALING\_BY\_WNT(GO:0198738) |  | 27 | 0.54 | 1.35 | 0.000 | 0.305 | 1.000 | 2889 | tags=26%, list=13%, signal=30% |
| 315 | CELL\_SURFACE\_RECEPTOR\_SIGNALING\_PATHWAY\_INVOLVED\_IN\_CELL\_CELL\_SIGNALING(GO:1905114) |  | 27 | 0.54 | 1.35 | 0.000 | 0.304 | 1.000 | 2889 | tags=26%, list=13%, signal=30% |
| 316 | CELLULAR\_BIOSYNTHETIC\_PROCESS(GO:0044249) |  | 1810 | 0.28 | 1.35 | 0.000 | 0.304 | 1.000 | 8912 | tags=45%, list=40%, signal=68% |
| 317 | REGULATION\_OF\_PROTEIN\_METABOLIC\_PROCESS(GO:0051246) |  | 21 | 0.39 | 1.35 | 0.000 | 0.309 | 1.000 | 6454 | tags=43%, list=29%, signal=60% |
| 318 | CHROMATIN(GO:0000785) |  | 35 | 0.44 | 1.35 | 0.000 | 0.309 | 1.000 | 3319 | tags=29%, list=15%, signal=33% |
| 319 | DNA\_BINDING\_TRANSCRIPTION\_FACTOR\_ACTIVITY(GO:0003700) |  | 319 | 0.27 | 1.34 | 0.000 | 0.312 | 1.000 | 11332 | tags=55%, list=51%, signal=109% |
| 320 | CYTOPLASMIC\_VESICLE(GO:0031410) |  | 26 | 0.35 | 1.34 | 0.000 | 0.312 | 1.000 | 10225 | tags=69%, list=46%, signal=127% |
| 321 | VESICLE(GO:0031982) |  | 26 | 0.35 | 1.34 | 0.000 | 0.311 | 1.000 | 10225 | tags=69%, list=46%, signal=127% |
| 322 | INTRACELLULAR\_VESICLE(GO:0097708) |  | 26 | 0.35 | 1.34 | 0.000 | 0.310 | 1.000 | 10225 | tags=69%, list=46%, signal=127% |
| 323 | CELL(GO:0005623) |  | 2296 | 0.27 | 1.34 | 0.000 | 0.314 | 1.000 | 11130 | tags=55%, list=50%, signal=97% |
| 324 | CELL\_PART(GO:0044464) |  | 2296 | 0.27 | 1.34 | 0.000 | 0.313 | 1.000 | 11130 | tags=55%, list=50%, signal=97% |
| 325 | TRANSITION\_METAL\_ION\_BINDING(GO:0046914) |  | 647 | 0.25 | 1.34 | 0.000 | 0.313 | 1.000 | 12341 | tags=60%, list=55%, signal=129% |
| 326 | PROTEIN\_LOCALIZATION\_TO\_ORGANELLE(GO:0033365) |  | 33 | 0.49 | 1.34 | 0.000 | 0.314 | 1.000 | 10052 | tags=85%, list=45%, signal=154% |
| 327 | CYTOSKELETON\_ORGANIZATION(GO:0007010) |  | 85 | 0.29 | 1.34 | 0.000 | 0.313 | 1.000 | 12781 | tags=72%, list=57%, signal=166% |
| 328 | VOLTAGE\_GATED\_POTASSIUM\_CHANNEL\_ACTIVITY(GO:0005249) |  | 16 | 0.52 | 1.34 | 0.000 | 0.313 | 1.000 | 3326 | tags=38%, list=15%, signal=44% |
| 329 | SMALL\_MOLECULE\_BIOSYNTHETIC\_PROCESS(GO:0044283) |  | 82 | 0.33 | 1.33 | 0.000 | 0.316 | 1.000 | 8028 | tags=44%, list=36%, signal=68% |
| 330 | SUPRAMOLECULAR\_FIBER\_ORGANIZATION(GO:0097435) |  | 36 | 0.40 | 1.33 | 0.000 | 0.317 | 1.000 | 10815 | tags=75%, list=48%, signal=145% |
| 331 | MONOVALENT\_INORGANIC\_CATION\_TRANSMEMBRANE\_TRANSPORTER\_ACTIVITY(GO:0015077) |  | 153 | 0.35 | 1.33 | 0.000 | 0.318 | 1.000 | 8430 | tags=48%, list=38%, signal=76% |
| 332 | GUANYL\_NUCLEOTIDE\_BINDING(GO:0019001) |  | 280 | 0.32 | 1.33 | 0.000 | 0.317 | 1.000 | 7908 | tags=48%, list=35%, signal=74% |
| 333 | MULTICELLULAR\_ORGANISMAL\_PROCESS(GO:0032501) |  | 98 | 0.37 | 1.33 | 0.000 | 0.317 | 1.000 | 6570 | tags=34%, list=29%, signal=47% |
| 334 | REGULATION\_OF\_ANATOMICAL\_STRUCTURE\_SIZE(GO:0090066) |  | 21 | 0.40 | 1.33 | 0.000 | 0.316 | 1.000 | 11633 | tags=81%, list=52%, signal=168% |
| 335 | NEGATIVE\_REGULATION\_OF\_MACROMOLECULE\_BIOSYNTHETIC\_PROCESS(GO:0010558) |  | 15 | 0.34 | 1.33 | 0.000 | 0.317 | 1.000 | 6852 | tags=47%, list=31%, signal=67% |
| 336 | NEGATIVE\_REGULATION\_OF\_CELLULAR\_BIOSYNTHETIC\_PROCESS(GO:0031327) |  | 15 | 0.34 | 1.33 | 0.000 | 0.316 | 1.000 | 6852 | tags=47%, list=31%, signal=67% |
| 337 | NEGATIVE\_REGULATION\_OF\_CELLULAR\_MACROMOLECULE\_BIOSYNTHETIC\_PROCESS(GO:2000113) |  | 15 | 0.34 | 1.33 | 0.000 | 0.315 | 1.000 | 6852 | tags=47%, list=31%, signal=67% |
| 338 | CYTOPLASMIC\_PART(GO:0044444) |  | 841 | 0.34 | 1.33 | 0.175 | 0.314 | 1.000 | 9180 | tags=52%, list=41%, signal=86% |
| 339 | RIBOSE\_PHOSPHATE\_METABOLIC\_PROCESS(GO:0019693) |  | 93 | 0.32 | 1.33 | 0.000 | 0.317 | 1.000 | 8028 | tags=49%, list=36%, signal=77% |
| 340 | ORGANIC\_ACID\_BIOSYNTHETIC\_PROCESS(GO:0016053) |  | 53 | 0.35 | 1.33 | 0.000 | 0.319 | 1.000 | 4110 | tags=26%, list=18%, signal=32% |
| 341 | CARBOXYLIC\_ACID\_BIOSYNTHETIC\_PROCESS(GO:0046394) |  | 53 | 0.35 | 1.33 | 0.000 | 0.318 | 1.000 | 4110 | tags=26%, list=18%, signal=32% |
| 342 | GTP\_BINDING(GO:0005525) |  | 261 | 0.33 | 1.33 | 0.000 | 0.319 | 1.000 | 7908 | tags=49%, list=35%, signal=74% |
| 343 | PURINE\_NUCLEOSIDE\_BINDING(GO:0001883) |  | 261 | 0.33 | 1.33 | 0.000 | 0.318 | 1.000 | 7908 | tags=49%, list=35%, signal=74% |
| 344 | RIBONUCLEOSIDE\_BINDING(GO:0032549) |  | 261 | 0.33 | 1.33 | 0.000 | 0.317 | 1.000 | 7908 | tags=49%, list=35%, signal=74% |
| 345 | PURINE\_RIBONUCLEOSIDE\_BINDING(GO:0032550) |  | 261 | 0.33 | 1.33 | 0.000 | 0.316 | 1.000 | 7908 | tags=49%, list=35%, signal=74% |
| 346 | GUANYL\_RIBONUCLEOTIDE\_BINDING(GO:0032561) |  | 262 | 0.33 | 1.33 | 0.000 | 0.316 | 1.000 | 7908 | tags=48%, list=35%, signal=74% |
| 347 | CARBOHYDRATE\_DERIVATIVE\_BIOSYNTHETIC\_PROCESS(GO:1901137) |  | 160 | 0.31 | 1.33 | 0.000 | 0.316 | 1.000 | 8180 | tags=47%, list=37%, signal=73% |
| 348 | REGULATION\_OF\_CATALYTIC\_ACTIVITY(GO:0050790) |  | 32 | 0.28 | 1.32 | 0.000 | 0.317 | 1.000 | 7894 | tags=44%, list=35%, signal=67% |
| 349 | NUCLEOSIDE\_BINDING(GO:0001882) |  | 262 | 0.33 | 1.32 | 0.000 | 0.320 | 1.000 | 7908 | tags=48%, list=35%, signal=74% |
| 350 | ION\_TRANSPORT(GO:0006811) |  | 351 | 0.31 | 1.32 | 0.000 | 0.322 | 1.000 | 8430 | tags=40%, list=38%, signal=62% |
| 351 | TRANSPORTER\_ACTIVITY(GO:0005215) |  | 504 | 0.31 | 1.32 | 0.000 | 0.323 | 1.000 | 9134 | tags=43%, list=41%, signal=71% |
| 352 | CARBOHYDRATE\_DERIVATIVE\_METABOLIC\_PROCESS(GO:1901135) |  | 206 | 0.31 | 1.32 | 0.000 | 0.322 | 1.000 | 8180 | tags=47%, list=37%, signal=73% |
| 353 | LIPID\_METABOLIC\_PROCESS(GO:0006629) |  | 141 | 0.27 | 1.32 | 0.000 | 0.322 | 1.000 | 7300 | tags=40%, list=33%, signal=60% |
| 354 | TRANSFERASE\_ACTIVITY(GO:0016740) |  | 1116 | 0.26 | 1.32 | 0.000 | 0.327 | 1.000 | 13165 | tags=67%, list=59%, signal=154% |
| 355 | SYMPORTER\_ACTIVITY(GO:0015293) |  | 22 | 0.45 | 1.32 | 0.000 | 0.327 | 1.000 | 7372 | tags=50%, list=33%, signal=74% |
| 356 | SOLUTE:CATION\_SYMPORTER\_ACTIVITY(GO:0015294) |  | 22 | 0.45 | 1.32 | 0.000 | 0.326 | 1.000 | 7372 | tags=50%, list=33%, signal=74% |
| 357 | GOLGI\_VESICLE\_TRANSPORT(GO:0048193) |  | 22 | 0.37 | 1.31 | 0.000 | 0.326 | 1.000 | 10292 | tags=73%, list=46%, signal=134% |
| 358 | DRUG\_METABOLIC\_PROCESS(GO:0017144) |  | 85 | 0.34 | 1.31 | 0.000 | 0.325 | 1.000 | 7953 | tags=48%, list=35%, signal=74% |
| 359 | PROTEIN\_HETERODIMERIZATION\_ACTIVITY(GO:0046982) |  | 114 | 0.36 | 1.31 | 0.160 | 0.325 | 1.000 | 3897 | tags=30%, list=17%, signal=36% |
| 360 | PROTEIN\_MODIFICATION\_BY\_SMALL\_PROTEIN\_CONJUGATION\_OR\_REMOVAL(GO:0070647) |  | 72 | 0.36 | 1.31 | 0.000 | 0.324 | 1.000 | 8644 | tags=60%, list=39%, signal=97% |
| 361 | PEPTIDASE\_ACTIVITY(GO:0008233) |  | 378 | 0.29 | 1.31 | 0.000 | 0.324 | 1.000 | 10348 | tags=51%, list=46%, signal=93% |
| 362 | ACTIN\_CYTOSKELETON\_ORGANIZATION(GO:0030036) |  | 42 | 0.36 | 1.31 | 0.000 | 0.324 | 1.000 | 10815 | tags=71%, list=48%, signal=138% |
| 363 | ACTIN\_FILAMENT\_BASED\_PROCESS(GO:0030029) |  | 42 | 0.36 | 1.31 | 0.000 | 0.323 | 1.000 | 10815 | tags=71%, list=48%, signal=138% |
| 364 | RECEPTOR\_LIGAND\_ACTIVITY(GO:0048018) |  | 125 | 0.43 | 1.31 | 0.174 | 0.323 | 1.000 | 3926 | tags=25%, list=18%, signal=30% |
| 365 | RECEPTOR\_REGULATOR\_ACTIVITY(GO:0030545) |  | 125 | 0.43 | 1.31 | 0.174 | 0.322 | 1.000 | 3926 | tags=25%, list=18%, signal=30% |
| 366 | PEPTIDASE\_ACTIVITY\_\_ACTING\_ON\_L\_AMINO\_ACID\_PEPTIDES(GO:0070011) |  | 365 | 0.29 | 1.31 | 0.000 | 0.322 | 1.000 | 10348 | tags=51%, list=46%, signal=93% |
| 367 | INORGANIC\_MOLECULAR\_ENTITY\_TRANSMEMBRANE\_TRANSPORTER\_ACTIVITY(GO:0015318) |  | 344 | 0.33 | 1.31 | 0.000 | 0.321 | 1.000 | 5464 | tags=27%, list=24%, signal=36% |
| 368 | TRANSFERASE\_ACTIVITY\_\_TRANSFERRING\_GLYCOSYL\_GROUPS(GO:0016757) |  | 152 | 0.28 | 1.31 | 0.000 | 0.321 | 1.000 | 6729 | tags=34%, list=30%, signal=49% |
| 369 | METHYLTRANSFERASE\_ACTIVITY(GO:0008168) |  | 85 | 0.26 | 1.31 | 0.000 | 0.320 | 1.000 | 14426 | tags=80%, list=64%, signal=224% |
| 370 | MEMBRANE(GO:0016020) |  | 1719 | 0.29 | 1.31 | 0.000 | 0.320 | 1.000 | 7966 | tags=38%, list=36%, signal=54% |
| 371 | TRANSMEMBRANE\_TRANSPORTER\_ACTIVITY(GO:0022857) |  | 442 | 0.31 | 1.31 | 0.000 | 0.319 | 1.000 | 8430 | tags=40%, list=38%, signal=62% |
| 372 | MACROMOLECULE\_BIOSYNTHETIC\_PROCESS(GO:0009059) |  | 1614 | 0.28 | 1.31 | 0.000 | 0.320 | 1.000 | 11023 | tags=54%, list=49%, signal=99% |
| 373 | PURINE\_CONTAINING\_COMPOUND\_BIOSYNTHETIC\_PROCESS(GO:0072522) |  | 81 | 0.32 | 1.31 | 0.000 | 0.320 | 1.000 | 8028 | tags=48%, list=36%, signal=75% |
| 374 | PROTEIN\_CONTAINING\_COMPLEX\_DISASSEMBLY(GO:0032984) |  | 19 | 0.38 | 1.31 | 0.163 | 0.320 | 1.000 | 12684 | tags=79%, list=57%, signal=182% |
| 375 | CELLULAR\_PROTEIN\_COMPLEX\_DISASSEMBLY(GO:0043624) |  | 19 | 0.38 | 1.31 | 0.163 | 0.319 | 1.000 | 12684 | tags=79%, list=57%, signal=182% |
| 376 | CELLULAR\_MACROMOLECULE\_BIOSYNTHETIC\_PROCESS(GO:0034645) |  | 1611 | 0.28 | 1.31 | 0.000 | 0.319 | 1.000 | 11023 | tags=54%, list=49%, signal=99% |
| 377 | PURINE\_RIBONUCLEOTIDE\_METABOLIC\_PROCESS(GO:0009150) |  | 90 | 0.32 | 1.31 | 0.000 | 0.319 | 1.000 | 8028 | tags=49%, list=36%, signal=76% |
| 378 | RIBONUCLEOTIDE\_METABOLIC\_PROCESS(GO:0009259) |  | 90 | 0.32 | 1.31 | 0.000 | 0.318 | 1.000 | 8028 | tags=49%, list=36%, signal=76% |
| 379 | TRANSFERASE\_ACTIVITY\_\_TRANSFERRING\_PHOSPHORUS\_CONTAINING\_GROUPS(GO:0016772) |  | 674 | 0.27 | 1.31 | 0.000 | 0.318 | 1.000 | 11732 | tags=61%, list=52%, signal=124% |
| 380 | DNA\_DIRECTED\_5'\_3'\_RNA\_POLYMERASE\_ACTIVITY(GO:0003899) |  | 35 | 0.40 | 1.31 | 0.212 | 0.318 | 1.000 | 11674 | tags=86%, list=52%, signal=179% |
| 381 | 5'\_3'\_RNA\_POLYMERASE\_ACTIVITY(GO:0034062) |  | 35 | 0.40 | 1.31 | 0.212 | 0.317 | 1.000 | 11674 | tags=86%, list=52%, signal=179% |
| 382 | RNA\_POLYMERASE\_ACTIVITY(GO:0097747) |  | 35 | 0.40 | 1.31 | 0.212 | 0.317 | 1.000 | 11674 | tags=86%, list=52%, signal=179% |
| 383 | DNA\_METABOLIC\_PROCESS(GO:0006259) |  | 145 | 0.27 | 1.31 | 0.163 | 0.316 | 1.000 | 13893 | tags=74%, list=62%, signal=193% |
| 384 | NEGATIVE\_REGULATION\_OF\_MACROMOLECULE\_METABOLIC\_PROCESS(GO:0010605) |  | 27 | 0.29 | 1.30 | 0.175 | 0.317 | 1.000 | 10924 | tags=59%, list=49%, signal=116% |
| 385 | CELLULAR\_NITROGEN\_COMPOUND\_BIOSYNTHETIC\_PROCESS(GO:0044271) |  | 1642 | 0.28 | 1.30 | 0.000 | 0.318 | 1.000 | 8912 | tags=44%, list=40%, signal=68% |
| 386 | ION\_TRANSMEMBRANE\_TRANSPORTER\_ACTIVITY(GO:0015075) |  | 370 | 0.32 | 1.30 | 0.000 | 0.318 | 1.000 | 9134 | tags=44%, list=41%, signal=72% |
| 387 | TRANSLATIONAL\_ELONGATION(GO:0006414) |  | 16 | 0.50 | 1.30 | 0.000 | 0.318 | 1.000 | 5293 | tags=44%, list=24%, signal=57% |
| 388 | TRANSLATION\_ELONGATION\_FACTOR\_ACTIVITY(GO:0003746) |  | 16 | 0.50 | 1.30 | 0.000 | 0.317 | 1.000 | 5293 | tags=44%, list=24%, signal=57% |
| 389 | MITOCHONDRIAL\_RESPIRATORY\_CHAIN(GO:0005746) |  | 22 | 0.46 | 1.30 | 0.000 | 0.317 | 1.000 | 9572 | tags=73%, list=43%, signal=127% |
| 390 | RESPIRATORY\_CHAIN(GO:0070469) |  | 22 | 0.46 | 1.30 | 0.000 | 0.316 | 1.000 | 9572 | tags=73%, list=43%, signal=127% |
| 391 | REGULATION\_OF\_CYTOSKELETON\_ORGANIZATION(GO:0051493) |  | 31 | 0.34 | 1.30 | 0.000 | 0.316 | 1.000 | 10722 | tags=68%, list=48%, signal=130% |
| 392 | VACUOLAR\_TRANSPORT(GO:0007034) |  | 15 | 0.46 | 1.30 | 0.000 | 0.320 | 1.000 | 11493 | tags=87%, list=51%, signal=178% |
| 393 | RIBONUCLEOPROTEIN\_COMPLEX(GO:1990904) |  | 566 | 0.37 | 1.29 | 0.175 | 0.322 | 1.000 | 9210 | tags=54%, list=41%, signal=90% |
| 394 | STRUCTURAL\_MOLECULE\_ACTIVITY(GO:0005198) |  | 651 | 0.36 | 1.29 | 0.175 | 0.322 | 1.000 | 6539 | tags=40%, list=29%, signal=55% |
| 395 | CYTOSKELETAL\_PROTEIN\_BINDING(GO:0008092) |  | 148 | 0.25 | 1.29 | 0.000 | 0.323 | 1.000 | 14444 | tags=76%, list=64%, signal=213% |
| 396 | HISTONE\_MODIFICATION(GO:0016570) |  | 16 | 0.37 | 1.29 | 0.000 | 0.325 | 1.000 | 11860 | tags=81%, list=53%, signal=173% |
| 397 | COVALENT\_CHROMATIN\_MODIFICATION(GO:0016569) |  | 16 | 0.37 | 1.29 | 0.000 | 0.324 | 1.000 | 11860 | tags=81%, list=53%, signal=173% |
| 398 | REGULATION\_OF\_CELL\_CYCLE(GO:0051726) |  | 31 | 0.32 | 1.29 | 0.000 | 0.324 | 1.000 | 11102 | tags=65%, list=50%, signal=128% |
| 399 | CATALYTIC\_ACTIVITY(GO:0003824) |  | 3158 | 0.25 | 1.29 | 0.000 | 0.325 | 1.000 | 12549 | tags=60%, list=56%, signal=117% |
| 400 | VOLTAGE\_GATED\_CATION\_CHANNEL\_ACTIVITY(GO:0022843) |  | 19 | 0.48 | 1.28 | 0.000 | 0.325 | 1.000 | 3326 | tags=32%, list=15%, signal=37% |
| 401 | INNER\_MITOCHONDRIAL\_MEMBRANE\_PROTEIN\_COMPLEX(GO:0098800) |  | 28 | 0.46 | 1.28 | 0.175 | 0.324 | 1.000 | 9572 | tags=71%, list=43%, signal=125% |
| 402 | PURINE\_NUCLEOTIDE\_BIOSYNTHETIC\_PROCESS(GO:0006164) |  | 80 | 0.31 | 1.28 | 0.000 | 0.328 | 1.000 | 8028 | tags=48%, list=36%, signal=74% |
| 403 | PROTEIN\_KINASE\_ACTIVITY(GO:0004672) |  | 478 | 0.27 | 1.28 | 0.000 | 0.332 | 1.000 | 11732 | tags=60%, list=52%, signal=124% |
| 404 | CELLULAR\_CATABOLIC\_PROCESS(GO:0044248) |  | 116 | 0.28 | 1.28 | 0.000 | 0.336 | 1.000 | 12544 | tags=67%, list=56%, signal=152% |
| 405 | HYDROLASE\_ACTIVITY\_\_ACTING\_ON\_ESTER\_BONDS(GO:0016788) |  | 276 | 0.25 | 1.28 | 0.000 | 0.337 | 1.000 | 13027 | tags=64%, list=58%, signal=151% |
| 406 | PROTEIN\_DIMERIZATION\_ACTIVITY(GO:0046983) |  | 213 | 0.32 | 1.28 | 0.000 | 0.337 | 1.000 | 7743 | tags=44%, list=35%, signal=66% |
| 407 | TRANSFERASE\_ACTIVITY\_\_TRANSFERRING\_ONE\_CARBON\_GROUPS(GO:0016741) |  | 92 | 0.27 | 1.28 | 0.000 | 0.340 | 1.000 | 14426 | tags=80%, list=64%, signal=225% |
| 408 | CELLULAR\_COMPONENT(GO:0005575) |  | 4058 | 0.27 | 1.27 | 0.000 | 0.343 | 1.000 | 8058 | tags=38%, list=36%, signal=48% |
| 409 | PHOSPHORUS\_METABOLIC\_PROCESS(GO:0006793) |  | 750 | 0.26 | 1.27 | 0.000 | 0.346 | 1.000 | 11154 | tags=56%, list=50%, signal=107% |
| 410 | PHOSPHATE\_CONTAINING\_COMPOUND\_METABOLIC\_PROCESS(GO:0006796) |  | 750 | 0.26 | 1.27 | 0.000 | 0.346 | 1.000 | 11154 | tags=56%, list=50%, signal=107% |
| 411 | MEMBRANE\_PART(GO:0044425) |  | 1042 | 0.28 | 1.27 | 0.174 | 0.345 | 1.000 | 7941 | tags=39%, list=35%, signal=58% |
| 412 | DNA\_REPLICATION(GO:0006260) |  | 48 | 0.30 | 1.27 | 0.197 | 0.350 | 1.000 | 13330 | tags=81%, list=60%, signal=200% |
| 413 | STRUCTURAL\_CONSTITUENT\_OF\_RIBOSOME(GO:0003735) |  | 518 | 0.38 | 1.27 | 0.175 | 0.350 | 1.000 | 9441 | tags=56%, list=42%, signal=94% |
| 414 | NUCLEOTIDE\_METABOLIC\_PROCESS(GO:0009117) |  | 136 | 0.28 | 1.27 | 0.000 | 0.350 | 1.000 | 8028 | tags=45%, list=36%, signal=69% |
| 415 | CELLULAR\_COMPONENT\_DISASSEMBLY(GO:0022411) |  | 21 | 0.39 | 1.27 | 0.000 | 0.351 | 1.000 | 12684 | tags=81%, list=57%, signal=186% |
| 416 | POSITIVE\_REGULATION\_OF\_BIOLOGICAL\_PROCESS(GO:0048518) |  | 48 | 0.31 | 1.27 | 0.000 | 0.351 | 1.000 | 10864 | tags=60%, list=48%, signal=117% |
| 417 | HETEROCYCLE\_METABOLIC\_PROCESS(GO:0046483) |  | 1411 | 0.23 | 1.27 | 0.000 | 0.351 | 1.000 | 13017 | tags=63%, list=58%, signal=140% |
| 418 | ORGANIC\_CYCLIC\_COMPOUND\_METABOLIC\_PROCESS(GO:1901360) |  | 1422 | 0.23 | 1.27 | 0.000 | 0.351 | 1.000 | 13017 | tags=63%, list=58%, signal=140% |
| 419 | VOLTAGE\_GATED\_ION\_CHANNEL\_ACTIVITY(GO:0005244) |  | 28 | 0.37 | 1.27 | 0.000 | 0.351 | 1.000 | 2146 | tags=21%, list=10%, signal=24% |
| 420 | VOLTAGE\_GATED\_CHANNEL\_ACTIVITY(GO:0022832) |  | 28 | 0.37 | 1.27 | 0.000 | 0.350 | 1.000 | 2146 | tags=21%, list=10%, signal=24% |
| 421 | GLYCEROLIPID\_BIOSYNTHETIC\_PROCESS(GO:0045017) |  | 17 | 0.33 | 1.27 | 0.000 | 0.351 | 1.000 | 11286 | tags=76%, list=50%, signal=154% |
| 422 | GLYCEROPHOSPHOLIPID\_BIOSYNTHETIC\_PROCESS(GO:0046474) |  | 17 | 0.33 | 1.27 | 0.000 | 0.350 | 1.000 | 11286 | tags=76%, list=50%, signal=154% |
| 423 | REGULATION\_OF\_MOLECULAR\_FUNCTION(GO:0065009) |  | 34 | 0.26 | 1.26 | 0.000 | 0.350 | 1.000 | 14041 | tags=76%, list=63%, signal=205% |
| 424 | ELECTRON\_TRANSFER\_ACTIVITY(GO:0009055) |  | 60 | 0.34 | 1.26 | 0.000 | 0.355 | 1.000 | 9132 | tags=55%, list=41%, signal=93% |
| 425 | REGULATION\_OF\_HYDROLASE\_ACTIVITY(GO:0051336) |  | 24 | 0.29 | 1.26 | 0.000 | 0.355 | 1.000 | 7303 | tags=42%, list=33%, signal=62% |
| 426 | CELLULAR\_AROMATIC\_COMPOUND\_METABOLIC\_PROCESS(GO:0006725) |  | 1408 | 0.23 | 1.26 | 0.000 | 0.354 | 1.000 | 13017 | tags=63%, list=58%, signal=140% |
| 427 | PHOSPHOTRANSFERASE\_ACTIVITY\_\_ALCOHOL\_GROUP\_AS\_ACCEPTOR(GO:0016773) |  | 555 | 0.26 | 1.26 | 0.000 | 0.354 | 1.000 | 11732 | tags=60%, list=52%, signal=123% |
| 428 | PHOSPHATASE\_ACTIVITY(GO:0016791) |  | 112 | 0.27 | 1.26 | 0.000 | 0.356 | 1.000 | 12353 | tags=63%, list=55%, signal=139% |
| 429 | ORGANOPHOSPHATE\_METABOLIC\_PROCESS(GO:0019637) |  | 194 | 0.26 | 1.26 | 0.000 | 0.355 | 1.000 | 9143 | tags=48%, list=41%, signal=80% |
| 430 | SIGNALING\_RECEPTOR\_BINDING(GO:0005102) |  | 187 | 0.42 | 1.26 | 0.174 | 0.355 | 1.000 | 4220 | tags=25%, list=19%, signal=31% |
| 431 | TRANSFERASE\_ACTIVITY\_\_TRANSFERRING\_HEXOSYL\_GROUPS(GO:0016758) |  | 94 | 0.33 | 1.26 | 0.000 | 0.357 | 1.000 | 4867 | tags=30%, list=22%, signal=38% |
| 432 | CYTOPLASMIC\_VESICLE\_PART(GO:0044433) |  | 17 | 0.39 | 1.26 | 0.000 | 0.357 | 1.000 | 10225 | tags=76%, list=46%, signal=141% |
| 433 | CELLULAR\_RESPONSE\_TO\_DNA\_DAMAGE\_STIMULUS(GO:0006974) |  | 78 | 0.25 | 1.26 | 0.163 | 0.357 | 1.000 | 14006 | tags=76%, list=63%, signal=201% |
| 434 | CELLULAR\_RESPONSE\_TO\_STRESS(GO:0033554) |  | 78 | 0.25 | 1.26 | 0.163 | 0.356 | 1.000 | 14006 | tags=76%, list=63%, signal=201% |
| 435 | ORGANOPHOSPHATE\_BIOSYNTHETIC\_PROCESS(GO:0090407) |  | 133 | 0.25 | 1.26 | 0.000 | 0.356 | 1.000 | 9143 | tags=49%, list=41%, signal=82% |
| 436 | NUCLEOTIDE\_BIOSYNTHETIC\_PROCESS(GO:0009165) |  | 105 | 0.28 | 1.26 | 0.000 | 0.358 | 1.000 | 8028 | tags=45%, list=36%, signal=69% |
| 437 | NUCLEOSIDE\_PHOSPHATE\_BIOSYNTHETIC\_PROCESS(GO:1901293) |  | 105 | 0.28 | 1.26 | 0.000 | 0.357 | 1.000 | 8028 | tags=45%, list=36%, signal=69% |
| 438 | KINASE\_ACTIVITY(GO:0016301) |  | 558 | 0.26 | 1.26 | 0.000 | 0.357 | 1.000 | 11732 | tags=59%, list=52%, signal=122% |
| 439 | NUCLEOSIDE\_PHOSPHATE\_METABOLIC\_PROCESS(GO:0006753) |  | 141 | 0.27 | 1.26 | 0.000 | 0.358 | 1.000 | 8028 | tags=45%, list=36%, signal=69% |
| 440 | RIBOSOME(GO:0005840) |  | 520 | 0.37 | 1.25 | 0.175 | 0.358 | 1.000 | 9441 | tags=56%, list=42%, signal=94% |
| 441 | G\_PROTEIN\_BETA\_GAMMA\_SUBUNIT\_COMPLEX\_BINDING(GO:0031683) |  | 18 | 0.32 | 1.25 | 0.198 | 0.358 | 1.000 | 14027 | tags=83%, list=63%, signal=223% |
| 442 | METAL\_ION\_TRANSMEMBRANE\_TRANSPORTER\_ACTIVITY(GO:0046873) |  | 94 | 0.32 | 1.25 | 0.174 | 0.360 | 1.000 | 8566 | tags=40%, list=38%, signal=65% |
| 443 | PROTEIN\_BINDING(GO:0005515) |  | 3321 | 0.25 | 1.25 | 0.000 | 0.363 | 1.000 | 11456 | tags=53%, list=51%, signal=93% |
| 444 | COENZYME\_BINDING(GO:0050662) |  | 124 | 0.26 | 1.25 | 0.000 | 0.364 | 1.000 | 9659 | tags=49%, list=43%, signal=86% |
| 445 | NUCLEOBASE\_CONTAINING\_COMPOUND\_METABOLIC\_PROCESS(GO:0006139) |  | 1380 | 0.23 | 1.25 | 0.000 | 0.365 | 1.000 | 12911 | tags=62%, list=58%, signal=137% |
| 446 | NUCLEOSIDE\_METABOLIC\_PROCESS(GO:0009116) |  | 22 | 0.35 | 1.25 | 0.193 | 0.366 | 1.000 | 12570 | tags=82%, list=56%, signal=186% |
| 447 | GLYCOSYL\_COMPOUND\_METABOLIC\_PROCESS(GO:1901657) |  | 22 | 0.35 | 1.25 | 0.193 | 0.365 | 1.000 | 12570 | tags=82%, list=56%, signal=186% |
| 448 | SOLUTE:SODIUM\_SYMPORTER\_ACTIVITY(GO:0015370) |  | 17 | 0.45 | 1.25 | 0.174 | 0.366 | 1.000 | 5325 | tags=35%, list=24%, signal=46% |
| 449 | INTRINSIC\_COMPONENT\_OF\_MEMBRANE(GO:0031224) |  | 889 | 0.28 | 1.24 | 0.174 | 0.369 | 1.000 | 7941 | tags=37%, list=35%, signal=56% |
| 450 | CELL\_CELL\_SIGNALING(GO:0007267) |  | 33 | 0.49 | 1.24 | 0.000 | 0.369 | 1.000 | 1861 | tags=18%, list=8%, signal=20% |
| 451 | INTEGRAL\_COMPONENT\_OF\_MEMBRANE(GO:0016021) |  | 887 | 0.28 | 1.24 | 0.174 | 0.369 | 1.000 | 7941 | tags=37%, list=35%, signal=56% |
| 452 | ORGANELLE\_SUBCOMPARTMENT(GO:0031984) |  | 47 | 0.32 | 1.24 | 0.000 | 0.370 | 1.000 | 7854 | tags=51%, list=35%, signal=78% |
| 453 | DNA\_INTEGRATION(GO:0015074) |  | 15 | 0.60 | 1.24 | 0.171 | 0.370 | 1.000 | 1060 | tags=13%, list=5%, signal=14% |
| 454 | PROTEIN\_PHOSPHORYLATION(GO:0006468) |  | 471 | 0.27 | 1.24 | 0.000 | 0.370 | 1.000 | 11732 | tags=59%, list=52%, signal=122% |
| 455 | TRANSFERASE\_ACTIVITY\_\_TRANSFERRING\_ACYL\_GROUPS(GO:0016746) |  | 87 | 0.28 | 1.24 | 0.163 | 0.373 | 1.000 | 13299 | tags=75%, list=59%, signal=183% |
| 456 | SYSTEM\_DEVELOPMENT(GO:0048731) |  | 15 | 0.42 | 1.24 | 0.000 | 0.373 | 1.000 | 9948 | tags=53%, list=44%, signal=96% |
| 457 | GOLGI\_ASSOCIATED\_VESICLE(GO:0005798) |  | 15 | 0.37 | 1.24 | 0.000 | 0.377 | 1.000 | 13356 | tags=93%, list=60%, signal=231% |
| 458 | VESICLE\_MEMBRANE(GO:0012506) |  | 15 | 0.37 | 1.24 | 0.000 | 0.376 | 1.000 | 13356 | tags=93%, list=60%, signal=231% |
| 459 | VESICLE\_COAT(GO:0030120) |  | 15 | 0.37 | 1.24 | 0.000 | 0.375 | 1.000 | 13356 | tags=93%, list=60%, signal=231% |
| 460 | COATED\_VESICLE(GO:0030135) |  | 15 | 0.37 | 1.24 | 0.000 | 0.374 | 1.000 | 13356 | tags=93%, list=60%, signal=231% |
| 461 | CYTOPLASMIC\_VESICLE\_MEMBRANE(GO:0030659) |  | 15 | 0.37 | 1.24 | 0.000 | 0.373 | 1.000 | 13356 | tags=93%, list=60%, signal=231% |
| 462 | GOLGI\_ASSOCIATED\_VESICLE\_MEMBRANE(GO:0030660) |  | 15 | 0.37 | 1.24 | 0.000 | 0.372 | 1.000 | 13356 | tags=93%, list=60%, signal=231% |
| 463 | COATED\_VESICLE\_MEMBRANE(GO:0030662) |  | 15 | 0.37 | 1.24 | 0.000 | 0.372 | 1.000 | 13356 | tags=93%, list=60%, signal=231% |
| 464 | PROTEIN\_DEUBIQUITINATION(GO:0016579) |  | 52 | 0.37 | 1.24 | 0.000 | 0.372 | 1.000 | 10725 | tags=71%, list=48%, signal=136% |
| 465 | PROTEIN\_MODIFICATION\_BY\_SMALL\_PROTEIN\_REMOVAL(GO:0070646) |  | 52 | 0.37 | 1.24 | 0.000 | 0.371 | 1.000 | 10725 | tags=71%, list=48%, signal=136% |
| 466 | ORGANELLE\_LOCALIZATION(GO:0051640) |  | 16 | 0.43 | 1.23 | 0.000 | 0.374 | 1.000 | 6554 | tags=50%, list=29%, signal=71% |
| 467 | DIVALENT\_METAL\_ION\_TRANSPORT(GO:0070838) |  | 16 | 0.41 | 1.23 | 0.000 | 0.375 | 1.000 | 2791 | tags=25%, list=12%, signal=29% |
| 468 | DIVALENT\_INORGANIC\_CATION\_TRANSPORT(GO:0072511) |  | 16 | 0.41 | 1.23 | 0.000 | 0.375 | 1.000 | 2791 | tags=25%, list=12%, signal=29% |
| 469 | NUCLEIC\_ACID\_METABOLIC\_PROCESS(GO:0090304) |  | 1217 | 0.23 | 1.23 | 0.000 | 0.375 | 1.000 | 12036 | tags=57%, list=54%, signal=116% |
| 470 | DNA\_REPAIR(GO:0006281) |  | 70 | 0.25 | 1.23 | 0.163 | 0.378 | 1.000 | 14006 | tags=77%, list=63%, signal=205% |
| 471 | NUCLEOBASE\_CONTAINING\_SMALL\_MOLECULE\_METABOLIC\_PROCESS(GO:0055086) |  | 156 | 0.26 | 1.23 | 0.000 | 0.379 | 1.000 | 9015 | tags=48%, list=40%, signal=80% |
| 472 | ENDONUCLEASE\_ACTIVITY(GO:0004519) |  | 28 | 0.40 | 1.23 | 0.000 | 0.379 | 1.000 | 1596 | tags=11%, list=7%, signal=12% |
| 473 | PROTEIN\_PEPTIDYL\_PROLYL\_ISOMERIZATION(GO:0000413) |  | 52 | 0.40 | 1.23 | 0.000 | 0.382 | 1.000 | 11210 | tags=77%, list=50%, signal=154% |
| 474 | PEPTIDYL\_PROLINE\_MODIFICATION(GO:0018208) |  | 52 | 0.40 | 1.23 | 0.000 | 0.382 | 1.000 | 11210 | tags=77%, list=50%, signal=154% |
| 475 | PEPTIDYL\_PROLYL\_CIS\_TRANS\_ISOMERASE\_ACTIVITY(GO:0003755) |  | 52 | 0.40 | 1.23 | 0.000 | 0.381 | 1.000 | 11210 | tags=77%, list=50%, signal=154% |
| 476 | CIS\_TRANS\_ISOMERASE\_ACTIVITY(GO:0016859) |  | 52 | 0.40 | 1.23 | 0.000 | 0.380 | 1.000 | 11210 | tags=77%, list=50%, signal=154% |
| 477 | PSEUDOURIDINE\_SYNTHESIS(GO:0001522) |  | 15 | 0.38 | 1.23 | 0.000 | 0.380 | 1.000 | 5883 | tags=47%, list=26%, signal=63% |
| 478 | RNA\_METABOLIC\_PROCESS(GO:0016070) |  | 1083 | 0.23 | 1.22 | 0.000 | 0.382 | 1.000 | 11344 | tags=53%, list=51%, signal=102% |
| 479 | NON\_MEMBRANE\_BOUNDED\_ORGANELLE(GO:0043228) |  | 812 | 0.33 | 1.22 | 0.175 | 0.386 | 1.000 | 9805 | tags=52%, list=44%, signal=89% |
| 480 | INTRACELLULAR\_NON\_MEMBRANE\_BOUNDED\_ORGANELLE(GO:0043232) |  | 812 | 0.33 | 1.22 | 0.175 | 0.385 | 1.000 | 9805 | tags=52%, list=44%, signal=89% |
| 481 | FATTY\_ACID\_METABOLIC\_PROCESS(GO:0006631) |  | 16 | 0.37 | 1.22 | 0.000 | 0.384 | 1.000 | 5212 | tags=38%, list=23%, signal=49% |
| 482 | ENDOPLASMIC\_RETICULUM(GO:0005783) |  | 54 | 0.29 | 1.22 | 0.000 | 0.390 | 1.000 | 12675 | tags=72%, list=57%, signal=166% |
| 483 | PROTEASOME\_COMPLEX(GO:0000502) |  | 30 | 0.39 | 1.22 | 0.193 | 0.392 | 1.000 | 11026 | tags=73%, list=49%, signal=144% |
| 484 | ENDOPEPTIDASE\_COMPLEX(GO:1905369) |  | 30 | 0.39 | 1.22 | 0.193 | 0.391 | 1.000 | 11026 | tags=73%, list=49%, signal=144% |
| 485 | DEFENSE\_RESPONSE(GO:0006952) |  | 15 | 0.56 | 1.22 | 0.174 | 0.394 | 1.000 | 5702 | tags=33%, list=25%, signal=45% |
| 486 | SIGNAL\_TRANSDUCTION(GO:0007165) |  | 1021 | 0.26 | 1.21 | 0.174 | 0.401 | 1.000 | 6817 | tags=32%, list=30%, signal=44% |
| 487 | CATABOLIC\_PROCESS(GO:0009056) |  | 130 | 0.27 | 1.21 | 0.000 | 0.402 | 1.000 | 13663 | tags=72%, list=61%, signal=184% |
| 488 | PYRIDOXAL\_PHOSPHATE\_BINDING(GO:0030170) |  | 31 | 0.30 | 1.21 | 0.000 | 0.403 | 1.000 | 9564 | tags=55%, list=43%, signal=96% |
| 489 | VITAMIN\_B6\_BINDING(GO:0070279) |  | 31 | 0.30 | 1.21 | 0.000 | 0.402 | 1.000 | 9564 | tags=55%, list=43%, signal=96% |
| 490 | SIALYLTRANSFERASE\_ACTIVITY(GO:0008373) |  | 18 | 0.34 | 1.21 | 0.000 | 0.401 | 1.000 | 10419 | tags=56%, list=47%, signal=104% |
| 491 | NUCLEASE\_ACTIVITY(GO:0004518) |  | 61 | 0.28 | 1.21 | 0.171 | 0.402 | 1.000 | 10752 | tags=56%, list=48%, signal=107% |
| 492 | ENDOPLASMIC\_RETICULUM\_MEMBRANE(GO:0005789) |  | 31 | 0.33 | 1.21 | 0.000 | 0.406 | 1.000 | 12048 | tags=74%, list=54%, signal=160% |
| 493 | ENDOPLASMIC\_RETICULUM\_SUBCOMPARTMENT(GO:0098827) |  | 31 | 0.33 | 1.21 | 0.000 | 0.405 | 1.000 | 12048 | tags=74%, list=54%, signal=160% |
| 494 | ENZYME\_BINDING(GO:0019899) |  | 147 | 0.25 | 1.21 | 0.000 | 0.405 | 1.000 | 13636 | tags=71%, list=61%, signal=180% |
| 495 | METALLOPEPTIDASE\_ACTIVITY(GO:0008237) |  | 121 | 0.29 | 1.21 | 0.174 | 0.405 | 1.000 | 10338 | tags=50%, list=46%, signal=92% |
| 496 | RAS\_GTPASE\_BINDING(GO:0017016) |  | 90 | 0.26 | 1.21 | 0.000 | 0.405 | 1.000 | 12733 | tags=67%, list=57%, signal=154% |
| 497 | SMALL\_GTPASE\_BINDING(GO:0031267) |  | 90 | 0.26 | 1.21 | 0.000 | 0.404 | 1.000 | 12733 | tags=67%, list=57%, signal=154% |
| 498 | INTRACELLULAR\_SIGNAL\_TRANSDUCTION(GO:0035556) |  | 402 | 0.24 | 1.21 | 0.000 | 0.404 | 1.000 | 13241 | tags=65%, list=59%, signal=156% |
| 499 | NADH\_DEHYDROGENASE\_ACTIVITY(GO:0003954) |  | 18 | 0.33 | 1.21 | 0.335 | 0.404 | 1.000 | 9015 | tags=56%, list=40%, signal=93% |
| 500 | SIGNALING(GO:0023052) |  | 1031 | 0.26 | 1.21 | 0.174 | 0.403 | 1.000 | 6817 | tags=32%, list=30%, signal=44% |
| 501 | HEME\_COPPER\_TERMINAL\_OXIDASE\_ACTIVITY(GO:0015002) |  | 36 | 0.36 | 1.20 | 0.175 | 0.403 | 1.000 | 9132 | tags=58%, list=41%, signal=98% |
| 502 | CELL\_COMMUNICATION(GO:0007154) |  | 1033 | 0.26 | 1.20 | 0.174 | 0.403 | 1.000 | 6817 | tags=32%, list=30%, signal=44% |
| 503 | SECRETION(GO:0046903) |  | 19 | 0.33 | 1.20 | 0.174 | 0.403 | 1.000 | 10530 | tags=58%, list=47%, signal=109% |
| 504 | SECRETION\_BY\_CELL(GO:0032940) |  | 19 | 0.33 | 1.20 | 0.174 | 0.402 | 1.000 | 10530 | tags=58%, list=47%, signal=109% |
| 505 | VITAMIN\_BINDING(GO:0019842) |  | 42 | 0.28 | 1.20 | 0.000 | 0.402 | 1.000 | 13411 | tags=71%, list=60%, signal=178% |
| 506 | ENDOPLASMIC\_RETICULUM\_PART(GO:0044432) |  | 32 | 0.33 | 1.20 | 0.000 | 0.402 | 1.000 | 12048 | tags=75%, list=54%, signal=162% |
| 507 | CELLULAR\_RESPONSE\_TO\_STIMULUS(GO:0051716) |  | 1097 | 0.25 | 1.20 | 0.174 | 0.404 | 1.000 | 6817 | tags=32%, list=30%, signal=44% |
| 508 | REGULATION\_OF\_CELL\_GROWTH(GO:0001558) |  | 17 | 0.51 | 1.19 | 0.210 | 0.413 | 1.000 | 2308 | tags=24%, list=10%, signal=26% |
| 509 | CELL\_GROWTH(GO:0016049) |  | 17 | 0.51 | 1.19 | 0.210 | 0.412 | 1.000 | 2308 | tags=24%, list=10%, signal=26% |
| 510 | REGULATION\_OF\_GROWTH(GO:0040008) |  | 17 | 0.51 | 1.19 | 0.210 | 0.411 | 1.000 | 2308 | tags=24%, list=10%, signal=26% |
| 511 | INSULIN\_LIKE\_GROWTH\_FACTOR\_BINDING(GO:0005520) |  | 17 | 0.51 | 1.19 | 0.210 | 0.411 | 1.000 | 2308 | tags=24%, list=10%, signal=26% |
| 512 | NUCLEAR\_OUTER\_MEMBRANE\_ENDOPLASMIC\_RETICULUM\_MEMBRANE\_NETWORK(GO:0042175) |  | 33 | 0.32 | 1.19 | 0.244 | 0.411 | 1.000 | 12048 | tags=73%, list=54%, signal=157% |
| 513 | ION\_CHANNEL\_ACTIVITY(GO:0005216) |  | 181 | 0.38 | 1.19 | 0.174 | 0.418 | 1.000 | 2471 | tags=14%, list=11%, signal=15% |
| 514 | CHANNEL\_ACTIVITY(GO:0015267) |  | 181 | 0.38 | 1.19 | 0.174 | 0.417 | 1.000 | 2471 | tags=14%, list=11%, signal=15% |
| 515 | PASSIVE\_TRANSMEMBRANE\_TRANSPORTER\_ACTIVITY(GO:0022803) |  | 181 | 0.38 | 1.19 | 0.174 | 0.416 | 1.000 | 2471 | tags=14%, list=11%, signal=15% |
| 516 | SUBSTRATE\_SPECIFIC\_CHANNEL\_ACTIVITY(GO:0022838) |  | 181 | 0.38 | 1.19 | 0.174 | 0.415 | 1.000 | 2471 | tags=14%, list=11%, signal=15% |
| 517 | RESPONSE\_TO\_STRESS(GO:0006950) |  | 122 | 0.23 | 1.19 | 0.163 | 0.415 | 1.000 | 14077 | tags=70%, list=63%, signal=186% |
| 518 | PHOSPHORYLATION(GO:0016310) |  | 521 | 0.25 | 1.19 | 0.000 | 0.415 | 1.000 | 11692 | tags=58%, list=52%, signal=118% |
| 519 | PURINE\_RIBONUCLEOTIDE\_BIOSYNTHETIC\_PROCESS(GO:0009152) |  | 76 | 0.29 | 1.19 | 0.175 | 0.415 | 1.000 | 8028 | tags=46%, list=36%, signal=72% |
| 520 | RIBONUCLEOTIDE\_BIOSYNTHETIC\_PROCESS(GO:0009260) |  | 76 | 0.29 | 1.19 | 0.175 | 0.415 | 1.000 | 8028 | tags=46%, list=36%, signal=72% |
| 521 | RIBOSE\_PHOSPHATE\_BIOSYNTHETIC\_PROCESS(GO:0046390) |  | 76 | 0.29 | 1.19 | 0.175 | 0.414 | 1.000 | 8028 | tags=46%, list=36%, signal=72% |
| 522 | REGULATION\_OF\_ACTIN\_FILAMENT\_POLYMERIZATION(GO:0030833) |  | 19 | 0.38 | 1.19 | 0.000 | 0.419 | 1.000 | 11631 | tags=79%, list=52%, signal=164% |
| 523 | REGULATION\_OF\_ACTIN\_FILAMENT\_LENGTH(GO:0030832) |  | 19 | 0.38 | 1.19 | 0.000 | 0.418 | 1.000 | 11631 | tags=79%, list=52%, signal=164% |
| 524 | REGULATION\_OF\_ACTIN\_POLYMERIZATION\_OR\_DEPOLYMERIZATION(GO:0008064) |  | 19 | 0.38 | 1.19 | 0.000 | 0.417 | 1.000 | 11631 | tags=79%, list=52%, signal=164% |
| 525 | ACTIN\_POLYMERIZATION\_OR\_DEPOLYMERIZATION(GO:0008154) |  | 19 | 0.38 | 1.19 | 0.000 | 0.416 | 1.000 | 11631 | tags=79%, list=52%, signal=164% |
| 526 | ACTIN\_FILAMENT\_POLYMERIZATION(GO:0030041) |  | 19 | 0.38 | 1.19 | 0.000 | 0.415 | 1.000 | 11631 | tags=79%, list=52%, signal=164% |
| 527 | REGULATION\_OF\_CELLULAR\_COMPONENT\_SIZE(GO:0032535) |  | 19 | 0.38 | 1.19 | 0.000 | 0.415 | 1.000 | 11631 | tags=79%, list=52%, signal=164% |
| 528 | REGULATION\_OF\_ACTIN\_CYTOSKELETON\_ORGANIZATION(GO:0032956) |  | 19 | 0.38 | 1.19 | 0.000 | 0.414 | 1.000 | 11631 | tags=79%, list=52%, signal=164% |
| 529 | REGULATION\_OF\_ACTIN\_FILAMENT\_BASED\_PROCESS(GO:0032970) |  | 19 | 0.38 | 1.19 | 0.000 | 0.413 | 1.000 | 11631 | tags=79%, list=52%, signal=164% |
| 530 | REGULATION\_OF\_ACTIN\_FILAMENT\_ORGANIZATION(GO:0110053) |  | 19 | 0.38 | 1.19 | 0.000 | 0.412 | 1.000 | 11631 | tags=79%, list=52%, signal=164% |
| 531 | OXIDOREDUCTASE\_ACTIVITY\_\_ACTING\_ON\_THE\_CH\_CH\_GROUP\_OF\_DONORS(GO:0016627) |  | 34 | 0.30 | 1.18 | 0.000 | 0.413 | 1.000 | 9239 | tags=59%, list=41%, signal=100% |
| 532 | NEGATIVE\_REGULATION\_OF\_GENE\_EXPRESSION(GO:0010629) |  | 19 | 0.29 | 1.18 | 0.209 | 0.415 | 1.000 | 14897 | tags=89%, list=66%, signal=267% |
| 533 | EXTRACELLULAR\_REGION\_PART(GO:0044421) |  | 82 | 0.36 | 1.18 | 0.174 | 0.418 | 1.000 | 2226 | tags=17%, list=10%, signal=19% |
| 534 | POLYMERIC\_CYTOSKELETAL\_FIBER(GO:0099513) |  | 96 | 0.42 | 1.18 | 0.000 | 0.418 | 1.000 | 2348 | tags=25%, list=10%, signal=28% |
| 535 | CHROMOSOMAL\_REGION(GO:0098687) |  | 15 | 0.36 | 1.18 | 0.194 | 0.420 | 1.000 | 13389 | tags=93%, list=60%, signal=232% |
| 536 | MEMBRANE\_PROTEIN\_COMPLEX(GO:0098796) |  | 163 | 0.27 | 1.18 | 0.175 | 0.419 | 1.000 | 11572 | tags=60%, list=52%, signal=123% |
| 537 | INTRAMOLECULAR\_TRANSFERASE\_ACTIVITY(GO:0016866) |  | 21 | 0.26 | 1.18 | 0.000 | 0.420 | 1.000 | 8215 | tags=48%, list=37%, signal=75% |
| 538 | ACTIN\_FILAMENT\_ORGANIZATION(GO:0007015) |  | 30 | 0.39 | 1.18 | 0.212 | 0.419 | 1.000 | 10815 | tags=73%, list=48%, signal=142% |
| 539 | GPI\_ANCHOR\_METABOLIC\_PROCESS(GO:0006505) |  | 16 | 0.32 | 1.18 | 0.249 | 0.419 | 1.000 | 11286 | tags=75%, list=50%, signal=151% |
| 540 | HYDROLASE\_ACTIVITY(GO:0016787) |  | 1168 | 0.23 | 1.17 | 0.174 | 0.424 | 1.000 | 10583 | tags=47%, list=47%, signal=85% |
| 541 | MEMBRANE\_COAT(GO:0030117) |  | 32 | 0.29 | 1.17 | 0.000 | 0.424 | 1.000 | 13356 | tags=81%, list=60%, signal=201% |
| 542 | COATED\_MEMBRANE(GO:0048475) |  | 32 | 0.29 | 1.17 | 0.000 | 0.423 | 1.000 | 13356 | tags=81%, list=60%, signal=201% |
| 543 | S\_ADENOSYLMETHIONINE\_DEPENDENT\_METHYLTRANSFERASE\_ACTIVITY(GO:0008757) |  | 20 | 0.30 | 1.17 | 0.245 | 0.423 | 1.000 | 14955 | tags=95%, list=67%, signal=285% |
| 544 | ION\_BINDING(GO:0043167) |  | 2762 | 0.23 | 1.17 | 0.174 | 0.423 | 1.000 | 9676 | tags=44%, list=43%, signal=67% |
| 545 | GTPASE\_BINDING(GO:0051020) |  | 131 | 0.24 | 1.17 | 0.000 | 0.429 | 1.000 | 13636 | tags=69%, list=61%, signal=176% |
| 546 | PHOSPHORIC\_ESTER\_HYDROLASE\_ACTIVITY(GO:0042578) |  | 164 | 0.24 | 1.16 | 0.000 | 0.431 | 1.000 | 12991 | tags=62%, list=58%, signal=147% |
| 547 | NEGATIVE\_REGULATION\_OF\_METABOLIC\_PROCESS(GO:0009892) |  | 28 | 0.28 | 1.16 | 0.209 | 0.432 | 1.000 | 10924 | tags=57%, list=49%, signal=111% |
| 548 | MACROMOLECULE\_METHYLATION(GO:0043414) |  | 19 | 0.33 | 1.16 | 0.000 | 0.432 | 1.000 | 8920 | tags=58%, list=40%, signal=96% |
| 549 | UBIQUITIN\_PROTEIN\_TRANSFERASE\_ACTIVITY(GO:0004842) |  | 41 | 0.30 | 1.16 | 0.000 | 0.432 | 1.000 | 13752 | tags=80%, list=61%, signal=208% |
| 550 | UBIQUITIN\_LIKE\_PROTEIN\_TRANSFERASE\_ACTIVITY(GO:0019787) |  | 41 | 0.30 | 1.16 | 0.000 | 0.431 | 1.000 | 13752 | tags=80%, list=61%, signal=208% |
| 551 | PROTEIN\_METHYLATION(GO:0006479) |  | 16 | 0.32 | 1.16 | 0.000 | 0.431 | 1.000 | 8920 | tags=56%, list=40%, signal=93% |
| 552 | PROTEIN\_ALKYLATION(GO:0008213) |  | 16 | 0.32 | 1.16 | 0.000 | 0.430 | 1.000 | 8920 | tags=56%, list=40%, signal=93% |
| 553 | NEGATIVE\_REGULATION\_OF\_CELLULAR\_PROCESS(GO:0048523) |  | 61 | 0.24 | 1.16 | 0.175 | 0.438 | 1.000 | 10299 | tags=51%, list=46%, signal=94% |
| 554 | ATP\_METABOLIC\_PROCESS(GO:0046034) |  | 61 | 0.31 | 1.16 | 0.175 | 0.439 | 1.000 | 7953 | tags=48%, list=35%, signal=74% |
| 555 | LIPID\_BINDING(GO:0008289) |  | 110 | 0.24 | 1.16 | 0.174 | 0.441 | 1.000 | 9466 | tags=46%, list=42%, signal=80% |
| 556 | INORGANIC\_ANION\_TRANSPORT(GO:0015698) |  | 26 | 0.33 | 1.15 | 0.174 | 0.445 | 1.000 | 12066 | tags=69%, list=54%, signal=150% |
| 557 | MEMBRANE\_ORGANIZATION(GO:0061024) |  | 17 | 0.29 | 1.15 | 0.163 | 0.448 | 1.000 | 13408 | tags=76%, list=60%, signal=190% |
| 558 | PHOSPHOLIPID\_BIOSYNTHETIC\_PROCESS(GO:0008654) |  | 24 | 0.31 | 1.15 | 0.249 | 0.448 | 1.000 | 14872 | tags=96%, list=66%, signal=285% |
| 559 | KERATIN\_FILAMENT(GO:0045095) |  | 20 | 0.56 | 1.15 | 0.204 | 0.449 | 1.000 | 1868 | tags=35%, list=8%, signal=38% |
| 560 | NEGATIVE\_REGULATION\_OF\_BIOLOGICAL\_PROCESS(GO:0048519) |  | 68 | 0.24 | 1.15 | 0.175 | 0.449 | 1.000 | 10578 | tags=51%, list=47%, signal=97% |
| 561 | REGULATION\_OF\_SIGNAL\_TRANSDUCTION(GO:0009966) |  | 126 | 0.23 | 1.15 | 0.349 | 0.449 | 1.000 | 12733 | tags=60%, list=57%, signal=139% |
| 562 | REGULATION\_OF\_CELL\_COMMUNICATION(GO:0010646) |  | 126 | 0.23 | 1.15 | 0.349 | 0.448 | 1.000 | 12733 | tags=60%, list=57%, signal=139% |
| 563 | INTERMEDIATE\_FILAMENT(GO:0005882) |  | 93 | 0.40 | 1.14 | 0.160 | 0.453 | 1.000 | 3118 | tags=27%, list=14%, signal=31% |
| 564 | INTERMEDIATE\_FILAMENT\_CYTOSKELETON(GO:0045111) |  | 93 | 0.40 | 1.14 | 0.160 | 0.452 | 1.000 | 3118 | tags=27%, list=14%, signal=31% |
| 565 | POTASSIUM\_ION\_TRANSPORT(GO:0006813) |  | 27 | 0.43 | 1.14 | 0.197 | 0.459 | 1.000 | 7063 | tags=48%, list=32%, signal=70% |
| 566 | PROTEASOME\_CORE\_COMPLEX(GO:0005839) |  | 23 | 0.41 | 1.14 | 0.193 | 0.459 | 1.000 | 10943 | tags=74%, list=49%, signal=144% |
| 567 | THREONINE\_TYPE\_ENDOPEPTIDASE\_ACTIVITY(GO:0004298) |  | 23 | 0.41 | 1.14 | 0.193 | 0.458 | 1.000 | 10943 | tags=74%, list=49%, signal=144% |
| 568 | THREONINE\_TYPE\_PEPTIDASE\_ACTIVITY(GO:0070003) |  | 23 | 0.41 | 1.14 | 0.193 | 0.457 | 1.000 | 10943 | tags=74%, list=49%, signal=144% |
| 569 | SULFUR\_COMPOUND\_METABOLIC\_PROCESS(GO:0006790) |  | 27 | 0.30 | 1.14 | 0.335 | 0.457 | 1.000 | 13321 | tags=70%, list=59%, signal=173% |
| 570 | PURINE\_NUCLEOSIDE\_TRIPHOSPHATE\_METABOLIC\_PROCESS(GO:0009144) |  | 71 | 0.29 | 1.14 | 0.335 | 0.458 | 1.000 | 8028 | tags=46%, list=36%, signal=72% |
| 571 | RIBONUCLEOSIDE\_TRIPHOSPHATE\_METABOLIC\_PROCESS(GO:0009199) |  | 71 | 0.29 | 1.14 | 0.335 | 0.457 | 1.000 | 8028 | tags=46%, list=36%, signal=72% |
| 572 | PURINE\_RIBONUCLEOSIDE\_TRIPHOSPHATE\_METABOLIC\_PROCESS(GO:0009205) |  | 71 | 0.29 | 1.14 | 0.335 | 0.457 | 1.000 | 8028 | tags=46%, list=36%, signal=72% |
| 573 | SULFOTRANSFERASE\_ACTIVITY(GO:0008146) |  | 38 | 0.43 | 1.14 | 0.000 | 0.457 | 1.000 | 7312 | tags=47%, list=33%, signal=70% |
| 574 | SUPRAMOLECULAR\_COMPLEX(GO:0099080) |  | 107 | 0.41 | 1.14 | 0.175 | 0.460 | 1.000 | 2348 | tags=24%, list=10%, signal=27% |
| 575 | SUPRAMOLECULAR\_POLYMER(GO:0099081) |  | 107 | 0.41 | 1.14 | 0.175 | 0.459 | 1.000 | 2348 | tags=24%, list=10%, signal=27% |
| 576 | SUPRAMOLECULAR\_FIBER(GO:0099512) |  | 107 | 0.41 | 1.14 | 0.175 | 0.458 | 1.000 | 2348 | tags=24%, list=10%, signal=27% |
| 577 | SMALL\_GTPASE\_MEDIATED\_SIGNAL\_TRANSDUCTION(GO:0007264) |  | 259 | 0.23 | 1.14 | 0.174 | 0.458 | 1.000 | 11627 | tags=56%, list=52%, signal=115% |
| 578 | GPI\_ANCHOR\_BIOSYNTHETIC\_PROCESS(GO:0006506) |  | 15 | 0.31 | 1.13 | 0.249 | 0.465 | 1.000 | 11286 | tags=73%, list=50%, signal=148% |
| 579 | PROTEIN\_LIPIDATION(GO:0006497) |  | 15 | 0.31 | 1.13 | 0.249 | 0.464 | 1.000 | 11286 | tags=73%, list=50%, signal=148% |
| 580 | PHOSPHATIDYLINOSITOL\_BIOSYNTHETIC\_PROCESS(GO:0006661) |  | 15 | 0.31 | 1.13 | 0.249 | 0.463 | 1.000 | 11286 | tags=73%, list=50%, signal=148% |
| 581 | LIPOPROTEIN\_BIOSYNTHETIC\_PROCESS(GO:0042158) |  | 15 | 0.31 | 1.13 | 0.249 | 0.463 | 1.000 | 11286 | tags=73%, list=50%, signal=148% |
| 582 | REGULATION\_OF\_SIGNALING(GO:0023051) |  | 127 | 0.23 | 1.13 | 0.349 | 0.462 | 1.000 | 12733 | tags=60%, list=57%, signal=138% |
| 583 | EXOCYTOSIS(GO:0006887) |  | 18 | 0.32 | 1.13 | 0.174 | 0.462 | 1.000 | 10530 | tags=56%, list=47%, signal=105% |
| 584 | REGULATION\_OF\_RESPONSE\_TO\_STIMULUS(GO:0048583) |  | 132 | 0.23 | 1.13 | 0.349 | 0.463 | 1.000 | 11721 | tags=54%, list=52%, signal=112% |
| 585 | DNA\_DEPENDENT\_ATPASE\_ACTIVITY(GO:0008094) |  | 17 | 0.33 | 1.13 | 0.000 | 0.464 | 1.000 | 12133 | tags=76%, list=54%, signal=167% |
| 586 | PURINE\_NUCLEOSIDE\_MONOPHOSPHATE\_METABOLIC\_PROCESS(GO:0009126) |  | 65 | 0.34 | 1.13 | 0.000 | 0.477 | 1.000 | 7953 | tags=51%, list=35%, signal=78% |
| 587 | RIBONUCLEOSIDE\_MONOPHOSPHATE\_METABOLIC\_PROCESS(GO:0009161) |  | 65 | 0.34 | 1.13 | 0.000 | 0.476 | 1.000 | 7953 | tags=51%, list=35%, signal=78% |
| 588 | PURINE\_RIBONUCLEOSIDE\_MONOPHOSPHATE\_METABOLIC\_PROCESS(GO:0009167) |  | 65 | 0.34 | 1.13 | 0.000 | 0.475 | 1.000 | 7953 | tags=51%, list=35%, signal=78% |
| 589 | PROTON\_TRANSPORTING\_TWO\_SECTOR\_ATPASE\_COMPLEX\_\_CATALYTIC\_DOMAIN(GO:0033178) |  | 17 | 0.35 | 1.12 | 0.337 | 0.477 | 1.000 | 8663 | tags=53%, list=39%, signal=86% |
| 590 | MONOCARBOXYLIC\_ACID\_METABOLIC\_PROCESS(GO:0032787) |  | 41 | 0.28 | 1.12 | 0.349 | 0.476 | 1.000 | 5212 | tags=29%, list=23%, signal=38% |
| 591 | PROTEIN\_METHYLTRANSFERASE\_ACTIVITY(GO:0008276) |  | 15 | 0.29 | 1.12 | 0.245 | 0.480 | 1.000 | 14955 | tags=93%, list=67%, signal=281% |
| 592 | LYASE\_ACTIVITY(GO:0016829) |  | 63 | 0.25 | 1.12 | 0.163 | 0.489 | 1.000 | 8185 | tags=43%, list=37%, signal=67% |
| 593 | ORGANIC\_SUBSTANCE\_CATABOLIC\_PROCESS(GO:1901575) |  | 120 | 0.26 | 1.12 | 0.335 | 0.491 | 1.000 | 13663 | tags=70%, list=61%, signal=178% |
| 594 | TETRAPYRROLE\_METABOLIC\_PROCESS(GO:0033013) |  | 15 | 0.38 | 1.11 | 0.244 | 0.496 | 1.000 | 8185 | tags=60%, list=37%, signal=94% |
| 595 | NUCLEOSIDE\_MONOPHOSPHATE\_METABOLIC\_PROCESS(GO:0009123) |  | 66 | 0.34 | 1.11 | 0.000 | 0.497 | 1.000 | 7953 | tags=50%, list=35%, signal=77% |
| 596 | PYRIMIDINE\_NUCLEOTIDE\_METABOLIC\_PROCESS(GO:0006220) |  | 20 | 0.34 | 1.11 | 0.403 | 0.498 | 1.000 | 11025 | tags=60%, list=49%, signal=118% |
| 597 | MEMBRANE\_LIPID\_METABOLIC\_PROCESS(GO:0006643) |  | 26 | 0.32 | 1.11 | 0.408 | 0.499 | 1.000 | 9143 | tags=54%, list=41%, signal=91% |
| 598 | CARBOXYLIC\_ESTER\_HYDROLASE\_ACTIVITY(GO:0052689) |  | 16 | 0.36 | 1.11 | 0.000 | 0.499 | 1.000 | 7209 | tags=50%, list=32%, signal=74% |
| 599 | OXIDOREDUCTASE\_ACTIVITY(GO:0016491) |  | 502 | 0.23 | 1.10 | 0.174 | 0.506 | 1.000 | 9304 | tags=41%, list=42%, signal=68% |
| 600 | LIGAND\_GATED\_CATION\_CHANNEL\_ACTIVITY(GO:0099094) |  | 21 | 0.48 | 1.10 | 0.337 | 0.505 | 1.000 | 3326 | tags=29%, list=15%, signal=34% |
| 601 | THIOL\_DEPENDENT\_UBIQUITINYL\_HYDROLASE\_ACTIVITY(GO:0036459) |  | 56 | 0.38 | 1.10 | 0.000 | 0.507 | 1.000 | 10725 | tags=73%, list=48%, signal=140% |
| 602 | UBIQUITINYL\_HYDROLASE\_ACTIVITY(GO:0101005) |  | 56 | 0.38 | 1.10 | 0.000 | 0.506 | 1.000 | 10725 | tags=73%, list=48%, signal=140% |
| 603 | EXOPEPTIDASE\_ACTIVITY(GO:0008238) |  | 37 | 0.28 | 1.10 | 0.175 | 0.508 | 1.000 | 10506 | tags=51%, list=47%, signal=97% |
| 604 | OXIDOREDUCTASE\_ACTIVITY\_\_ACTING\_ON\_PEROXIDE\_AS\_ACCEPTOR(GO:0016684) |  | 22 | 0.28 | 1.10 | 0.334 | 0.507 | 1.000 | 15662 | tags=95%, list=70%, signal=317% |
| 605 | POTASSIUM\_CHANNEL\_ACTIVITY(GO:0005267) |  | 29 | 0.40 | 1.10 | 0.337 | 0.507 | 1.000 | 2146 | tags=21%, list=10%, signal=23% |
| 606 | POTASSIUM\_ION\_TRANSMEMBRANE\_TRANSPORTER\_ACTIVITY(GO:0015079) |  | 29 | 0.40 | 1.10 | 0.337 | 0.506 | 1.000 | 2146 | tags=21%, list=10%, signal=23% |
| 607 | ALPHA\_AMINO\_ACID\_METABOLIC\_PROCESS(GO:1901605) |  | 22 | 0.41 | 1.10 | 0.401 | 0.506 | 1.000 | 3753 | tags=27%, list=17%, signal=33% |
| 608 | UBIQUITIN\_LIKE\_PROTEIN\_SPECIFIC\_PROTEASE\_ACTIVITY(GO:0019783) |  | 57 | 0.38 | 1.10 | 0.000 | 0.510 | 1.000 | 10725 | tags=74%, list=48%, signal=141% |
| 609 | CYTOCHROME\_C\_OXIDASE\_ACTIVITY(GO:0004129) |  | 32 | 0.37 | 1.09 | 0.419 | 0.511 | 1.000 | 9132 | tags=59%, list=41%, signal=100% |
| 610 | OXIDOREDUCTASE\_ACTIVITY\_\_ACTING\_ON\_A\_HEME\_GROUP\_OF\_DONORS(GO:0016675) |  | 32 | 0.37 | 1.09 | 0.419 | 0.511 | 1.000 | 9132 | tags=59%, list=41%, signal=100% |
| 611 | OXIDOREDUCTASE\_ACTIVITY\_\_ACTING\_ON\_A\_HEME\_GROUP\_OF\_DONORS\_\_OXYGEN\_AS\_ACCEPTOR(GO:0016676) |  | 32 | 0.37 | 1.09 | 0.419 | 0.510 | 1.000 | 9132 | tags=59%, list=41%, signal=100% |
| 612 | NEGATIVE\_REGULATION\_OF\_BIOSYNTHETIC\_PROCESS(GO:0009890) |  | 16 | 0.33 | 1.09 | 0.209 | 0.511 | 1.000 | 6852 | tags=44%, list=31%, signal=63% |
| 613 | TUBULIN\_BINDING(GO:0015631) |  | 63 | 0.22 | 1.09 | 0.174 | 0.513 | 1.000 | 16935 | tags=92%, list=76%, signal=376% |
| 614 | NADH\_DEHYDROGENASE\_(UBIQUINONE)\_ACTIVITY(GO:0008137) |  | 17 | 0.31 | 1.09 | 0.509 | 0.520 | 1.000 | 9015 | tags=53%, list=40%, signal=89% |
| 615 | OXIDOREDUCTASE\_ACTIVITY\_\_ACTING\_ON\_NAD(P)H\_\_QUINONE\_OR\_SIMILAR\_COMPOUND\_AS\_ACCEPTOR(GO:0016655) |  | 17 | 0.31 | 1.09 | 0.509 | 0.519 | 1.000 | 9015 | tags=53%, list=40%, signal=89% |
| 616 | NADH\_DEHYDROGENASE\_(QUINONE)\_ACTIVITY(GO:0050136) |  | 17 | 0.31 | 1.09 | 0.509 | 0.518 | 1.000 | 9015 | tags=53%, list=40%, signal=89% |
| 617 | GOLGI\_APPARATUS(GO:0005794) |  | 54 | 0.29 | 1.09 | 0.174 | 0.518 | 1.000 | 5086 | tags=30%, list=23%, signal=38% |
| 618 | ATPASE\_ACTIVITY(GO:0016887) |  | 96 | 0.22 | 1.09 | 0.163 | 0.518 | 1.000 | 12210 | tags=59%, list=55%, signal=130% |
| 619 | GROWTH\_FACTOR\_BINDING(GO:0019838) |  | 19 | 0.45 | 1.09 | 0.210 | 0.517 | 1.000 | 2308 | tags=21%, list=10%, signal=23% |
| 620 | TRANSFERASE\_ACTIVITY\_\_TRANSFERRING\_ACYL\_GROUPS\_OTHER\_THAN\_AMINO\_ACYL\_GROUPS(GO:0016747) |  | 48 | 0.27 | 1.09 | 0.163 | 0.517 | 1.000 | 13154 | tags=75%, list=59%, signal=181% |
| 621 | RESPONSE\_TO\_STIMULUS(GO:0050896) |  | 1262 | 0.23 | 1.08 | 0.349 | 0.518 | 1.000 | 6817 | tags=30%, list=30%, signal=41% |
| 622 | SULFURIC\_ESTER\_HYDROLASE\_ACTIVITY(GO:0008484) |  | 16 | 0.28 | 1.08 | 0.421 | 0.520 | 1.000 | 13587 | tags=75%, list=61%, signal=190% |
| 623 | SIGNAL\_TRANSDUCER\_ACTIVITY(GO:0004871) |  | 336 | 0.31 | 1.08 | 0.174 | 0.523 | 1.000 | 7570 | tags=37%, list=34%, signal=55% |
| 624 | METALLOCARBOXYPEPTIDASE\_ACTIVITY(GO:0004181) |  | 16 | 0.38 | 1.08 | 0.413 | 0.522 | 1.000 | 1037 | tags=19%, list=5%, signal=20% |
| 625 | METALLOEXOPEPTIDASE\_ACTIVITY(GO:0008235) |  | 16 | 0.38 | 1.08 | 0.413 | 0.521 | 1.000 | 1037 | tags=19%, list=5%, signal=20% |
| 626 | ORGANIC\_CYCLIC\_COMPOUND\_BIOSYNTHETIC\_PROCESS(GO:1901362) |  | 1042 | 0.20 | 1.08 | 0.337 | 0.521 | 1.000 | 11179 | tags=49%, list=50%, signal=94% |
| 627 | HETEROCYCLE\_BIOSYNTHETIC\_PROCESS(GO:0018130) |  | 1037 | 0.20 | 1.08 | 0.337 | 0.526 | 1.000 | 11179 | tags=49%, list=50%, signal=93% |
| 628 | GALACTOSYLTRANSFERASE\_ACTIVITY(GO:0008378) |  | 16 | 0.42 | 1.07 | 0.349 | 0.530 | 1.000 | 7253 | tags=44%, list=32%, signal=65% |
| 629 | ENDOPEPTIDASE\_ACTIVITY(GO:0004175) |  | 229 | 0.26 | 1.07 | 0.509 | 0.534 | 1.000 | 8152 | tags=36%, list=36%, signal=56% |
| 630 | TRANSMEMBRANE\_RECEPTOR\_PROTEIN\_KINASE\_ACTIVITY(GO:0019199) |  | 18 | 0.41 | 1.07 | 0.000 | 0.533 | 1.000 | 9552 | tags=78%, list=43%, signal=135% |
| 631 | CATALYTIC\_ACTIVITY\_\_ACTING\_ON\_DNA(GO:0140097) |  | 50 | 0.23 | 1.07 | 0.349 | 0.535 | 1.000 | 6776 | tags=28%, list=30%, signal=40% |
| 632 | TRANSFERASE\_ACTIVITY\_\_TRANSFERRING\_PENTOSYL\_GROUPS(GO:0016763) |  | 23 | 0.32 | 1.07 | 0.356 | 0.535 | 1.000 | 12375 | tags=74%, list=55%, signal=165% |
| 633 | CELL\_DEATH(GO:0008219) |  | 58 | 0.22 | 1.07 | 0.349 | 0.535 | 1.000 | 13179 | tags=62%, list=59%, signal=150% |
| 634 | PURINE\_NUCLEOSIDE\_MONOPHOSPHATE\_BIOSYNTHETIC\_PROCESS(GO:0009127) |  | 53 | 0.33 | 1.07 | 0.211 | 0.536 | 1.000 | 7893 | tags=49%, list=35%, signal=76% |
| 635 | RIBONUCLEOSIDE\_MONOPHOSPHATE\_BIOSYNTHETIC\_PROCESS(GO:0009156) |  | 53 | 0.33 | 1.07 | 0.211 | 0.535 | 1.000 | 7893 | tags=49%, list=35%, signal=76% |
| 636 | PURINE\_RIBONUCLEOSIDE\_MONOPHOSPHATE\_BIOSYNTHETIC\_PROCESS(GO:0009168) |  | 53 | 0.33 | 1.07 | 0.211 | 0.534 | 1.000 | 7893 | tags=49%, list=35%, signal=76% |
| 637 | ANTIOXIDANT\_ACTIVITY(GO:0016209) |  | 23 | 0.27 | 1.07 | 0.337 | 0.533 | 1.000 | 15662 | tags=91%, list=70%, signal=303% |
| 638 | PHOSPHORIC\_DIESTER\_HYDROLASE\_ACTIVITY(GO:0008081) |  | 47 | 0.25 | 1.07 | 0.337 | 0.533 | 1.000 | 6443 | tags=34%, list=29%, signal=48% |
| 639 | AROMATIC\_COMPOUND\_BIOSYNTHETIC\_PROCESS(GO:0019438) |  | 1029 | 0.20 | 1.06 | 0.337 | 0.533 | 1.000 | 11179 | tags=49%, list=50%, signal=93% |
| 640 | APOPTOTIC\_PROCESS(GO:0006915) |  | 56 | 0.22 | 1.06 | 0.349 | 0.539 | 1.000 | 13179 | tags=63%, list=59%, signal=151% |
| 641 | PROGRAMMED\_CELL\_DEATH(GO:0012501) |  | 56 | 0.22 | 1.06 | 0.349 | 0.539 | 1.000 | 13179 | tags=63%, list=59%, signal=151% |
| 642 | PHOSPHOTRANSFERASE\_ACTIVITY\_\_PHOSPHATE\_GROUP\_AS\_ACCEPTOR(GO:0016776) |  | 18 | 0.32 | 1.06 | 0.163 | 0.538 | 1.000 | 11367 | tags=61%, list=51%, signal=124% |
| 643 | TRANSMEMBRANE\_RECEPTOR\_PROTEIN\_TYROSINE\_KINASE\_SIGNALING\_PATHWAY(GO:0007169) |  | 17 | 0.31 | 1.06 | 0.417 | 0.542 | 1.000 | 11154 | tags=59%, list=50%, signal=117% |
| 644 | GLYCOLIPID\_METABOLIC\_PROCESS(GO:0006664) |  | 21 | 0.30 | 1.06 | 0.408 | 0.542 | 1.000 | 9143 | tags=52%, list=41%, signal=88% |
| 645 | LIPOSACCHARIDE\_METABOLIC\_PROCESS(GO:1903509) |  | 21 | 0.30 | 1.06 | 0.408 | 0.541 | 1.000 | 9143 | tags=52%, list=41%, signal=88% |
| 646 | TRANSFERASE\_ACTIVITY\_\_TRANSFERRING\_SULFUR\_CONTAINING\_GROUPS(GO:0016782) |  | 42 | 0.40 | 1.06 | 0.000 | 0.540 | 1.000 | 7312 | tags=45%, list=33%, signal=67% |
| 647 | ATPASE\_ACTIVITY\_\_COUPLED\_TO\_TRANSMEMBRANE\_MOVEMENT\_OF\_SUBSTANCES(GO:0042626) |  | 42 | 0.23 | 1.06 | 0.338 | 0.542 | 1.000 | 12560 | tags=62%, list=56%, signal=141% |
| 648 | PRIMARY\_ACTIVE\_TRANSMEMBRANE\_TRANSPORTER\_ACTIVITY(GO:0015399) |  | 42 | 0.23 | 1.06 | 0.338 | 0.542 | 1.000 | 12560 | tags=62%, list=56%, signal=141% |
| 649 | P\_P\_BOND\_HYDROLYSIS\_DRIVEN\_TRANSMEMBRANE\_TRANSPORTER\_ACTIVITY(GO:0015405) |  | 42 | 0.23 | 1.06 | 0.338 | 0.541 | 1.000 | 12560 | tags=62%, list=56%, signal=141% |
| 650 | ATPASE\_ACTIVITY\_\_COUPLED\_TO\_MOVEMENT\_OF\_SUBSTANCES(GO:0043492) |  | 42 | 0.23 | 1.06 | 0.338 | 0.540 | 1.000 | 12560 | tags=62%, list=56%, signal=141% |
| 651 | GTPASE\_ACTIVITY(GO:0003924) |  | 69 | 0.31 | 1.06 | 0.349 | 0.540 | 1.000 | 4804 | tags=28%, list=21%, signal=35% |
| 652 | MICROTUBULE\_CYTOSKELETON\_ORGANIZATION(GO:0000226) |  | 27 | 0.26 | 1.06 | 0.338 | 0.540 | 1.000 | 5307 | tags=30%, list=24%, signal=39% |
| 653 | REGULATION\_OF\_CELLULAR\_PROCESS(GO:0050794) |  | 1960 | 0.21 | 1.05 | 0.174 | 0.540 | 1.000 | 11232 | tags=48%, list=50%, signal=89% |
| 654 | REGULATION\_OF\_BIOLOGICAL\_PROCESS(GO:0050789) |  | 1979 | 0.21 | 1.05 | 0.174 | 0.540 | 1.000 | 11232 | tags=48%, list=50%, signal=88% |
| 655 | IRON\_SULFUR\_CLUSTER\_BINDING(GO:0051536) |  | 30 | 0.29 | 1.05 | 0.245 | 0.544 | 1.000 | 14486 | tags=83%, list=65%, signal=235% |
| 656 | METAL\_CLUSTER\_BINDING(GO:0051540) |  | 30 | 0.29 | 1.05 | 0.245 | 0.543 | 1.000 | 14486 | tags=83%, list=65%, signal=235% |
| 657 | PROTEIN\_TYROSINE\_PHOSPHATASE\_ACTIVITY(GO:0004725) |  | 41 | 0.29 | 1.05 | 0.415 | 0.546 | 1.000 | 8358 | tags=41%, list=37%, signal=66% |
| 658 | COENZYME\_METABOLIC\_PROCESS(GO:0006732) |  | 56 | 0.30 | 1.05 | 0.404 | 0.545 | 1.000 | 7696 | tags=43%, list=34%, signal=65% |
| 659 | NUCLEOSIDE\_MONOPHOSPHATE\_BIOSYNTHETIC\_PROCESS(GO:0009124) |  | 54 | 0.32 | 1.05 | 0.404 | 0.545 | 1.000 | 7893 | tags=48%, list=35%, signal=74% |
| 660 | GLYCOLIPID\_BIOSYNTHETIC\_PROCESS(GO:0009247) |  | 19 | 0.31 | 1.05 | 0.408 | 0.546 | 1.000 | 11286 | tags=63%, list=50%, signal=127% |
| 661 | MEMBRANE\_LIPID\_BIOSYNTHETIC\_PROCESS(GO:0046467) |  | 19 | 0.31 | 1.05 | 0.408 | 0.545 | 1.000 | 11286 | tags=63%, list=50%, signal=127% |
| 662 | REGULATION\_OF\_INTRACELLULAR\_SIGNAL\_TRANSDUCTION(GO:1902531) |  | 98 | 0.22 | 1.05 | 0.520 | 0.546 | 1.000 | 12733 | tags=61%, list=57%, signal=141% |
| 663 | BIOLOGICAL\_REGULATION(GO:0065007) |  | 2021 | 0.21 | 1.05 | 0.174 | 0.547 | 1.000 | 11232 | tags=48%, list=50%, signal=88% |
| 664 | RHO\_GTPASE\_BINDING(GO:0017048) |  | 77 | 0.22 | 1.04 | 0.349 | 0.550 | 1.000 | 13636 | tags=66%, list=61%, signal=169% |
| 665 | RIBONUCLEASE\_ACTIVITY(GO:0004540) |  | 31 | 0.30 | 1.04 | 0.345 | 0.555 | 1.000 | 12945 | tags=71%, list=58%, signal=168% |
| 666 | REGULATION\_OF\_SMALL\_GTPASE\_MEDIATED\_SIGNAL\_TRANSDUCTION(GO:0051056) |  | 91 | 0.22 | 1.04 | 0.520 | 0.554 | 1.000 | 13586 | tags=66%, list=61%, signal=167% |
| 667 | HYDROLASE\_ACTIVITY\_\_ACTING\_ON\_ACID\_ANHYDRIDES(GO:0016817) |  | 299 | 0.21 | 1.04 | 0.174 | 0.554 | 1.000 | 14522 | tags=67%, list=65%, signal=188% |
| 668 | LIGAND\_GATED\_ION\_CHANNEL\_ACTIVITY(GO:0015276) |  | 62 | 0.37 | 1.04 | 0.174 | 0.553 | 1.000 | 4483 | tags=24%, list=20%, signal=30% |
| 669 | LIGAND\_GATED\_CHANNEL\_ACTIVITY(GO:0022834) |  | 62 | 0.37 | 1.04 | 0.174 | 0.553 | 1.000 | 4483 | tags=24%, list=20%, signal=30% |
| 670 | NUCLEOBASE\_CONTAINING\_COMPOUND\_BIOSYNTHETIC\_PROCESS(GO:0034654) |  | 1012 | 0.20 | 1.03 | 0.337 | 0.558 | 1.000 | 11134 | tags=48%, list=50%, signal=92% |
| 671 | RNA\_MODIFICATION(GO:0009451) |  | 26 | 0.29 | 1.03 | 0.511 | 0.559 | 1.000 | 8806 | tags=54%, list=39%, signal=89% |
| 672 | G\_PROTEIN\_COUPLED\_RECEPTOR\_SIGNALING\_PATHWAY(GO:0007186) |  | 280 | 0.31 | 1.03 | 0.174 | 0.559 | 1.000 | 7570 | tags=37%, list=34%, signal=55% |
| 673 | NUCLEOSIDE\_TRIPHOSPHATE\_METABOLIC\_PROCESS(GO:0009141) |  | 78 | 0.29 | 1.03 | 0.614 | 0.560 | 1.000 | 8028 | tags=46%, list=36%, signal=72% |
| 674 | RAS\_PROTEIN\_SIGNAL\_TRANSDUCTION(GO:0007265) |  | 80 | 0.22 | 1.03 | 0.520 | 0.565 | 1.000 | 13586 | tags=65%, list=61%, signal=165% |
| 675 | REGULATION\_OF\_RAS\_PROTEIN\_SIGNAL\_TRANSDUCTION(GO:0046578) |  | 80 | 0.22 | 1.03 | 0.520 | 0.564 | 1.000 | 13586 | tags=65%, list=61%, signal=165% |
| 676 | COFACTOR\_BIOSYNTHETIC\_PROCESS(GO:0051188) |  | 59 | 0.29 | 1.03 | 0.404 | 0.563 | 1.000 | 7696 | tags=42%, list=34%, signal=64% |
| 677 | HYDROLASE\_ACTIVITY\_\_ACTING\_ON\_ACID\_ANHYDRIDES\_\_IN\_PHOSPHORUS\_CONTAINING\_ANHYDRIDES(GO:0016818) |  | 289 | 0.21 | 1.03 | 0.174 | 0.564 | 1.000 | 14522 | tags=66%, list=65%, signal=186% |
| 678 | NEGATIVE\_REGULATION\_OF\_CELL\_CYCLE(GO:0045786) |  | 18 | 0.25 | 1.03 | 0.417 | 0.564 | 1.000 | 3838 | tags=22%, list=17%, signal=27% |
| 679 | REGULATION\_OF\_BIOLOGICAL\_QUALITY(GO:0065008) |  | 104 | 0.27 | 1.03 | 0.337 | 0.564 | 1.000 | 10722 | tags=56%, list=48%, signal=106% |
| 680 | PEROXIDASE\_ACTIVITY(GO:0004601) |  | 15 | 0.27 | 1.02 | 0.174 | 0.567 | 1.000 | 15662 | tags=93%, list=70%, signal=310% |
| 681 | METALLOENDOPEPTIDASE\_ACTIVITY(GO:0004222) |  | 90 | 0.26 | 1.02 | 0.174 | 0.567 | 1.000 | 7425 | tags=33%, list=33%, signal=50% |
| 682 | N\_ACYLTRANSFERASE\_ACTIVITY(GO:0016410) |  | 31 | 0.29 | 1.02 | 0.323 | 0.566 | 1.000 | 13154 | tags=77%, list=59%, signal=187% |
| 683 | PROTEIN\_SERINE\_THREONINE\_KINASE\_ACTIVITY(GO:0004674) |  | 78 | 0.31 | 1.02 | 0.508 | 0.568 | 1.000 | 11732 | tags=69%, list=52%, signal=145% |
| 684 | GUANYL\_NUCLEOTIDE\_EXCHANGE\_FACTOR\_ACTIVITY(GO:0005085) |  | 108 | 0.21 | 1.02 | 0.520 | 0.568 | 1.000 | 13586 | tags=64%, list=61%, signal=162% |
| 685 | SIGNALING\_RECEPTOR\_ACTIVITY(GO:0038023) |  | 281 | 0.31 | 1.02 | 0.174 | 0.569 | 1.000 | 6510 | tags=32%, list=29%, signal=45% |
| 686 | MOLECULAR\_TRANSDUCER\_ACTIVITY(GO:0060089) |  | 281 | 0.31 | 1.02 | 0.174 | 0.568 | 1.000 | 6510 | tags=32%, list=29%, signal=45% |
| 687 | ENDORIBONUCLEASE\_ACTIVITY(GO:0004521) |  | 21 | 0.34 | 1.02 | 0.411 | 0.570 | 1.000 | 12945 | tags=76%, list=58%, signal=180% |
| 688 | PYROPHOSPHATASE\_ACTIVITY(GO:0016462) |  | 281 | 0.21 | 1.02 | 0.174 | 0.569 | 1.000 | 10222 | tags=44%, list=46%, signal=79% |
| 689 | PYRIMIDINE\_CONTAINING\_COMPOUND\_BIOSYNTHETIC\_PROCESS(GO:0072528) |  | 15 | 0.30 | 1.02 | 0.403 | 0.569 | 1.000 | 15477 | tags=93%, list=69%, signal=302% |
| 690 | CARBOXYPEPTIDASE\_ACTIVITY(GO:0004180) |  | 19 | 0.34 | 1.02 | 0.413 | 0.569 | 1.000 | 1037 | tags=16%, list=5%, signal=17% |
| 691 | INORGANIC\_ANION\_TRANSMEMBRANE\_TRANSPORTER\_ACTIVITY(GO:0015103) |  | 24 | 0.31 | 1.01 | 0.508 | 0.575 | 1.000 | 12066 | tags=67%, list=54%, signal=144% |
| 692 | RNA\_BIOSYNTHETIC\_PROCESS(GO:0032774) |  | 896 | 0.20 | 1.01 | 0.508 | 0.574 | 1.000 | 12036 | tags=52%, list=54%, signal=109% |
| 693 | CHROMATIN\_BINDING(GO:0003682) |  | 36 | 0.32 | 1.01 | 0.267 | 0.576 | 1.000 | 13529 | tags=78%, list=60%, signal=196% |
| 694 | TRANSCRIPTION\_\_DNA\_TEMPLATED(GO:0006351) |  | 892 | 0.20 | 1.01 | 0.508 | 0.577 | 1.000 | 12036 | tags=52%, list=54%, signal=108% |
| 695 | NUCLEIC\_ACID\_TEMPLATED\_TRANSCRIPTION(GO:0097659) |  | 892 | 0.20 | 1.01 | 0.508 | 0.576 | 1.000 | 12036 | tags=52%, list=54%, signal=108% |
| 696 | EXTRACELLULAR\_REGION(GO:0005576) |  | 239 | 0.31 | 1.01 | 0.174 | 0.576 | 1.000 | 4220 | tags=20%, list=19%, signal=24% |
| 697 | REGULATION\_OF\_APOPTOTIC\_PROCESS(GO:0042981) |  | 45 | 0.21 | 1.01 | 0.337 | 0.576 | 1.000 | 13179 | tags=60%, list=59%, signal=145% |
| 698 | REGULATION\_OF\_CELL\_DEATH(GO:0010941) |  | 45 | 0.21 | 1.01 | 0.337 | 0.576 | 1.000 | 13179 | tags=60%, list=59%, signal=145% |
| 699 | REGULATION\_OF\_PROGRAMMED\_CELL\_DEATH(GO:0043067) |  | 45 | 0.21 | 1.01 | 0.337 | 0.575 | 1.000 | 13179 | tags=60%, list=59%, signal=145% |
| 700 | NUCLEOSIDE\_TRIPHOSPHATASE\_REGULATOR\_ACTIVITY(GO:0060589) |  | 62 | 0.22 | 1.00 | 0.349 | 0.578 | 1.000 | 15576 | tags=77%, list=70%, signal=253% |
| 701 | PYRIMIDINE\_CONTAINING\_COMPOUND\_METABOLIC\_PROCESS(GO:0072527) |  | 23 | 0.29 | 1.00 | 0.403 | 0.581 | 1.000 | 11981 | tags=61%, list=53%, signal=131% |
| 702 | G\_PROTEIN\_COUPLED\_RECEPTOR\_ACTIVITY(GO:0004930) |  | 229 | 0.32 | 1.00 | 0.174 | 0.586 | 1.000 | 7570 | tags=36%, list=34%, signal=54% |
| 703 | CARBOXY\_LYASE\_ACTIVITY(GO:0016831) |  | 16 | 0.27 | 1.00 | 0.346 | 0.588 | 1.000 | 14325 | tags=81%, list=64%, signal=225% |
| 704 | MOLECULAR\_FUNCTION\_REGULATOR(GO:0098772) |  | 370 | 0.22 | 0.99 | 0.349 | 0.600 | 1.000 | 4006 | tags=17%, list=18%, signal=21% |
| 705 | TRANSMEMBRANE\_SIGNALING\_RECEPTOR\_ACTIVITY(GO:0004888) |  | 277 | 0.30 | 0.99 | 0.174 | 0.600 | 1.000 | 6510 | tags=31%, list=29%, signal=44% |
| 706 | PYRIMIDINE\_NUCLEOSIDE\_TRIPHOSPHATE\_METABOLIC\_PROCESS(GO:0009147) |  | 16 | 0.30 | 0.99 | 0.334 | 0.603 | 1.000 | 11025 | tags=56%, list=49%, signal=111% |
| 707 | ACETYLGLUCOSAMINYLTRANSFERASE\_ACTIVITY(GO:0008375) |  | 17 | 0.25 | 0.99 | 0.509 | 0.607 | 1.000 | 16304 | tags=94%, list=73%, signal=345% |
| 708 | ENZYME\_LINKED\_RECEPTOR\_PROTEIN\_SIGNALING\_PATHWAY(GO:0007167) |  | 25 | 0.25 | 0.99 | 0.520 | 0.608 | 1.000 | 5923 | tags=32%, list=26%, signal=43% |
| 709 | CYCLIC\_NUCLEOTIDE\_BIOSYNTHETIC\_PROCESS(GO:0009190) |  | 17 | 0.26 | 0.98 | 0.398 | 0.618 | 1.000 | 6118 | tags=35%, list=27%, signal=49% |
| 710 | PHOSPHORUS\_OXYGEN\_LYASE\_ACTIVITY(GO:0016849) |  | 17 | 0.26 | 0.98 | 0.398 | 0.617 | 1.000 | 6118 | tags=35%, list=27%, signal=49% |
| 711 | OXIDATION\_REDUCTION\_PROCESS(GO:0055114) |  | 418 | 0.20 | 0.98 | 0.497 | 0.623 | 1.000 | 15243 | tags=69%, list=68%, signal=212% |
| 712 | COFACTOR\_BINDING(GO:0048037) |  | 237 | 0.20 | 0.98 | 0.337 | 0.622 | 1.000 | 15479 | tags=70%, list=69%, signal=223% |
| 713 | NUCLEOSIDE\_TRIPHOSPHATASE\_ACTIVITY(GO:0017111) |  | 275 | 0.20 | 0.98 | 0.174 | 0.622 | 1.000 | 14522 | tags=65%, list=65%, signal=184% |
| 714 | RNA\_SPLICING(GO:0008380) |  | 20 | 0.27 | 0.97 | 0.404 | 0.623 | 1.000 | 12881 | tags=70%, list=57%, signal=165% |
| 715 | NUCLEOSOME\_BINDING(GO:0031491) |  | 26 | 0.38 | 0.97 | 0.493 | 0.633 | 1.000 | 3518 | tags=35%, list=16%, signal=41% |
| 716 | MICROTUBULE\_BINDING(GO:0008017) |  | 55 | 0.22 | 0.96 | 0.411 | 0.640 | 1.000 | 16935 | tags=91%, list=76%, signal=372% |
| 717 | SODIUM\_ION\_TRANSMEMBRANE\_TRANSPORTER\_ACTIVITY(GO:0015081) |  | 31 | 0.33 | 0.96 | 0.334 | 0.640 | 1.000 | 7751 | tags=32%, list=35%, signal=49% |
| 718 | METAL\_ION\_BINDING(GO:0046872) |  | 1521 | 0.19 | 0.96 | 0.508 | 0.649 | 1.000 | 8012 | tags=31%, list=36%, signal=45% |
| 719 | CARBOHYDRATE\_BINDING(GO:0030246) |  | 46 | 0.21 | 0.96 | 0.509 | 0.651 | 1.000 | 8267 | tags=33%, list=37%, signal=52% |
| 720 | SERINE\_TYPE\_ENDOPEPTIDASE\_ACTIVITY(GO:0004252) |  | 91 | 0.28 | 0.96 | 0.509 | 0.650 | 1.000 | 5741 | tags=27%, list=26%, signal=37% |
| 721 | OXIDOREDUCTASE\_ACTIVITY\_\_ACTING\_ON\_A\_SULFUR\_GROUP\_OF\_DONORS(GO:0016667) |  | 16 | 0.24 | 0.96 | 0.404 | 0.651 | 1.000 | 10942 | tags=56%, list=49%, signal=110% |
| 722 | RESPONSE\_TO\_OXIDATIVE\_STRESS(GO:0006979) |  | 17 | 0.24 | 0.96 | 0.337 | 0.652 | 1.000 | 15662 | tags=88%, list=70%, signal=293% |
| 723 | N\_ACETYLTRANSFERASE\_ACTIVITY(GO:0008080) |  | 23 | 0.28 | 0.96 | 0.163 | 0.652 | 1.000 | 13154 | tags=74%, list=59%, signal=179% |
| 724 | CATION\_BINDING(GO:0043169) |  | 1525 | 0.19 | 0.96 | 0.508 | 0.653 | 1.000 | 8012 | tags=31%, list=36%, signal=45% |
| 725 | FLAVIN\_ADENINE\_DINUCLEOTIDE\_BINDING(GO:0050660) |  | 40 | 0.24 | 0.95 | 0.602 | 0.653 | 1.000 | 14429 | tags=75%, list=64%, signal=210% |
| 726 | REGULATION\_OF\_MACROMOLECULE\_METABOLIC\_PROCESS(GO:0060255) |  | 841 | 0.19 | 0.95 | 0.508 | 0.654 | 1.000 | 15274 | tags=69%, list=68%, signal=210% |
| 727 | REGULATION\_OF\_METABOLIC\_PROCESS(GO:0019222) |  | 850 | 0.18 | 0.95 | 0.508 | 0.654 | 1.000 | 15274 | tags=69%, list=68%, signal=209% |
| 728 | CELL\_REDOX\_HOMEOSTASIS(GO:0045454) |  | 43 | 0.34 | 0.95 | 0.508 | 0.654 | 1.000 | 10707 | tags=63%, list=48%, signal=120% |
| 729 | REGULATION\_OF\_CELLULAR\_METABOLIC\_PROCESS(GO:0031323) |  | 841 | 0.18 | 0.95 | 0.508 | 0.654 | 1.000 | 14828 | tags=66%, list=66%, signal=189% |
| 730 | COFACTOR\_METABOLIC\_PROCESS(GO:0051186) |  | 85 | 0.26 | 0.95 | 0.404 | 0.654 | 1.000 | 8983 | tags=46%, list=40%, signal=76% |
| 731 | REGULATION\_OF\_NITROGEN\_COMPOUND\_METABOLIC\_PROCESS(GO:0051171) |  | 834 | 0.19 | 0.95 | 0.508 | 0.659 | 1.000 | 14828 | tags=66%, list=66%, signal=189% |
| 732 | REGULATION\_OF\_PRIMARY\_METABOLIC\_PROCESS(GO:0080090) |  | 834 | 0.19 | 0.95 | 0.508 | 0.659 | 1.000 | 14828 | tags=66%, list=66%, signal=189% |
| 733 | MICROTUBULE\_CYTOSKELETON(GO:0015630) |  | 41 | 0.21 | 0.95 | 0.508 | 0.659 | 1.000 | 12012 | tags=54%, list=54%, signal=115% |
| 734 | REGULATION\_OF\_MACROMOLECULE\_BIOSYNTHETIC\_PROCESS(GO:0010556) |  | 817 | 0.19 | 0.95 | 0.508 | 0.660 | 1.000 | 15274 | tags=69%, list=68%, signal=210% |
| 735 | REGULATION\_OF\_CELLULAR\_BIOSYNTHETIC\_PROCESS(GO:0031326) |  | 817 | 0.19 | 0.95 | 0.508 | 0.659 | 1.000 | 15274 | tags=69%, list=68%, signal=210% |
| 736 | REGULATION\_OF\_CELLULAR\_MACROMOLECULE\_BIOSYNTHETIC\_PROCESS(GO:2000112) |  | 817 | 0.19 | 0.95 | 0.508 | 0.658 | 1.000 | 15274 | tags=69%, list=68%, signal=210% |
| 737 | GATED\_CHANNEL\_ACTIVITY(GO:0022836) |  | 89 | 0.29 | 0.95 | 0.337 | 0.659 | 1.000 | 4693 | tags=21%, list=21%, signal=27% |
| 738 | ION\_GATED\_CHANNEL\_ACTIVITY(GO:0022839) |  | 89 | 0.29 | 0.95 | 0.337 | 0.658 | 1.000 | 4693 | tags=21%, list=21%, signal=27% |
| 739 | REGULATION\_OF\_BIOSYNTHETIC\_PROCESS(GO:0009889) |  | 818 | 0.19 | 0.94 | 0.508 | 0.660 | 1.000 | 15274 | tags=69%, list=68%, signal=210% |
| 740 | REGULATION\_OF\_RHO\_PROTEIN\_SIGNAL\_TRANSDUCTION(GO:0035023) |  | 67 | 0.21 | 0.94 | 0.520 | 0.661 | 1.000 | 13586 | tags=63%, list=61%, signal=159% |
| 741 | RHO\_PROTEIN\_SIGNAL\_TRANSDUCTION(GO:0007266) |  | 67 | 0.21 | 0.94 | 0.520 | 0.661 | 1.000 | 13586 | tags=63%, list=61%, signal=159% |
| 742 | RHO\_GUANYL\_NUCLEOTIDE\_EXCHANGE\_FACTOR\_ACTIVITY(GO:0005089) |  | 67 | 0.21 | 0.94 | 0.520 | 0.660 | 1.000 | 13586 | tags=63%, list=61%, signal=159% |
| 743 | RAS\_GUANYL\_NUCLEOTIDE\_EXCHANGE\_FACTOR\_ACTIVITY(GO:0005088) |  | 67 | 0.21 | 0.94 | 0.520 | 0.659 | 1.000 | 13586 | tags=63%, list=61%, signal=159% |
| 744 | REGULATION\_OF\_NUCLEOBASE\_CONTAINING\_COMPOUND\_METABOLIC\_PROCESS(GO:0019219) |  | 813 | 0.18 | 0.94 | 0.508 | 0.659 | 1.000 | 15274 | tags=69%, list=68%, signal=210% |
| 745 | REGULATION\_OF\_GENE\_EXPRESSION(GO:0010468) |  | 821 | 0.18 | 0.94 | 0.508 | 0.659 | 1.000 | 15274 | tags=69%, list=68%, signal=210% |
| 746 | CELLULAR\_MODIFIED\_AMINO\_ACID\_METABOLIC\_PROCESS(GO:0006575) |  | 19 | 0.37 | 0.94 | 0.503 | 0.659 | 1.000 | 13321 | tags=79%, list=59%, signal=195% |
| 747 | EXTRINSIC\_COMPONENT\_OF\_MEMBRANE(GO:0019898) |  | 20 | 0.32 | 0.94 | 0.349 | 0.658 | 1.000 | 12683 | tags=70%, list=57%, signal=161% |
| 748 | CYTOSKELETON(GO:0005856) |  | 204 | 0.24 | 0.94 | 0.349 | 0.658 | 1.000 | 10749 | tags=46%, list=48%, signal=88% |
| 749 | REGULATION\_OF\_TRANSCRIPTION\_\_DNA\_TEMPLATED(GO:0006355) |  | 806 | 0.18 | 0.94 | 0.508 | 0.663 | 1.000 | 15274 | tags=69%, list=68%, signal=210% |
| 750 | REGULATION\_OF\_NUCLEIC\_ACID\_TEMPLATED\_TRANSCRIPTION(GO:1903506) |  | 806 | 0.18 | 0.94 | 0.508 | 0.662 | 1.000 | 15274 | tags=69%, list=68%, signal=210% |
| 751 | REGULATION\_OF\_RNA\_BIOSYNTHETIC\_PROCESS(GO:2001141) |  | 806 | 0.18 | 0.94 | 0.508 | 0.661 | 1.000 | 15274 | tags=69%, list=68%, signal=210% |
| 752 | COENZYME\_BIOSYNTHETIC\_PROCESS(GO:0009108) |  | 51 | 0.27 | 0.94 | 0.614 | 0.661 | 1.000 | 7696 | tags=39%, list=34%, signal=60% |
| 753 | HETEROTRIMERIC\_G\_PROTEIN\_COMPLEX(GO:0005834) |  | 15 | 0.32 | 0.94 | 0.520 | 0.663 | 1.000 | 11791 | tags=67%, list=53%, signal=141% |
| 754 | CYTOPLASMIC\_SIDE\_OF\_PLASMA\_MEMBRANE(GO:0009898) |  | 15 | 0.32 | 0.94 | 0.520 | 0.662 | 1.000 | 11791 | tags=67%, list=53%, signal=141% |
| 755 | EXTRINSIC\_COMPONENT\_OF\_PLASMA\_MEMBRANE(GO:0019897) |  | 15 | 0.32 | 0.94 | 0.520 | 0.662 | 1.000 | 11791 | tags=67%, list=53%, signal=141% |
| 756 | EXTRINSIC\_COMPONENT\_OF\_CYTOPLASMIC\_SIDE\_OF\_PLASMA\_MEMBRANE(GO:0031234) |  | 15 | 0.32 | 0.94 | 0.520 | 0.661 | 1.000 | 11791 | tags=67%, list=53%, signal=141% |
| 757 | SIDE\_OF\_MEMBRANE(GO:0098552) |  | 15 | 0.32 | 0.94 | 0.520 | 0.660 | 1.000 | 11791 | tags=67%, list=53%, signal=141% |
| 758 | CYTOPLASMIC\_SIDE\_OF\_MEMBRANE(GO:0098562) |  | 15 | 0.32 | 0.94 | 0.520 | 0.659 | 1.000 | 11791 | tags=67%, list=53%, signal=141% |
| 759 | GTPASE\_COMPLEX(GO:1905360) |  | 15 | 0.32 | 0.94 | 0.520 | 0.658 | 1.000 | 11791 | tags=67%, list=53%, signal=141% |
| 760 | REGULATION\_OF\_RNA\_METABOLIC\_PROCESS(GO:0051252) |  | 808 | 0.18 | 0.94 | 0.508 | 0.659 | 1.000 | 15274 | tags=69%, list=68%, signal=210% |
| 761 | NUCLEOBASE\_CONTAINING\_COMPOUND\_KINASE\_ACTIVITY(GO:0019205) |  | 18 | 0.28 | 0.93 | 0.668 | 0.663 | 1.000 | 11367 | tags=56%, list=51%, signal=113% |
| 762 | ATP\_BIOSYNTHETIC\_PROCESS(GO:0006754) |  | 49 | 0.29 | 0.93 | 0.614 | 0.669 | 1.000 | 7893 | tags=45%, list=35%, signal=69% |
| 763 | CYTOSKELETAL\_PART(GO:0044430) |  | 185 | 0.25 | 0.93 | 0.509 | 0.673 | 1.000 | 5222 | tags=25%, list=23%, signal=32% |
| 764 | NUCLEAR\_CHROMOSOME(GO:0000228) |  | 15 | 0.22 | 0.93 | 0.520 | 0.674 | 1.000 | 13123 | tags=67%, list=59%, signal=161% |
| 765 | 3'\_5'\_CYCLIC\_NUCLEOTIDE\_PHOSPHODIESTERASE\_ACTIVITY(GO:0004114) |  | 21 | 0.34 | 0.92 | 0.507 | 0.680 | 1.000 | 6386 | tags=38%, list=29%, signal=53% |
| 766 | ENZYME\_ACTIVATOR\_ACTIVITY(GO:0008047) |  | 54 | 0.23 | 0.91 | 0.524 | 0.695 | 1.000 | 15308 | tags=80%, list=68%, signal=251% |
| 767 | ACETYLTRANSFERASE\_ACTIVITY(GO:0016407) |  | 24 | 0.26 | 0.91 | 0.338 | 0.701 | 1.000 | 13154 | tags=71%, list=59%, signal=171% |
| 768 | CELLULAR\_HOMEOSTASIS(GO:0019725) |  | 58 | 0.30 | 0.91 | 0.508 | 0.701 | 1.000 | 10707 | tags=57%, list=48%, signal=109% |
| 769 | SERINE\_TYPE\_PEPTIDASE\_ACTIVITY(GO:0008236) |  | 103 | 0.26 | 0.91 | 0.509 | 0.703 | 1.000 | 7897 | tags=34%, list=35%, signal=52% |
| 770 | SERINE\_HYDROLASE\_ACTIVITY(GO:0017171) |  | 103 | 0.26 | 0.91 | 0.509 | 0.702 | 1.000 | 7897 | tags=34%, list=35%, signal=52% |
| 771 | DOUBLE\_STRANDED\_DNA\_BINDING(GO:0003690) |  | 16 | 0.34 | 0.90 | 0.680 | 0.708 | 1.000 | 13754 | tags=94%, list=61%, signal=243% |
| 772 | HOMEOSTATIC\_PROCESS(GO:0042592) |  | 61 | 0.29 | 0.90 | 0.508 | 0.708 | 1.000 | 10707 | tags=56%, list=48%, signal=106% |
| 773 | OXIDOREDUCTASE\_ACTIVITY\_\_ACTING\_ON\_THE\_ALDEHYDE\_OR\_OXO\_GROUP\_OF\_DONORS\_\_NAD\_OR\_NADP\_AS\_ACCEPTOR(GO:0016620) |  | 16 | 0.39 | 0.90 | 0.668 | 0.711 | 1.000 | 3378 | tags=25%, list=15%, signal=29% |
| 774 | CYCLIC\_NUCLEOTIDE\_METABOLIC\_PROCESS(GO:0009187) |  | 18 | 0.24 | 0.90 | 0.813 | 0.715 | 1.000 | 6118 | tags=33%, list=27%, signal=46% |
| 775 | CYCLIC\_NUCLEOTIDE\_PHOSPHODIESTERASE\_ACTIVITY(GO:0004112) |  | 22 | 0.31 | 0.89 | 0.507 | 0.721 | 1.000 | 6386 | tags=36%, list=29%, signal=51% |
| 776 | MONOCARBOXYLIC\_ACID\_BIOSYNTHETIC\_PROCESS(GO:0072330) |  | 32 | 0.29 | 0.89 | 0.614 | 0.721 | 1.000 | 5212 | tags=28%, list=23%, signal=37% |
| 777 | ENDORIBONUCLEASE\_ACTIVITY\_\_PRODUCING\_5'\_PHOSPHOMONOESTERS(GO:0016891) |  | 17 | 0.33 | 0.88 | 0.411 | 0.730 | 1.000 | 12671 | tags=71%, list=57%, signal=162% |
| 778 | NEUROTRANSMITTER\_TRANSPORT(GO:0006836) |  | 16 | 0.36 | 0.88 | 0.505 | 0.737 | 1.000 | 1857 | tags=13%, list=8%, signal=14% |
| 779 | NUCLEOSIDE\_TRIPHOSPHATE\_BIOSYNTHETIC\_PROCESS(GO:0009142) |  | 58 | 0.25 | 0.87 | 0.614 | 0.739 | 1.000 | 8028 | tags=43%, list=36%, signal=67% |
| 780 | PURINE\_NUCLEOSIDE\_TRIPHOSPHATE\_BIOSYNTHETIC\_PROCESS(GO:0009145) |  | 58 | 0.25 | 0.87 | 0.614 | 0.738 | 1.000 | 8028 | tags=43%, list=36%, signal=67% |
| 781 | RIBONUCLEOSIDE\_TRIPHOSPHATE\_BIOSYNTHETIC\_PROCESS(GO:0009201) |  | 58 | 0.25 | 0.87 | 0.614 | 0.737 | 1.000 | 8028 | tags=43%, list=36%, signal=67% |
| 782 | PURINE\_RIBONUCLEOSIDE\_TRIPHOSPHATE\_BIOSYNTHETIC\_PROCESS(GO:0009206) |  | 58 | 0.25 | 0.87 | 0.614 | 0.736 | 1.000 | 8028 | tags=43%, list=36%, signal=67% |
| 783 | MICROTUBULE\_BASED\_PROCESS(GO:0007017) |  | 89 | 0.19 | 0.87 | 0.683 | 0.738 | 1.000 | 17003 | tags=84%, list=76%, signal=348% |
| 784 | MICROTUBULE\_ASSOCIATED\_COMPLEX(GO:0005875) |  | 23 | 0.24 | 0.87 | 0.683 | 0.738 | 1.000 | 2815 | tags=17%, list=13%, signal=20% |
| 785 | GTPASE\_ACTIVATOR\_ACTIVITY(GO:0005096) |  | 47 | 0.21 | 0.86 | 0.524 | 0.760 | 1.000 | 15308 | tags=77%, list=68%, signal=241% |
| 786 | CATION\_CHANNEL\_ACTIVITY(GO:0005261) |  | 62 | 0.30 | 0.84 | 0.672 | 0.774 | 1.000 | 7912 | tags=32%, list=35%, signal=50% |
| 787 | CELL\_CYCLE\_PROCESS(GO:0022402) |  | 30 | 0.19 | 0.84 | 0.520 | 0.774 | 1.000 | 15993 | tags=77%, list=71%, signal=268% |
| 788 | ENDONUCLEASE\_ACTIVITY\_\_ACTIVE\_WITH\_EITHER\_RIBO\_\_OR\_DEOXYRIBONUCLEIC\_ACIDS\_AND\_PRODUCING\_5'\_PHOSPHOMONOESTERS(GO:0016893) |  | 18 | 0.29 | 0.83 | 0.411 | 0.785 | 1.000 | 12671 | tags=67%, list=57%, signal=153% |
| 789 | NUCLEOSOMAL\_DNA\_BINDING(GO:0031492) |  | 24 | 0.32 | 0.83 | 0.760 | 0.796 | 1.000 | 3319 | tags=29%, list=15%, signal=34% |
| 790 | CHROMATIN\_DNA\_BINDING(GO:0031490) |  | 24 | 0.32 | 0.83 | 0.760 | 0.795 | 1.000 | 3319 | tags=29%, list=15%, signal=34% |
| 791 | ACTIN\_CYTOSKELETON(GO:0015629) |  | 57 | 0.22 | 0.82 | 0.349 | 0.803 | 1.000 | 11699 | tags=51%, list=52%, signal=106% |
| 792 | ENZYME\_REGULATOR\_ACTIVITY(GO:0030234) |  | 141 | 0.19 | 0.82 | 0.509 | 0.807 | 1.000 | 14627 | tags=62%, list=65%, signal=179% |
| 793 | EXTRACELLULAR\_LIGAND\_GATED\_ION\_CHANNEL\_ACTIVITY(GO:0005230) |  | 49 | 0.28 | 0.82 | 0.680 | 0.809 | 1.000 | 4483 | tags=18%, list=20%, signal=23% |
| 794 | FERRIC\_IRON\_BINDING(GO:0008199) |  | 15 | 0.26 | 0.81 | 0.506 | 0.819 | 1.000 | 15673 | tags=80%, list=70%, signal=266% |
| 795 | LIPOPROTEIN\_METABOLIC\_PROCESS(GO:0042157) |  | 26 | 0.20 | 0.80 | 0.672 | 0.833 | 1.000 | 11328 | tags=50%, list=51%, signal=101% |
| 796 | EXTRACELLULAR\_SPACE(GO:0005615) |  | 24 | 0.30 | 0.80 | 0.497 | 0.833 | 1.000 | 7370 | tags=25%, list=33%, signal=37% |
| 797 | SCAVENGER\_RECEPTOR\_ACTIVITY(GO:0005044) |  | 22 | 0.27 | 0.80 | 0.843 | 0.838 | 1.000 | 7779 | tags=32%, list=35%, signal=49% |
| 798 | CARGO\_RECEPTOR\_ACTIVITY(GO:0038024) |  | 22 | 0.27 | 0.80 | 0.843 | 0.837 | 1.000 | 7779 | tags=32%, list=35%, signal=49% |
| 799 | HYDROLASE\_ACTIVITY\_\_ACTING\_ON\_CARBON\_NITROGEN\_(BUT\_NOT\_PEPTIDE)\_BONDS(GO:0016810) |  | 43 | 0.19 | 0.79 | 0.509 | 0.847 | 1.000 | 14760 | tags=63%, list=66%, signal=184% |
| 800 | RNA\_SPLICING\_\_VIA\_TRANSESTERIFICATION\_REACTIONS(GO:0000375) |  | 15 | 0.26 | 0.78 | 0.764 | 0.863 | 1.000 | 12662 | tags=67%, list=57%, signal=153% |
| 801 | RNA\_SPLICING\_\_VIA\_TRANSESTERIFICATION\_REACTIONS\_WITH\_BULGED\_ADENOSINE\_AS\_NUCLEOPHILE(GO:0000377) |  | 15 | 0.26 | 0.78 | 0.764 | 0.862 | 1.000 | 12662 | tags=67%, list=57%, signal=153% |
| 802 | MRNA\_SPLICING\_\_VIA\_SPLICEOSOME(GO:0000398) |  | 15 | 0.26 | 0.78 | 0.764 | 0.861 | 1.000 | 12662 | tags=67%, list=57%, signal=153% |
| 803 | CARBOHYDRATE\_METABOLIC\_PROCESS(GO:0005975) |  | 161 | 0.18 | 0.78 | 0.672 | 0.862 | 1.000 | 16292 | tags=72%, list=73%, signal=262% |
| 804 | INTEGRAL\_COMPONENT\_OF\_PLASMA\_MEMBRANE(GO:0005887) |  | 23 | 0.32 | 0.78 | 0.843 | 0.865 | 1.000 | 11520 | tags=43%, list=51%, signal=89% |
| 805 | INTRINSIC\_COMPONENT\_OF\_PLASMA\_MEMBRANE(GO:0031226) |  | 23 | 0.32 | 0.78 | 0.843 | 0.864 | 1.000 | 11520 | tags=43%, list=51%, signal=89% |
| 806 | CYTOKINE\_ACTIVITY(GO:0005125) |  | 33 | 0.26 | 0.77 | 0.497 | 0.866 | 1.000 | 666 | tags=9%, list=3%, signal=9% |
| 807 | HYDROLASE\_ACTIVITY\_\_HYDROLYZING\_O\_GLYCOSYL\_COMPOUNDS(GO:0004553) |  | 57 | 0.18 | 0.76 | 0.509 | 0.880 | 1.000 | 17403 | tags=81%, list=78%, signal=361% |
| 808 | CARBON\_CARBON\_LYASE\_ACTIVITY(GO:0016830) |  | 20 | 0.23 | 0.76 | 0.680 | 0.883 | 1.000 | 14738 | tags=70%, list=66%, signal=204% |
| 809 | GTPASE\_REGULATOR\_ACTIVITY(GO:0030695) |  | 54 | 0.21 | 0.75 | 0.524 | 0.886 | 1.000 | 15576 | tags=76%, list=70%, signal=249% |
| 810 | SERINE\_TYPE\_ENDOPEPTIDASE\_INHIBITOR\_ACTIVITY(GO:0004867) |  | 15 | 0.29 | 0.74 | 0.608 | 0.899 | 1.000 | 10859 | tags=53%, list=48%, signal=103% |
| 811 | MYOSIN\_COMPLEX(GO:0016459) |  | 35 | 0.21 | 0.74 | 0.520 | 0.898 | 1.000 | 14070 | tags=57%, list=63%, signal=153% |
| 812 | MICROTUBULE\_BASED\_MOVEMENT(GO:0007018) |  | 57 | 0.18 | 0.74 | 0.605 | 0.898 | 1.000 | 17003 | tags=82%, list=76%, signal=341% |
| 813 | CALCIUM\_ION\_BINDING(GO:0005509) |  | 274 | 0.18 | 0.73 | 0.680 | 0.903 | 1.000 | 7265 | tags=24%, list=32%, signal=36% |
| 814 | MOTOR\_ACTIVITY(GO:0003774) |  | 89 | 0.16 | 0.73 | 0.520 | 0.903 | 1.000 | 17003 | tags=75%, list=76%, signal=311% |
| 815 | CELL\_DIFFERENTIATION(GO:0030154) |  | 16 | 0.28 | 0.73 | 0.614 | 0.903 | 1.000 | 5955 | tags=25%, list=27%, signal=34% |
| 816 | MICROTUBULE\_MOTOR\_ACTIVITY(GO:0003777) |  | 54 | 0.17 | 0.71 | 0.605 | 0.923 | 1.000 | 17003 | tags=81%, list=76%, signal=337% |
| 817 | COPPER\_ION\_BINDING(GO:0005507) |  | 19 | 0.23 | 0.71 | 0.812 | 0.926 | 1.000 | 14795 | tags=74%, list=66%, signal=217% |
| 818 | HYDROLASE\_ACTIVITY\_\_ACTING\_ON\_GLYCOSYL\_BONDS(GO:0016798) |  | 66 | 0.17 | 0.70 | 0.672 | 0.935 | 1.000 | 17496 | tags=80%, list=78%, signal=366% |
| 819 | CELL\_ADHESION(GO:0007155) |  | 119 | 0.18 | 0.69 | 0.683 | 0.943 | 1.000 | 9062 | tags=30%, list=40%, signal=51% |
| 820 | BIOLOGICAL\_ADHESION(GO:0022610) |  | 119 | 0.18 | 0.69 | 0.683 | 0.942 | 1.000 | 9062 | tags=30%, list=40%, signal=51% |
| 821 | OXIDOREDUCTASE\_ACTIVITY\_\_ACTING\_ON\_SINGLE\_DONORS\_WITH\_INCORPORATION\_OF\_MOLECULAR\_OXYGEN(GO:0016701) |  | 21 | 0.26 | 0.69 | 0.843 | 0.945 | 1.000 | 4765 | tags=19%, list=21%, signal=24% |
| 822 | SODIUM\_ION\_TRANSPORT(GO:0006814) |  | 21 | 0.25 | 0.69 | 0.843 | 0.944 | 1.000 | 7751 | tags=29%, list=35%, signal=44% |
| 823 | CYSTEINE\_TYPE\_ENDOPEPTIDASE\_ACTIVITY(GO:0004197) |  | 15 | 0.24 | 0.69 | 0.764 | 0.944 | 1.000 | 14295 | tags=60%, list=64%, signal=166% |
| 824 | HORMONE\_ACTIVITY(GO:0005179) |  | 35 | 0.28 | 0.69 | 0.843 | 0.943 | 1.000 | 2248 | tags=23%, list=10%, signal=25% |
| 825 | ION\_HOMEOSTASIS(GO:0050801) |  | 16 | 0.24 | 0.68 | 0.683 | 0.950 | 1.000 | 15673 | tags=75%, list=70%, signal=249% |
| 826 | CHEMICAL\_HOMEOSTASIS(GO:0048878) |  | 16 | 0.24 | 0.68 | 0.683 | 0.949 | 1.000 | 15673 | tags=75%, list=70%, signal=249% |
| 827 | METAL\_ION\_HOMEOSTASIS(GO:0055065) |  | 16 | 0.24 | 0.68 | 0.683 | 0.948 | 1.000 | 15673 | tags=75%, list=70%, signal=249% |
| 828 | CATION\_HOMEOSTASIS(GO:0055080) |  | 16 | 0.24 | 0.68 | 0.683 | 0.947 | 1.000 | 15673 | tags=75%, list=70%, signal=249% |
| 829 | INORGANIC\_ION\_HOMEOSTASIS(GO:0098771) |  | 16 | 0.24 | 0.68 | 0.683 | 0.945 | 1.000 | 15673 | tags=75%, list=70%, signal=249% |
| 830 | OXIDOREDUCTASE\_ACTIVITY\_\_ACTING\_ON\_THE\_ALDEHYDE\_OR\_OXO\_GROUP\_OF\_DONORS(GO:0016903) |  | 22 | 0.25 | 0.67 | 0.843 | 0.945 | 1.000 | 5925 | tags=27%, list=26%, signal=37% |
| 831 | NUCLEOSIDE\_DIPHOSPHATE\_METABOLIC\_PROCESS(GO:0009132) |  | 37 | 0.22 | 0.67 | 0.811 | 0.946 | 1.000 | 16493 | tags=84%, list=74%, signal=317% |
| 832 | OXIDOREDUCTASE\_ACTIVITY\_\_ACTING\_ON\_SINGLE\_DONORS\_WITH\_INCORPORATION\_OF\_MOLECULAR\_OXYGEN\_\_INCORPORATION\_OF\_TWO\_ATOMS\_OF\_OXYGEN(GO:0016702) |  | 20 | 0.24 | 0.67 | 0.843 | 0.945 | 1.000 | 2236 | tags=10%, list=10%, signal=11% |
| 833 | CELLULAR\_ION\_HOMEOSTASIS(GO:0006873) |  | 15 | 0.23 | 0.65 | 0.683 | 0.954 | 1.000 | 15673 | tags=73%, list=70%, signal=244% |
| 834 | CELLULAR\_METAL\_ION\_HOMEOSTASIS(GO:0006875) |  | 15 | 0.23 | 0.65 | 0.683 | 0.953 | 1.000 | 15673 | tags=73%, list=70%, signal=244% |
| 835 | CELLULAR\_CATION\_HOMEOSTASIS(GO:0030003) |  | 15 | 0.23 | 0.65 | 0.683 | 0.952 | 1.000 | 15673 | tags=73%, list=70%, signal=244% |
| 836 | CELLULAR\_CHEMICAL\_HOMEOSTASIS(GO:0055082) |  | 15 | 0.23 | 0.65 | 0.683 | 0.951 | 1.000 | 15673 | tags=73%, list=70%, signal=244% |
| 837 | OXIDOREDUCTION\_COENZYME\_METABOLIC\_PROCESS(GO:0006733) |  | 34 | 0.22 | 0.65 | 0.811 | 0.951 | 1.000 | 16493 | tags=85%, list=74%, signal=323% |
| 838 | MONOSACCHARIDE\_METABOLIC\_PROCESS(GO:0005996) |  | 18 | 0.21 | 0.65 | 0.614 | 0.951 | 1.000 | 17188 | tags=89%, list=77%, signal=382% |
| 839 | MULTI\_ORGANISM\_PROCESS(GO:0051704) |  | 35 | 0.21 | 0.65 | 0.843 | 0.950 | 1.000 | 7940 | tags=31%, list=35%, signal=49% |
| 840 | CYTOKINE\_RECEPTOR\_BINDING(GO:0005126) |  | 40 | 0.23 | 0.65 | 0.843 | 0.950 | 1.000 | 3747 | tags=15%, list=17%, signal=18% |
| 841 | MOVEMENT\_OF\_CELL\_OR\_SUBCELLULAR\_COMPONENT(GO:0006928) |  | 67 | 0.16 | 0.65 | 0.605 | 0.952 | 1.000 | 17003 | tags=76%, list=76%, signal=315% |
| 842 | DIOXYGENASE\_ACTIVITY(GO:0051213) |  | 25 | 0.21 | 0.65 | 0.683 | 0.951 | 1.000 | 14154 | tags=60%, list=63%, signal=163% |
| 843 | PYRIDINE\_NUCLEOTIDE\_METABOLIC\_PROCESS(GO:0019362) |  | 31 | 0.21 | 0.64 | 0.811 | 0.952 | 1.000 | 16493 | tags=84%, list=74%, signal=317% |
| 844 | NICOTINAMIDE\_NUCLEOTIDE\_METABOLIC\_PROCESS(GO:0046496) |  | 31 | 0.21 | 0.64 | 0.811 | 0.951 | 1.000 | 16493 | tags=84%, list=74%, signal=317% |
| 845 | PYRIDINE\_CONTAINING\_COMPOUND\_METABOLIC\_PROCESS(GO:0072524) |  | 31 | 0.21 | 0.64 | 0.811 | 0.950 | 1.000 | 16493 | tags=84%, list=74%, signal=317% |
| 846 | NUCLEOSIDE\_DIPHOSPHATE\_PHOSPHORYLATION(GO:0006165) |  | 32 | 0.21 | 0.63 | 0.614 | 0.958 | 1.000 | 16493 | tags=81%, list=74%, signal=308% |
| 847 | NUCLEOTIDE\_PHOSPHORYLATION(GO:0046939) |  | 32 | 0.21 | 0.63 | 0.614 | 0.956 | 1.000 | 16493 | tags=81%, list=74%, signal=308% |
| 848 | IRON\_ION\_BINDING(GO:0005506) |  | 72 | 0.19 | 0.63 | 0.843 | 0.957 | 1.000 | 6473 | tags=22%, list=29%, signal=31% |
| 849 | PLASMA\_MEMBRANE\_PROTEIN\_COMPLEX(GO:0098797) |  | 49 | 0.20 | 0.62 | 0.843 | 0.961 | 1.000 | 7215 | tags=31%, list=32%, signal=45% |
| 850 | AROMATIC\_COMPOUND\_CATABOLIC\_PROCESS(GO:0019439) |  | 42 | 0.18 | 0.62 | 0.672 | 0.963 | 1.000 | 16493 | tags=76%, list=74%, signal=288% |
| 851 | ORGANIC\_CYCLIC\_COMPOUND\_CATABOLIC\_PROCESS(GO:1901361) |  | 42 | 0.18 | 0.62 | 0.672 | 0.962 | 1.000 | 16493 | tags=76%, list=74%, signal=288% |
| 852 | HEXOSE\_METABOLIC\_PROCESS(GO:0019318) |  | 15 | 0.23 | 0.61 | 0.764 | 0.966 | 1.000 | 17188 | tags=100%, list=77%, signal=429% |
| 853 | TRANSMEMBRANE\_TRANSPORTER\_COMPLEX(GO:1902495) |  | 19 | 0.23 | 0.61 | 0.843 | 0.966 | 1.000 | 11520 | tags=42%, list=51%, signal=87% |
| 854 | TRANSPORTER\_COMPLEX(GO:1990351) |  | 19 | 0.23 | 0.61 | 0.843 | 0.965 | 1.000 | 11520 | tags=42%, list=51%, signal=87% |
| 855 | NICOTINAMIDE\_NUCLEOTIDE\_BIOSYNTHETIC\_PROCESS(GO:0019359) |  | 28 | 0.21 | 0.61 | 0.811 | 0.964 | 1.000 | 16493 | tags=82%, list=74%, signal=311% |
| 856 | PYRIDINE\_NUCLEOTIDE\_BIOSYNTHETIC\_PROCESS(GO:0019363) |  | 28 | 0.21 | 0.61 | 0.811 | 0.963 | 1.000 | 16493 | tags=82%, list=74%, signal=311% |
| 857 | PYRIDINE\_CONTAINING\_COMPOUND\_BIOSYNTHETIC\_PROCESS(GO:0072525) |  | 28 | 0.21 | 0.61 | 0.811 | 0.962 | 1.000 | 16493 | tags=82%, list=74%, signal=311% |
| 858 | NUCLEOBASE\_CONTAINING\_SMALL\_MOLECULE\_BIOSYNTHETIC\_PROCESS(GO:0034404) |  | 39 | 0.18 | 0.59 | 0.614 | 0.974 | 1.000 | 16493 | tags=77%, list=74%, signal=291% |
| 859 | CELL\_PERIPHERY(GO:0071944) |  | 74 | 0.18 | 0.59 | 0.843 | 0.974 | 1.000 | 11791 | tags=43%, list=53%, signal=91% |
| 860 | VIRAL\_PROCESS(GO:0016032) |  | 20 | 0.22 | 0.59 | 0.813 | 0.973 | 1.000 | 7122 | tags=25%, list=32%, signal=37% |
| 861 | SYMBIONT\_PROCESS(GO:0044403) |  | 20 | 0.22 | 0.59 | 0.813 | 0.972 | 1.000 | 7122 | tags=25%, list=32%, signal=37% |
| 862 | INTERSPECIES\_INTERACTION\_BETWEEN\_ORGANISMS(GO:0044419) |  | 20 | 0.22 | 0.59 | 0.813 | 0.971 | 1.000 | 7122 | tags=25%, list=32%, signal=37% |
| 863 | NUCLEOBASE\_CONTAINING\_COMPOUND\_CATABOLIC\_PROCESS(GO:0034655) |  | 35 | 0.18 | 0.59 | 0.614 | 0.971 | 1.000 | 16493 | tags=77%, list=74%, signal=292% |
| 864 | ENDOPEPTIDASE\_INHIBITOR\_ACTIVITY(GO:0004866) |  | 37 | 0.19 | 0.58 | 0.843 | 0.973 | 1.000 | 10859 | tags=38%, list=48%, signal=73% |
| 865 | ENDOPEPTIDASE\_REGULATOR\_ACTIVITY(GO:0061135) |  | 37 | 0.19 | 0.58 | 0.843 | 0.972 | 1.000 | 10859 | tags=38%, list=48%, signal=73% |
| 866 | GENERATION\_OF\_PRECURSOR\_METABOLITES\_AND\_ENERGY(GO:0006091) |  | 41 | 0.18 | 0.58 | 0.811 | 0.971 | 1.000 | 16529 | tags=78%, list=74%, signal=297% |
| 867 | HOMOPHILIC\_CELL\_ADHESION\_VIA\_PLASMA\_MEMBRANE\_ADHESION\_MOLECULES(GO:0007156) |  | 72 | 0.17 | 0.58 | 0.843 | 0.975 | 1.000 | 1217 | tags=6%, list=5%, signal=6% |
| 868 | CELL\_CELL\_ADHESION\_VIA\_PLASMA\_MEMBRANE\_ADHESION\_MOLECULES(GO:0098742) |  | 72 | 0.17 | 0.58 | 0.843 | 0.974 | 1.000 | 1217 | tags=6%, list=5%, signal=6% |
| 869 | REPRODUCTION(GO:0000003) |  | 16 | 0.19 | 0.57 | 0.843 | 0.981 | 1.000 | 12197 | tags=56%, list=54%, signal=123% |
| 870 | REPRODUCTIVE\_PROCESS(GO:0022414) |  | 16 | 0.19 | 0.57 | 0.843 | 0.980 | 1.000 | 12197 | tags=56%, list=54%, signal=123% |
| 871 | CELL\_CELL\_ADHESION(GO:0098609) |  | 73 | 0.17 | 0.57 | 0.843 | 0.981 | 1.000 | 1217 | tags=5%, list=5%, signal=6% |
| 872 | PLASMA\_MEMBRANE(GO:0005886) |  | 65 | 0.18 | 0.57 | 0.843 | 0.983 | 1.000 | 11791 | tags=43%, list=53%, signal=91% |
| 873 | CELLULAR\_DEVELOPMENTAL\_PROCESS(GO:0048869) |  | 18 | 0.21 | 0.56 | 0.764 | 0.985 | 1.000 | 10463 | tags=39%, list=47%, signal=73% |
| 874 | PLASMA\_MEMBRANE\_PART(GO:0044459) |  | 59 | 0.18 | 0.54 | 0.843 | 0.988 | 1.000 | 11791 | tags=42%, list=53%, signal=89% |
| 875 | LIPID\_TRANSPORT(GO:0006869) |  | 18 | 0.19 | 0.54 | 0.614 | 0.988 | 1.000 | 2786 | tags=17%, list=12%, signal=19% |
| 876 | RESPONSE\_TO\_BIOTIC\_STIMULUS(GO:0009607) |  | 20 | 0.26 | 0.54 | 0.764 | 0.988 | 1.000 | 3817 | tags=15%, list=17%, signal=18% |
| 877 | ENZYME\_INHIBITOR\_ACTIVITY(GO:0004857) |  | 68 | 0.15 | 0.53 | 0.843 | 0.988 | 1.000 | 10957 | tags=37%, list=49%, signal=72% |
| 878 | GLYCOLYTIC\_PROCESS(GO:0006096) |  | 23 | 0.19 | 0.52 | 0.811 | 0.992 | 1.000 | 16493 | tags=78%, list=74%, signal=296% |
| 879 | PYRUVATE\_METABOLIC\_PROCESS(GO:0006090) |  | 23 | 0.19 | 0.52 | 0.811 | 0.991 | 1.000 | 16493 | tags=78%, list=74%, signal=296% |
| 880 | ATP\_GENERATION\_FROM\_ADP(GO:0006757) |  | 23 | 0.19 | 0.52 | 0.811 | 0.990 | 1.000 | 16493 | tags=78%, list=74%, signal=296% |
| 881 | PURINE\_NUCLEOSIDE\_DIPHOSPHATE\_METABOLIC\_PROCESS(GO:0009135) |  | 23 | 0.19 | 0.52 | 0.811 | 0.989 | 1.000 | 16493 | tags=78%, list=74%, signal=296% |
| 882 | PURINE\_RIBONUCLEOSIDE\_DIPHOSPHATE\_METABOLIC\_PROCESS(GO:0009179) |  | 23 | 0.19 | 0.52 | 0.811 | 0.988 | 1.000 | 16493 | tags=78%, list=74%, signal=296% |
| 883 | RIBONUCLEOSIDE\_DIPHOSPHATE\_METABOLIC\_PROCESS(GO:0009185) |  | 23 | 0.19 | 0.52 | 0.811 | 0.987 | 1.000 | 16493 | tags=78%, list=74%, signal=296% |
| 884 | PYRUVATE\_BIOSYNTHETIC\_PROCESS(GO:0042866) |  | 23 | 0.19 | 0.52 | 0.811 | 0.985 | 1.000 | 16493 | tags=78%, list=74%, signal=296% |
| 885 | ADP\_METABOLIC\_PROCESS(GO:0046031) |  | 23 | 0.19 | 0.52 | 0.811 | 0.984 | 1.000 | 16493 | tags=78%, list=74%, signal=296% |
| 886 | CELLULAR\_NITROGEN\_COMPOUND\_CATABOLIC\_PROCESS(GO:0044270) |  | 40 | 0.18 | 0.51 | 0.811 | 0.985 | 1.000 | 16493 | tags=75%, list=74%, signal=284% |
| 887 | HETEROCYCLE\_CATABOLIC\_PROCESS(GO:0046700) |  | 40 | 0.18 | 0.51 | 0.811 | 0.984 | 1.000 | 16493 | tags=75%, list=74%, signal=284% |
| 888 | PEPTIDASE\_INHIBITOR\_ACTIVITY(GO:0030414) |  | 46 | 0.17 | 0.51 | 0.843 | 0.983 | 1.000 | 10957 | tags=35%, list=49%, signal=68% |
| 889 | PEPTIDASE\_REGULATOR\_ACTIVITY(GO:0061134) |  | 46 | 0.17 | 0.51 | 0.843 | 0.982 | 1.000 | 10957 | tags=35%, list=49%, signal=68% |
| 890 | CARBOHYDRATE\_CATABOLIC\_PROCESS(GO:0016052) |  | 24 | 0.19 | 0.50 | 0.811 | 0.981 | 1.000 | 16493 | tags=79%, list=74%, signal=300% |
| 891 | OXIDOREDUCTASE\_ACTIVITY\_\_ACTING\_ON\_PAIRED\_DONORS\_\_WITH\_INCORPORATION\_OR\_REDUCTION\_OF\_MOLECULAR\_OXYGEN(GO:0016705) |  | 71 | 0.14 | 0.49 | 0.843 | 0.984 | 1.000 | 15323 | tags=54%, list=68%, signal=169% |
| 892 | TETRAPYRROLE\_BINDING(GO:0046906) |  | 79 | 0.13 | 0.42 | 0.843 | 0.995 | 1.000 | 16690 | tags=59%, list=74%, signal=232% |
| 893 | HEME\_BINDING(GO:0020037) |  | 74 | 0.13 | 0.41 | 0.843 | 0.995 | 1.000 | 16690 | tags=59%, list=74%, signal=232% |
| 894 | IMMUNE\_RESPONSE(GO:0006955) |  | 97 | 0.15 | 0.38 | 0.843 | 0.999 | 1.000 | 3926 | tags=14%, list=18%, signal=17% |
| 895 | IMMUNE\_SYSTEM\_PROCESS(GO:0002376) |  | 100 | 0.14 | 0.37 | 0.843 | 0.999 | 1.000 | 3926 | tags=14%, list=18%, signal=17% |
Table: Gene sets enriched in phenotype
 **TE1 (2 samples)
 **[plain text format]****
